# Five new species of *Vibrissina* Rondani (Diptera: Tachinidae) from Area de Conservación Guanacaste in Northwestern Costa Rica

**DOI:** 10.3897/BDJ.5.e10967

**Published:** 2017-07-18

**Authors:** AJ Fleming, D. Monty Wood, M. Alex Smith, Tanya Dapkey, Winnie Hallwachs, Daniel H. Janzen

**Affiliations:** 1 Agriculture Agri-Food Canada, Ottawa, Canada; 2 Department of Integrative Biology and the Biodiversity Institute of Ontario, Guelph, Canada; 3 University of Pennsylvania, Philadelphia, United States of America

**Keywords:** Diptera, Tachinidae, tropical rain forest, tropical dry forest, parasitoid fly, host-specificity, sawfly larvae, Blondeliini

## Abstract

**Background:**

We describe five new species in the genus *Vibrissina* Rondani from Area de Conservación Guanacaste (ACG). All species were reared from wild-caught sawfly larvae (Hymenoptera: Symphyta: Argidae and Tenthredinidae). We provide a morphological description of each species together with information on life history, molecular data, and photographic documentation.

**New information:**

Five new species of *Vibrissina* Rondani: *Vibrissina randycurtisi* **sp. n.**, *V. randyjonesi* **sp. n.**, *V. robertwellsi* **sp. n.**, *V. danmartini* **sp. n.**, *V. hallwachsorum* **sp. n.**

## Introduction

With more than 8,500 described species classified into more than 1,500 genera (Irwin et al. 2003, O'Hara 2016) the family Tachinidae is one of the most diverse of all the insect families. The Neotropical Region has a vast and largely unknown fauna of tachinids, with a majority of names belonging to the Mexican and Brazilian fauna deriving from work conducted by early researchers, and from a limited number of localities (Wood 1985). The last major work on Neotropical Tachinidae, conducted by Guimaraes (1971), catalogued some 2,864 species, a number much larger than that of any other faunal region. Based on what has recently been discovered in Costa Rica (Smith et al. 2007, Fleming et al. 2014a, Fleming et al. 2014b, Fleming et al. 2015c, Fleming et al. 2015a, Fleming et al. 2015b) and what is already present in museum collections, this number is undoubtedly just a small fraction of what actually exists in nature.

The present study is part of an effort to document the tachinid species living within the terrestrial 120,000 km^2^ Area de Conservación Guanacaste (http://www.acguanacaste.ac.cr), and provide names for any new species. A comparison of tachinids collected during the ACG inventory with those present in the national collection in the Museo Nacional de Costa Rica (formerly INBio) shows minimal overlap of species, suggesting that the tachinid fauna in other parts of the country is quite different from that of ACG and requires much additional study (Janzen et al. 2012, Bertrand et al. 2014, Brown et al. 2014, Fernandez-Triana et al. 2014, Phillips-Rodríguez et al. 2014, Fernandez-Triana et al. 2015, Sharkey et al. 2015).

The genus *Vibrissina* Rondani, 1861 (Exoristinae: Blondeliini) originally included Rondani (1861) two nominal species from the Palearctic, *Vibrissina turrita* (Meigen, 1824) and *Vibrissina debilitata* (Pandellé, 1896). Three Asian species were added by Shima (1983), but the genus as a whole remained unstudied until Wood (1985) revised the tribe Blondeliini and 7 other genera under *Vibrissina*, bringing the total number of valid included species to 28. Among the genera synonymized by Wood (1985), *Spathimeigenia* Townsend represented the largest proportion of the newly added species. Almost all members of the genus *Vibrissina* are parasitoids on sawfly larvae in the families Argidae, Diprionidae, and Tenthredinidae (Wood 1985).

*Vibrissina* possesses the following characters, common to the tribe Blondeliini: prosternum setose; first postsutural supraalar bristle shorter than first postsutural dorsocentral bristle; bend of vein M rounded, forming an obtuse angle; subapical scutellar bristles long, stout, and divergent; and veins R_4+5_ and M ending at or near wing tip (Wood 1985).

Wood (1985) made a distinction between New World and Old World species of *Vibrissina*, the Old World species being characterized in large part by a bare parafacial, a character which is rare in New World species. All ACG-reared species of *Vibrissina* are parasitoids of sawfly larvae in the families Diprionidae and Tenthredinidae. This host specificity as parasitoids of sawfly larvae indicate that the various species placed by Wood (1985) under *Vibrissina* may actually form a monophyletic assemblage.

In this paper we describe five new species of *Vibrissina* using morphology and CO1 (cytochrome c oxidase I) gene sequences or “DNA barcodes”, and provide additional information on host preference.

## Materials and methods

### Study area and rearing intensity

All flies and rearing information described here were collected as part of the 37+ year-old and ongoing inventory of caterpillars, their food plants and their parasitoids across the three major ecosystems of the terrestrial portion of Area de Conservación Guanacaste (ACG) in northwestern Costa Rica (Smith et al. 2006, Smith et al. 2007, Smith et al. 2008, Janzen et al. 2009, Janzen and Hallwachs 2011, Rodriguez et al. 2012, Smith et al. 2012, Fleming et al. 2014a, Fleming et al. 2014b, Fleming et al. 2015c, Fleming et al. 2015a, Fleming et al. 2015b). The parasitoid rearing methods are described by Janzen et al. (2009) and at http://janzen.bio.upenn.edu/caterpillars/methodology/how/parasitoid_husbandry.htm.

This inventory has reared more than 750,000 wild-caught caterpillars and sawfly larvae since 1978. All frequencies of parasitization reported here need to be considered against this background inventory (Janzen et al. 2009, Janzen and Hallwachs 2011, Fernandez-Triana et al. 2014). The scope of our treatment of the genus *Vibrissina* is limited to species found within ACG.

It should be noted that this inventory searches some vegetation types and vertical strata much more thoroughly than others. This bias is due to the methods employed for collecting of specimens, which rely solely on those animals within reach of the collectors, up to 3m above the ground. Recent comparisons of reared species of parasitoids with those collected in the same place with hand nets or Malaise traps demonstrate that, to date, the estimated 1,100 species of tachinid flies reared by the inventory represent less than half the species of caterpillar (and caterpillar-like larvae) parasitizing Tachinidae present in ACG. The largest unsampled habitat is the foliage of the canopy that is higher than approximately 3–4 m above the ground.

This paper on *Vibrissina* is part of a larger effort to describe the new species reared during the ACG inventory (Smith et al. 2006, Smith et al. 2007, Smith et al. 2008, Smith et al. 2012, Fleming et al. 2014a, Fleming et al. 2014b, Fleming et al. 2015c, Fleming et al. 2015a, Fleming et al. 2015b, Fleming et al. 2015a, Fleming et al. 2015b, Fleming et al. 2016). This series of papers will represent the baseline for later, more detailed ecological and behavioral accounts and studies that will extend across ACG ecological groups, whole ecosystems, and taxonomic assemblages much larger than a genus

### Imaging

Species accounts presented in this paper are deliberately brief and only include basic descriptions of body morphology and coloration commonly used in the identification of Tachinidae. The descriptions are complemented with a series of color photos of every species, used to illustrate the morphological differences among them. The morphological terminology used follows Cumming and Wood (2009). All dissections and photography were carried out following the methods detailed in O’Hara (1983) and Fleming et al. (2014a). Measurements and examples of parts of the terminalia are illustrated in Fig. 1.

### Acronyms for depositories

CNC Canadian National Collection of Insects, Arachnids and Nematodes, Ottawa, Canada

MACN Museo Argentino de Ciencias Naturales Bernardino Rivadavia, Buenos Aires, Argentina

MNCR Museo Nacional de Costa Rica (formerly Instituto Nacional de Biodiversidad - INBio), Santo Domingo de Heredia, Costa Rica

MRSN Museo Regionale di Scienze Naturali di Torino (collection formerly housed at Museo di Zoologia, Istituto di Zoologia e Anatomia Comparata, Università di Torino - MZUT), Turin, Italy

MZSP Museu de Zoologia da Universidade de São Paulo, São Paulo, Brazil

NHMUK Natural History Museum, London, United Kingdom (formerly British Museum of Natural History)

NHMW Naturhistorisches Museum Wien, Vienna, Austria

USNM National Museum of Natural History, Washington, D.C., U.S.A. (formerly United States National Museum)

### Voucher specimen management

The procedures surrounding the management of voucher specimens has been detailed in previous papers in this series (Fleming et al. 2014a, Fleming et al. 2014b, Fleming et al. 2015a, Fleming et al. 2015b, Fleming et al. 2015c). In brief, caterpillars reared from the ACG efforts receive a unique voucher code in the format yy–SRNP–xxxxx; any parasitoid emerging from a caterpillar receives the same voucher code, and if/when later dealt with as an individual, receives a second unique voucher code in the format DHJPARxxxxxxx. The collateral data for each voucher code are available at: http://janzen.bio.upenn.edu/caterpillars/database.lasso.

All DHJPARxxxxxxx-coded tachinids have had one leg removed and sent for DNA barcoding to the Biodiversity Institute of Ontario (BIO) and its Center for Biodiversity Genomics (CBG) in Guelph, Canada. All collateral data and successful barcodes are permanently and publicly deposited in the Barcode of Life Data System (BOLD, www.boldsystems.org) (Ratnasingham and Hebert 2007), and subsequently migrated to GenBank. Each barcoded specimen also receives an accession number from the Barcode of Life Data System (BOLD) and GenBank. Since the inventory is continually adding new specimens, these can be found by searching the genus *Vibrissina* in BOLD.

All inventoried specimens discussed herein were collected under Costa Rican government research permits issued to DHJ, and Tachinidae samples were exported under permit by DHJ from Costa Rica to their final depository in the CNC in Ottawa, Canada. Tachinid identifications for the inventory were done by DHJ in coordination with a) visual inspection by AJF and DMW, b) DNA barcoding by MAS and c) databasing/correlation with host caterpillars by DHJ and WH via the inventory itself.

The date of capture cited for each specimen is the date of eclosion of the fly and not the date of capture of the caterpillar. Eclosion date is much more representative of the time when that fly species is on the wing than is the time of capture of the parasitized caterpillar. The “collector” is the parataxonomist who found the caterpillar, rather than the person who later retrieved the newly eclosed fly and processed it by freezing, pinning, labelling and oven-drying. The holotypes and paratypes of the newly-described species are housed in the Diptera collection of the Canadian National Collection (CNC).

### Interim names of undescribed host species

Names of undescribed host species follow a standardized, interim naming system used for taxonomic units considered as distinct species and identified by DNA barcodes. The interim names are given in the format "*Eois* Janzen52", where the species epithet is composed of the name of the taxonomist who identified the species and a number. This prevents confusion with already described species while maintaining traceability of each undescribed species within the ACG project.

### Barcoding methods

We analyzed DNA barcodes (the 5’ region of the cytochrome c oxidase I (CO1) gene (Hebert et al. 2004)) for all specimens of ACG *Vibrissina*. Barcodes were amplified from total DNA extracts that had been prepared from single legs using a standard glass fiber protocol (Ivanova et al. 2006). This 658 bp region near the 5’ terminus of the CO1 gene was then generated using standard insect primers (LepF1–LepR1) following established protocols for production and quality control (see Smith et al. 2006, Smith et al. 2007, Smith et al. 2008).

## Taxon treatments

### Vibrissina

Rondani, 1861

Vibrissina Rondani, 1861: 35. Type species: *Frontina demissa* Meigen, 1838 [misidentified, =*Tachina turrita* Meigen, 1824], by original designation.
Microvibrissina Villeneuve, 1911: 82. Type species: *Tachina muscaria* Fallén sensu Meigen [misidentified, = *Latreillia debilitata* Pandellé, 1896: 110]. Synonymy by Herting (1984: 186). As explained by Herting (1984) the synonymy results from a misidentification of the type-species of *Microvibrissina* Villeneuve. The specimen under the name *Tachina muscaria* Fallen in Meigen's collection is actually a *Vibrissina turrita*. Villeneuve saw it, mistook the sex and misidentified the specimen as a *V. debilitata* (Herting 1984).
Spathimeigenia Townsend, 1915: 19. Type species: *Spathimeigenia spinigera* Townsend, 1915, by original designation. Synonymy proposed by Wood (1985: 86).
Hylotomomyia Townsend, 1916: 31. Type species: *Admontia hylotomae* Coquillett, 1898, by original designation. Synonymy proposed by Wood (1985: 86).
Schizocerophaga Townsend, 1916: 77. Type species: *Schizocerophaga leibyi* Townsend, 1916, by original designation. Synonymy proposed by Wood (1985: 86).
Jicaltepecia Townsend, 1917: 49. Type species: *Jicaltepecia rafaela* Townsend, 1917, by original designation. Synonymy proposed by Wood (1985: 86).
Acemeigenia Townsend, 1927: 241. Type species: *Acemeigenia inca* Townsend, 1927, by original designation. Synonymy proposed by Wood (1985: 87).
Hypophylax Townsend, 1935: 232. Type species: *Hypophylax prospheryx* Townsend, 1935, by original designation. Synonymy proposed by Wood (1985: 87).
Neoswaldia Mesnil, 1960: 655. Type species: *Hylotomomyia buckelli* Curran, 1926, by monotypy. Synonymy proposed by Wood (1985: 87).
Vibrissina **Previously described Neotropical species included in *Vibrissina***:
Vibrissina *aberrans* Wulp, 1890: 198 (*Anisia*). Lectotype male (NHMUK), by designation of Wood (1985) [examined by DMW]. Type locality: Mexico, Guerrero, Omilteme.
albopicta Bigot, 1889: 258 (*Chaetolyga*). Lectotype female (NHMUK), by designation of Wood (1985) [examined by DMW]. Type locality: Mexico.
Vibrissina *bilineata* Wulp, 1890: 112 (*Masicera*). Holotype female (NHMUK) [examined by DMW]. Type locality: Mexico, North Yucatan, Temax.
Vibrissina *candicans* Wulp, 1890: 194 (*Anisia*). Holotype male (NHMUK), published as female [examined by DMW]. Type locality: Mexico, Morelos, Cuernavaca.
Vibrissina *carinata* Wulp, 1890: 184 (*Telothyria*). Lectotype female (NHMUK), by designation of Wood (1985) [examined by DMW]. Type locality: Mexico, Veracruz, Atoyac.
Vibrissina *curva* Wulp, 1890: 177 (*Telothyria*). Holotype male (NHMUK) [examined by DMW]. Type locality: Mexico, Guerrero, Savana Grande.
Vibrissina *dieloceri* Townsend, 1942: 438 (*Hylotomomyia*). Lectotype female (MZSP), [examined by DMW] by fixation of Toma & Nihei (2006: 247) (examination of “Holótipo f.” from Minas Gerais in MZSP is regarded as a lectotype fixation). Type locality: Brazil, Minas Gerais, Para de Minas, Florestal (reared January 25 from cocoon mass of *Dielocerus formosus* (Wood, personal note)).
Vibrissina *fasciata* Wulp, 1890: 179 (*Telothyria*). Lectotype male (NHMUK), by designation of Wood (1985) [examined by DMW]. Type locality: Mexico, Tabasco, Teapa.
Vibrissina *flavocalyptrata* Brèthes, 1909: 94 (*Vibrissina*). Holotype female (MACN, or lost). Type locality: Argentina, Mendoza, Valle del Río Tupungato.
Vibrissina *forticula* Wulp, 1890: 174 (*Telothyria*). Lectotype male (NHMUK), by designation of Wood (1985) [examined by DMW]. Type locality: Mexico, Guerrero, Amula.
Vibrissina *sublineata* Wulp, 1890: 181 (*Telothyria*). Holotype female (NHMUK) [examined by DMW]. Type locality: Mexico, Guerrero, Amula.
inca Townsend, 1927: 282 (*Acemeigenia*). Holotype male (USNM) [examined by DMW]. Type locality: Peru, Cuzco.
insecta Giglio-Tos, 1893: 7 (*Degeeria*). Holotype male (MRSN), published as female [examined by DMW]. Type locality: Mexico.
Vibrissina *itaquaquecetubae* Townsend, 1929: 372 (*Jicaltepecia*). Seven syntypes: 6 males, 1 female (USNM) [examined by DMW]. Type locality: Brazil, São Paulo, Itaquaquecetuba.
Vibrissina *lepida* Wulp, 1890: 135 (*Myobia*). Lectotype male (NHMUK), by designation of Wood (1985) [examined by DMW]. Type locality: Mexico, Guerrero, Omilteme.
Vibrissina *mexicana* Aldrich, 1931: 5 (*Spathimeigenia*). Holotype male (USNM) [examined by DMW]. Type locality: Mexico, Michoacan, Erongaricaro.
Vibrissina *mucorea* Wulp, 1890: 199 (*Anisia*). Lectotype male (NHMUK), by designation of Wood (1985) [examined by DMW]. Type locality: Mexico, Guerrero, Chilpancingo.
Vibrissina *obscura* Aldrich, 1931: 6 (*Spathimeigenia*). Holotype male (USNM) [examined by DMW]. Type locality: Mexico.
prospheryx Townsend, 1935: 232 (*Hypophylax*). Holotype female (NHMUK) [examined by DMW]. Type locality: Guyana, Pacaraima Mts., Upper Ireng River.
rafaela Townsend, 1917: 49 (*Jicaltepecia*). Holotype female (USNM) [examined by DMW]. Type locality: Mexico, Veracruz, Jicaltepec, San Rafael.
Vibrissina *remota* Wulp, 1890: 181 (*Telothyria*). Lectotype female (NHMUK), by designation of Wood (1985) [examined by DMW]. Type locality: Mexico, Guerrero, Xucumanatlan.
Vibrissina *scita* Walker, 1853:302 (*Tachina*). Holotype female (NHMUK) [examined by DMW]. Type locality: Brazil.
Vibrissina *vaciva* Wulp, 1890: 176 (*Telothyria*). Holotype male (NHMUK) [examined by DMW]. Type locality: Mexico, Guerrero, Chilpancingo.
Vibrissina *vicina* Wulp, 1890: 184 (*Telothyria*). Lectotype female (NHMUK), by designation of Wood (1985) [examined by DMW]. Type locality: Mexico, Guerrero, Xucumanatlan.
Vibrissina *zonata* Bigot, 1889: 261 (*Ceromasia*). Holotype female (NHMUK) [examined by DMW]. Type locality: Mexico.
Vibrissina Frontina demissa Meigen, 1838Rondani 1861: 35. misidentified = Tachina turrita Meigen, 1824

#### Description

Male. **Head**: vertex 1/4–1/3 of head width; 1–3 reclinate orbital bristles; anteriormost reclinate orbital bristle distinctly longer than uppermost frontal bristle; ocellar bristles well developed, long, and divergent; eye bare or at most with minute, inconspicuous hairs all but invisible except under certain angles of light; parafacial on most New World species haired on lower half or more, bare in few New World species (Wood 1985); parafrontal bearing hairs interspersed among frontal bristles; lower margin of face at level of vibrissa not visible in profile; facial ridge bristled; subvibrissal ridge short, usually with 3 or fewer bristles; anterior margin of postgena concave, usually without a genal dilation; genal groove extensive, often sparsely tomentose (suggesting Dexiini); postgena bearing few widely spaced bristles, the anteriormost usually larger and distinctly isolated from the rest; arista minutely pubescent, usually distinctly thickened on basal fourth or fifth. **Thorax**: prosternum setose; proepisternum bare; postpronotum with 3 bristles in a straight line or slightly curved row; katepisternum with 2 or 3 bristles, when 3 present then two anterior to suture and one posterior; lateral scutellar bristles shorter than subapical bristles, curved medially or straight, divergent, as long as subapical bristles. Legs: fore tibia with 1 posterior bristle; mid tibia with 1 anterodorsal bristle. Wings: vein R_4+5_ setose, bearing only 2–3 bristles dorsally at base. **Abdomen**: mid-dorsal depression on syntergite 1+2 (ST1+2) extending almost to hind margin; two median marginal bristles on T3 and often also on ST1+2; row of marginal bristles on T4 and T5; 1–3 pairs of median discal bristles on T3–T5 (5th tergite sometimes bearing a complete row of discal bristles). Female as male except: 2 pairs of proclinate orbital bristles present; T3 and T4 ventolaterally flattened, their edges either studded with spines or stout bristles.

#### Diagnosis

Our observations of New World species of *Vibrissina* confirm those made by Wood (1985). One of the main characteristics of the genus in the New World is that all species possess hairs on the lower half of the parafacial, usually separated from the lowest frontal bristle by a bare gap, a character trait which can be used to distinguish them from the rest of the Blondeliini, with the exception of *Lixophaga retiniae* (Coquillett) and *Erynniopsis antennata* (Rondani) (both restricted to California and Oregon), and all species of *Cryptomeigenia* Brauer & Bergenstamm, which also have hairs on the lower half of the parafacial, having 4 or more bristles on the subvibrissal ridge. Wood (1985) also cites *Enrogalia morigera* Reinhard and *Istocheta aldrichi* (Mesnil) as having hairs on the parafacial; however, in the case of both these species the hairs are most abundant on the upper half, decreasing in size and density toward the lower half. Old World species of *Vibrissina*, which lack parafacial hairs, can be separated only with difficulty from species of *Eucelatoria* Townsend, and can be separated only by the lack of a genal dilation.

#### Distribution

Widespread throughout the Palearctic, and from Mexico to Brazil in the Neotropical Region (Wood 1985).

#### Ecology

Almost all members of *Vibrissina* are parasitic on the caterpillar-like larvae of sawflies in the families Argidae, Diprionidae, and Tenthredinidae (Wood 1985). The present data from ACG inventoried larvae support this.

### Vibrissina albopicta

(Bigot, 1889)

Chaetolyga albopicta Bigot, 1889: 258. Lectotype female (NHMUK), by designation of Wood (1985) [examined by DMW]. Type locality: Mexico.

#### Materials

- **Type status:** Other material. **Occurrence:** occurrenceDetails: http://www.boldsystems.org/index.php/API_Public/specimen?ids=ASHYM1999-13; catalogNumber: DHJPAR0052645; recordNumber: 13-SRNP-18822; recordedBy: Guillermo Pereira; individualID: DHJPAR0052645; individualCount: 1; sex: male; lifeStage: adult; **Taxon:** scientificName: Vibrissina albopicta; nameAccordingTo: (Bigot, 1889); phylum: Arthropoda; class: Insecta; order: Diptera; family: Tachinidae; genus: Vibrissina; specificEpithet: albopicta; scientificNameAuthorship: (Bigot, 1889); **Location:** continent: Central America; country: Costa Rica; stateProvince: Guanacaste; county: Sector Santa Rosa; locality: Area de Conservación Guanacaste; verbatimLocality: Area Administrativa; verbatimElevation: 295; verbatimLatitude: 10.83764; verbatimLongitude: -85.61871; verbatimCoordinateSystem: decimal; decimalLatitude: 10.8376; decimalLongitude: -85.6187; **Identification:** identifiedBy: A.J. Fleming; **Event:** samplingProtocol: Reared from the larva of Durgoa mattogrossensis; verbatimEventDate: 10-Aug-2013; **Record Level:** institutionCode: CNC
- **Type status:** Other material. **Occurrence:** recordedBy: H.J. Reinhard; sex: male; lifeStage: adult; **Taxon:** scientificName: Vibrissina albopicta; nameAccordingTo: (Bigot, 1889); family: Tachinidae; genus: Vibrissina; specificEpithet: albopicta; **Location:** locationID: Santa Engracia; higherGeographyID: Tamaulipas; higherGeography: Mexico; continent: North America; country: Mexico; countryCode: MX; stateProvince: Tamaulipas; locality: Santa Engracia; verbatimLocality: Santa Engracia, Tamaulipas, Mex; **Identification:** identifiedBy: D.M. Wood; dateIdentified: 1979; **Event:** eventDate: 1937-10-20; year: 1937; month: 10; day: 20; verbatimEventDate: X-20-37; **Record Level:** institutionCode: CNC
- **Type status:** Other material. **Occurrence:** recordedBy: B.V. Peterson; sex: female; **Taxon:** scientificName: Vibrissina albopicta; nameAccordingTo: (Bigot, 1889); family: Tachinidae; genus: Vibrissina; specificEpithet: albopicta; **Location:** locationID: Sontecomapan; higherGeographyID: Veracruz; higherGeography: Mexico; continent: North America; country: Mexico; countryCode: MX; stateProvince: Veracruz; locality: Sontecomapan; verbatimLocality: MEXICO, Veracruz, Sontecomapan; **Event:** eventDate: 1969-6-20; year: 1937; month: 6; day: 20; verbatimEventDate: 20.VI.1969; **Record Level:** institutionCode: CNC
- **Type status:** Other material. **Occurrence:** recordedBy: J.G. Chillcott; sex: female; **Taxon:** scientificName: Vibrissina albopicta; nameAccordingTo: (Bigot, 1889); family: Tachinidae; genus: Vibrissina; specificEpithet: albopicta; **Location:** locationID: 12 Mi. East of Cuernavaca; higherGeographyID: Morelos; higherGeography: Mexico; continent: North America; country: Mexico; countryCode: MX; stateProvince: Morelos; locality: 12. Mi. E. of Cuernavaca; verbatimLocality: Cuernavaca, 12 Mi. E. 4300' Morelos, Mex.; verbatimElevation: 4300'; **Event:** eventDate: 14-VIII-1954; year: 1954; month: 8; day: 14; verbatimEventDate: 14-VIII-1954; **Record Level:** institutionCode: CNC
- **Type status:** Other material. **Occurrence:** recordedBy: J.G. Chillcott; sex: female; **Taxon:** scientificName: Vibrissina albopicta; nameAccordingTo: (Bigot, 1889); family: Tachinidae; genus: Vibrissina; specificEpithet: albopicta; **Location:** locationID: Xilitla; higherGeographyID: San Luis Potosí; higherGeography: Mexico; continent: North America; country: Mexico; countryCode: MX; stateProvince: San Luis Potosí; locality: Xilitla; verbatimLocality: Xilitla, 1800' S.L.P., Mexico; verbatimElevation: 1800'; **Event:** eventDate: 1954-VII-24; year: 1954; month: 7; day: 24; verbatimEventDate: 24-VII-1954; **Record Level:** institutionCode: CNC
- **Type status:** Other material. **Occurrence:** recordedBy: R. & K. Dreisbach; sex: female; **Taxon:** scientificName: Vibrissina albopicta; nameAccordingTo: (Bigot, 1889); family: Tachinidae; genus: Vibrissina; specificEpithet: albopicta; **Location:** locationID: Hujintlan; higherGeographyID: Morelos; higherGeography: Mexico; continent: North America; country: Mexico; countryCode: MX; stateProvince: Morelos; locality: Hujintlan; verbatimLocality: Hujintlan, Morelos, Mexico; **Event:** eventDate: 1956-VIII-22; year: 1956; month: 8; day: 22; verbatimEventDate: 8-22-56; **Record Level:** institutionCode: CNC

#### Description

**Male** (Fig. 2). Length: 6–8mm. **Head** (Fig. 2b): parafrontal and parafacial silver tomentose; postorbit with slight golden-yellow tinge but overall silver in appearance; face, gena, and postgena silver tomentose; antenna black-brown; arista reddish brown; gena 0.14X eye height; 3 reclinate orbital bristles; frontal bristles not extending beyond lower level of pedicel; first flagellomere short of facial margin by 2X length of pedicel. **Thorax** (Fig. 2a, c): dorsum gray tomentose with 4 dorsal vittae barely visible presuturally, postsuturally all blurred together into a black smudge with a dark overall appearance (this trait only visible under natural light, not pictured); thorax gold tinged on posterior edge adjacent to scutellum; scutellum silver tomentose over black ground color; katepisternum, anepisternum and anepimeron silver-gray tomentose; 3 strong katepisternal bristles; 2 pairs of short discal scutellar bristles; apical scutellars weak and crossed. Legs: tibiae black overall, with light silver tomentosity visible under certain angles of light. Wings: smoky gray, bearing 2–3 short setulae dorsally at base of vein R_4+5_. **Abdomen** (Fig. 2a): ground color of abdomen dark brown-black laterally, black dorsally; ST1+2 all black, with mid-dorsal depression extending halfway to margin of syntergite; anterior margins of T3 and T4 bearing gray tomentum covering ⅓ of tergal surface; gray tomentum absent from T5; ST1+2 and T3 with 1 pair of median marginal bristles; T3 and T4 each with 1 pair of underdeveloped median discal bristles; T4 and T5 each bearing 1 row of marginal bristles; no discal bristles on T5. **Male terminalia**: not dissected.

**Female** (not pictured, due to lack of photographic quality specimens). Length: 5–6mm. As male, with the exception of the following characters: **thorax**: dorsum and scutellum entirely silver tomentose; thoracic vittae distinct both pre- and postsuturally. **Abdomen**: entirely black in ground color, ventrolaterally flattened; all tergites lacking discal bristles; median marginal bristles present on ST1+2 and T3; T4 and T5 each with a row of marginal bristles; mid-ventral portion of T3–T5 tergites with a row of strong, stout spines.

#### Diagnosis

*Vibrissina albopicta* can be differentiated from its congeners by the following combination of traits: parafacial and parafrontal silver; thoracic tomentum silver-gray on both dorsal and lateral surfaces; abdominal ground color almost entirely black with only traces of dark brown; silver tomentum extending to over 30% of T3 and T4, but absent on T5; ST1+2 and T3 with only one pair of median marginals, T4 and T5 with a complete row of marginals; discals on T3 and T4 underdeveloped in male, absent in female.

#### Distribution

Mexico: ranging from San Luis Potosí, Southeast to Morelos; Costa Rica: ACG, Guanacaste Province, 295 m.

#### Ecology

**Hosts**: in ACG, *V. albopicta* has been reared once from the larvae of the sawfly *Durgoa mattogrossensis* Malaise (Argidae), feeding on the leaves of *Bauhinia ungulata* L. (Fabaceae).

### Vibrissina danmartini

Fleming & Wood sp. n.

urn:lsid:zoobank.org:act:B6B542B7-A072-44D2-8B0B-CEFCAFBB0780

#### Materials

- **Type status:** Holotype. **Occurrence:** occurrenceDetails: http://janzen.sas.upenn.edu; catalogNumber: DHJPAR0017886; recordedBy: D.H. Janzen, W. Hallwachs & Gusaneros; individualID: DHJPAR0017886; individualCount: 1; sex: male; lifeStage: adult; preparations: pinned; otherCatalogNumbers: ASTAR596-07, DHJPAR0017886, 99-SRNP-9357, BOLD:ABY9311; **Taxon:** scientificName: Vibrissina danmartini; phylum: Arthropoda; class: Insecta; order: Diptera; family: Tachinidae; genus: Vibrissina; specificEpithet: danmartini; scientificNameAuthorship: Fleming & Wood, 2016; **Location:** continent: Central America; country: Costa Rica; countryCode: CR; stateProvince: Guanacaste; county: Sector Santa Rosa; locality: Area de Conservación Guanacaste; verbatimLocality: Area Administrativa; verbatimElevation: 295; verbatimLatitude: 10.8376; verbatimLongitude: -85.6187; verbatimCoordinateSystem: Decimal; decimalLatitude: 10.8376; decimalLongitude: -85.6187; **Identification:** identifiedBy: AJ Fleming; dateIdentified: 2016; **Event:** samplingProtocol: Reared from the larvae of the Argid sawfly, Durgoa mattogrossensis; verbatimEventDate: 07-Oct-1999; **Record Level:** language: en; institutionCode: CNC; collectionCode: Insects; basisOfRecord: Pinned Specimen
- **Type status:** Paratype. **Occurrence:** occurrenceDetails: http://janzen.sas.upenn.edu; catalogNumber: DHJPAR0017892; recordedBy: D.H. Janzen, W. Hallwachs & Gusaneros; individualID: DHJPAR0017892; individualCount: 1; sex: female; lifeStage: adult; preparations: pinned; otherCatalogNumbers: ASTAR602-07, DHJPAR0017892, 99-SRNP-9255, BOLD:ABY9311; **Taxon:** scientificName: Vibrissina danmartini; phylum: Arthropoda; class: Insecta; order: Diptera; family: Tachinidae; genus: Vibrissina; specificEpithet: danmartini; scientificNameAuthorship: Fleming & Wood, 2016; **Location:** continent: Central America; country: Costa Rica; countryCode: CR; stateProvince: Guanacaste; county: Sector Santa Rosa; locality: Area de Conservación Guanacaste; verbatimLocality: Area Administrativa; verbatimElevation: 295; verbatimLatitude: 10.8376; verbatimLongitude: -85.6187; verbatimCoordinateSystem: Decimal; decimalLatitude: 10.8376; decimalLongitude: -85.6187; **Identification:** identifiedBy: AJ Fleming; dateIdentified: 2016; **Event:** samplingProtocol: Reared from the larvae of the Argid sawfly, Durgoa mattogrossensis; verbatimEventDate: 07-Nov-1999; **Record Level:** language: en; institutionCode: CNC; collectionCode: Insects; basisOfRecord: Pinned Specimen
- **Type status:** Paratype. **Occurrence:** occurrenceDetails: http://janzen.sas.upenn.edu; catalogNumber: DHJPAR0017885; recordedBy: D.H. Janzen, W. Hallwachs & Gusaneros; individualID: DHJPAR0017885; individualCount: 1; sex: male; lifeStage: adult; preparations: pinned; otherCatalogNumbers: ASTAR595-07, DHJPAR0017885, 99-SRNP-10233, BOLD:ABY9311; **Taxon:** scientificName: Vibrissina danmartini; phylum: Arthropoda; class: Insecta; order: Diptera; family: Tachinidae; genus: Vibrissina; specificEpithet: danmartini; scientificNameAuthorship: Fleming & Wood, 2016; **Location:** continent: Central America; country: Costa Rica; countryCode: CR; stateProvince: Guanacaste; county: Sector Santa Rosa; locality: Area de Conservación Guanacaste; verbatimLocality: Parcela John Sullivan; verbatimElevation: 280; verbatimLatitude: 10.8734; verbatimLongitude: -85.6265; verbatimCoordinateSystem: Decimal; decimalLatitude: 10.8734; decimalLongitude: -85.6265; **Identification:** identifiedBy: AJ Fleming; dateIdentified: 2016; **Event:** samplingProtocol: Reared from the larvae of the Argid sawfly, Durgoa mattogrossensis; verbatimEventDate: 07-Oct-1999; **Record Level:** language: en; institutionCode: CNC; collectionCode: Insects; basisOfRecord: Pinned Specimen
- **Type status:** Paratype. **Occurrence:** occurrenceDetails: http://janzen.sas.upenn.edu; catalogNumber: DHJPAR0017887; recordedBy: D.H. Janzen, W. Hallwachs & Gusaneros; individualID: DHJPAR0017887; individualCount: 1; sex: male; lifeStage: adult; preparations: pinned; otherCatalogNumbers: ASTAR597-07, DHJPAR0017887, 99-SRNP-10150, BOLD:ABY9311; **Taxon:** scientificName: Vibrissina danmartini; phylum: Arthropoda; class: Insecta; order: Diptera; family: Tachinidae; genus: Vibrissina; specificEpithet: danmartini; scientificNameAuthorship: Fleming & Wood, 2016; **Location:** continent: Central America; country: Costa Rica; countryCode: CR; stateProvince: Guanacaste; county: Sector Santa Rosa; locality: Area de Conservación Guanacaste; verbatimLocality: Parcela John Sullivan; verbatimElevation: 280; verbatimLatitude: 10.8734; verbatimLongitude: -85.6265; verbatimCoordinateSystem: Decimal; decimalLatitude: 10.8734; decimalLongitude: -85.6265; **Identification:** identifiedBy: AJ Fleming; dateIdentified: 2016; **Event:** samplingProtocol: Reared from the larvae of the Argid sawfly, Durgoa mattogrossensis; verbatimEventDate: 07-Oct-1999; **Record Level:** language: en; institutionCode: CNC; collectionCode: Insects; basisOfRecord: Pinned Specimen
- **Type status:** Paratype. **Occurrence:** occurrenceDetails: http://janzen.sas.upenn.edu; catalogNumber: DHJPAR0017890; recordedBy: D.H. Janzen, W. Hallwachs & Gusaneros; individualID: DHJPAR0017890; individualCount: 1; sex: female; lifeStage: adult; preparations: pinned; otherCatalogNumbers: ASTAR600-07, DHJPAR0017890, 99-SRNP-10015, BOLD:ABY9311; **Taxon:** scientificName: Vibrissina danmartini; phylum: Arthropoda; class: Insecta; order: Diptera; family: Tachinidae; genus: Vibrissina; specificEpithet: danmartini; scientificNameAuthorship: Fleming & Wood, 2016; **Location:** continent: Central America; country: Costa Rica; countryCode: CR; stateProvince: Guanacaste; county: Sector Santa Rosa; locality: Area de Conservación Guanacaste; verbatimLocality: Parcela John Sullivan; verbatimElevation: 280; verbatimLatitude: 10.8734; verbatimLongitude: -85.6265; verbatimCoordinateSystem: Decimal; decimalLatitude: 10.8734; decimalLongitude: -85.6265; **Identification:** identifiedBy: AJ Fleming; dateIdentified: 2016; **Event:** samplingProtocol: Reared from the larvae of the Argid sawfly, Durgoa mattogrossensis; verbatimEventDate: 07-Oct-1999; **Record Level:** language: en; institutionCode: CNC; collectionCode: Insects; basisOfRecord: Pinned Specimen
- **Type status:** Paratype. **Occurrence:** occurrenceDetails: http://janzen.sas.upenn.edu; catalogNumber: DHJPAR0017891; recordedBy: D.H. Janzen, W. Hallwachs & Gusaneros; individualID: DHJPAR0017891; individualCount: 1; sex: female; lifeStage: adult; preparations: pinned; otherCatalogNumbers: ASTAR601-07, DHJPAR0017891, 99-SRNP-9302, BOLD:ABY9311; **Taxon:** scientificName: Vibrissina danmartini; phylum: Arthropoda; class: Insecta; order: Diptera; family: Tachinidae; genus: Vibrissina; specificEpithet: danmartini; scientificNameAuthorship: Fleming & Wood, 2016; **Location:** continent: Central America; country: Costa Rica; countryCode: CR; stateProvince: Guanacaste; county: Sector Santa Rosa; locality: Area de Conservación Guanacaste; verbatimLocality: Area Administrativa; verbatimElevation: 295; verbatimLatitude: 10.8376; verbatimLongitude: -85.6187; verbatimCoordinateSystem: Decimal; decimalLatitude: 10.8376; decimalLongitude: -85.6187; **Identification:** identifiedBy: AJ Fleming; dateIdentified: 2016; **Event:** samplingProtocol: Reared from the larvae of the Argid sawfly, Durgoa mattogrossensis; verbatimEventDate: 07-Oct-1999; **Record Level:** language: en; institutionCode: CNC; collectionCode: Insects; basisOfRecord: Pinned Specimen
- **Type status:** Paratype. **Occurrence:** occurrenceDetails: http://janzen.sas.upenn.edu; catalogNumber: DHJPAR0017889; recordedBy: D.H. Janzen, W. Hallwachs & Gusaneros; individualID: DHJPAR0017889; individualCount: 1; sex: female; lifeStage: adult; preparations: pinned; otherCatalogNumbers: ASTAR599-07, DHJPAR0017889, 99-SRNP-9127, BOLD:ABY9311; **Taxon:** scientificName: Vibrissina danmartini; phylum: Arthropoda; class: Insecta; order: Diptera; family: Tachinidae; genus: Vibrissina; specificEpithet: danmartini; scientificNameAuthorship: Fleming & Wood, 2016; **Location:** continent: Central America; country: Costa Rica; countryCode: CR; stateProvince: Guanacaste; county: Sector Santa Rosa; locality: Area de Conservación Guanacaste; verbatimLocality: Area Administrativa; verbatimElevation: 295; verbatimLatitude: 10.8376; verbatimLongitude: -85.6187; verbatimCoordinateSystem: Decimal; decimalLatitude: 10.8376; decimalLongitude: -85.6187; **Identification:** identifiedBy: AJ Fleming; dateIdentified: 2016; **Event:** samplingProtocol: Reared from the larvae of the Argid sawfly, Durgoa mattogrossensis; verbatimEventDate: 07-Oct-1999; **Record Level:** language: en; institutionCode: CNC; collectionCode: Insects; basisOfRecord: Pinned Specimen
- **Type status:** Paratype. **Occurrence:** occurrenceDetails: http://janzen.sas.upenn.edu; catalogNumber: DHJPAR0017888; recordedBy: D.H. Janzen, W. Hallwachs & Gusaneros; individualID: DHJPAR0017888; individualCount: 1; sex: female; lifeStage: adult; preparations: pinned; otherCatalogNumbers: ASTAR598-07, DHJPAR0017888, 99-SRNP-9160; **Taxon:** scientificName: Vibrissina danmartini; phylum: Arthropoda; class: Insecta; order: Diptera; family: Tachinidae; genus: Vibrissina; specificEpithet: danmartini; scientificNameAuthorship: Fleming & Wood, 2016; **Location:** continent: Central America; country: Costa Rica; countryCode: CR; stateProvince: Guanacaste; county: Sector Santa Rosa; locality: Area de Conservación Guanacaste; verbatimLocality: Area Administrativa; verbatimElevation: 295; verbatimLatitude: 10.8376; verbatimLongitude: -85.6187; verbatimCoordinateSystem: Decimal; decimalLatitude: 10.8376; decimalLongitude: -85.6187; **Identification:** identifiedBy: AJ Fleming; dateIdentified: 2016; **Event:** samplingProtocol: Reared from the larvae of the Argid sawfly, Durgoa mattogrossensis; verbatimEventDate: 07-Oct-1999; **Record Level:** language: en; institutionCode: CNC; collectionCode: Insects; basisOfRecord: Pinned Specimen
- **Type status:** Paratype. **Occurrence:** occurrenceDetails: http://janzen.sas.upenn.edu; catalogNumber: DHJPAR0017874; recordedBy: D.H. Janzen, W. Hallwachs & Gusaneros; individualID: DHJPAR0017874; individualCount: 1; sex: male; lifeStage: adult; preparations: pinned; otherCatalogNumbers: ASTAR584-07, DHJPAR0017874, 99-SRNP-9604, BOLD:ABY9311; **Taxon:** scientificName: Vibrissina danmartini; phylum: Arthropoda; class: Insecta; order: Diptera; family: Tachinidae; genus: Vibrissina; specificEpithet: danmartini; scientificNameAuthorship: Fleming & Wood, 2016; **Location:** continent: Central America; country: Costa Rica; countryCode: CR; stateProvince: Guanacaste; county: Sector Santa Rosa; locality: Area de Conservación Guanacaste; verbatimLocality: Area Administrativa; verbatimElevation: 295; verbatimLatitude: 10.8376; verbatimLongitude: -85.6187; verbatimCoordinateSystem: Decimal; decimalLatitude: 10.8376; decimalLongitude: -85.6187; **Identification:** identifiedBy: AJ Fleming; dateIdentified: 2016; **Event:** samplingProtocol: Reared from the larvae of the Argid sawfly, Durgoa mattogrossensis; verbatimEventDate: 15-Jul-1999; **Record Level:** language: en; institutionCode: CNC; collectionCode: Insects; basisOfRecord: Pinned Specimen

#### Description

**Male** (Fig. 3a, b, c). Length: 6–7mm. **Head** (Fig. 3b): parafrontal, parafacial and postorbit golden yellow tomentose; face, gena, and postgena silver tomentose; antenna black-brown; arista reddish brown; gena 0.2X eye height; 3 reclinate orbital bristles; frontal bristles not extending beyond lower level of pedicel; first flagellomere short of facial margin by length of pedicel. **Thorax** (Fig. 3a, c): dorsum gold tomentose with 4 distinct vittae visible presuturally, postsuturally all blurred together into a black smudge, giving the thorax a very dark overall appearance; scutellum glabrous black except at apex, where it bears a light gold tinge; anepisternum and anepimeron gold tomentose with silver margins (anepimeron only very slightly gold); katepisternum gold along dorsal edge but overall silver-gray tomentose; 3 strong katepisternal bristles; 2 short pairs of discal scutellar bristles; apical scutellars weak and strongly divergent. Legs: dark brown, tibiae with a light brown-reddish tinge under certain angles of light. Wings: smoky gray, bearing 2–3 short setulae dorsally at the base of R_4+5_. **Abdomen** (Fig. 3a): ground color of abdomen light brown laterally, dark brown-black dorsally; ST1+2 all black, mid-dorsal depression extending halfway to margin of syntergite; anterior margin of T3, T4 and T5 bearing gold tomentum over less than 1/3 of tergal surface; ST1+2 and T3 each with 1 pair of median marginal bristles; T3 and T4 each with 3 pairs of median discal bristles; T4 and T5 each bearing 1 complete row of marginal bristles; T5 bearing 2 rows of discal bristles. **Terminalia** (Fig. 4): sternite 5 with two prominent rounded lobes, flanking a wide U-shaped median cleft; posterior lobes 0.61X length of anterior plate; inner margin covered in dense tomentum appearing darker than surrounding cuticle; posterior lobes bearing short stout bristles throughout; anterior plate bare, wider than posterior lobes; cerci subtriangular in dorsal view, 1.38X as long as wide, separated along entire length; in lateral view cerci dorsally straight, apically pointed and with a rounded ventral edge giving it a knife-like appearance; surstylus, in lateral view, weakly constricted at midpoint, giving it a leaf-like appearance; postgonite parallel-sided and rounded at tip when viewed laterally, entire structure bent midway at a rounded 90 degree angle, giving it a rounded L-shaped appearance, with a small, apically flattened anterobasal heel bearing few minute cilia; ventral sclerite of distiphallus elongate and linear; membranous acrophallus terminating in a small downward-curved apical hook, visible laterally.

**Female** (Fig. 3d, e, f). Length: 5–6mm. As male, with the exception of the following characters: **thorax**: dorsum of thorax and scutellum almost entirely gold tinged with no scutellar discals; thoracic vittae distinct both pre- and postsuturally; ventral katepisternal bristle very reduced compared to the other two. **Abdomen**: black ground color over its entirety; brassy tomentum along anterior 1/2 of tergites T3, T4, and T5; ventrolaterally flattened; no visible discal bristles on any segment; 1 pair of reduced marginal bristles on ST1+2 and T3; T4 and T5 each with 1 row of marginal bristles; mid-ventral portion of T3–T5 with a row of strong stout spines.

#### Diagnosis

*Vibrissina danmartini* **sp. n.** can be differentiated from its congeners by the combination of the following traits: parafacial and parafrontal gold with parafacial haired; outer thoracic vittae reduced presuturally, not extending to suture, postsuturally also weak; ST1+2, T3, and 50% of T4 bearing orange ground color lateroventrally; abdominal tomentum extending over 60% of tergites T3, T4, and T5, with tomentum broken up by a dorsocentral dark stripe; in females, abdominal tomentum brassy-gold; marginal bristles reduced on ST1+2 and T3, rows of marginal bristles on T4 and T5.

#### Etymology

*Vibrissina danmartini* **sp. n.** is dedicated to Mr. Dan Martin, now of Washington, D.C., formerly of Chicago, Illinois, in recognition of his administrative and philanthropic support of the biodiversity development concept that gave birth in 1989 to the INBio national biodiversity inventory collections, now part of Museo Nacional de Costa Rica, and his decades of steadfast support for the protection of tropical biodiversity in general, and specifically that of Costa Rica.

#### Distribution

Costa Rica, ACG, Prov. Guanacaste, dry forest, between 280–295m.

#### Ecology

**Hosts**: reared 37 times from larvae of the sawfly *Durgoa mattogrossensis* Malaise (Argidae), which feed on the leaves of *Bauhinia ungulata* L. (Fabaceae).

### Vibrissina hallwachsorum

Fleming & Wood sp. n.

urn:lsid:zoobank.org:act:674B0A7A-528F-4CC2-9421-E908CB20CB7C

#### Materials

- **Type status:** Holotype. **Occurrence:** occurrenceDetails: http://janzen.sas.upenn.edu; catalogNumber: DHJPAR0017884; recordedBy: D.H. Janzen, W. Hallwachs & Gusaneros; individualID: DHJPAR0017884; individualCount: 1; sex: female; lifeStage: adult; preparations: pinned; otherCatalogNumbers: ASTAR594-07, 97-SRNP-1013.03, BOLD:ABY9310; **Taxon:** scientificName: Vibrissina hallwachsorum; phylum: Arthropoda; class: Insecta; order: Diptera; family: Tachinidae; genus: Vibrissina; specificEpithet: hallwachsorum; scientificNameAuthorship: Fleming & Wood, 2016; **Location:** continent: Central America; country: Costa Rica; countryCode: CR; stateProvince: Guanacaste; county: Sector Cacao; locality: Area de Conservación Guanacaste; verbatimLocality: Sendero Nayo; verbatimElevation: 1090; verbatimLatitude: 10.9245; verbatimLongitude: -85.4695; verbatimCoordinateSystem: Decimal; decimalLatitude: 10.9245; decimalLongitude: -85.4695; **Identification:** identifiedBy: AJ Fleming; dateIdentified: 2016; **Event:** samplingProtocol: Reared from the larvae of the Tenthredinid sawfly, Waldheimia interstitialis; verbatimEventDate: 05-Jan-1997; **Record Level:** language: en; institutionCode: CNC; collectionCode: Insects; basisOfRecord: Pinned Specimen

#### Description

**Male.** Unknown. **Female** (Fig. 5). Length: 5mm. **Head** (Fig. 5b): parafrontal, postorbit, parafacial, face, gena, and postgena gold tomentose; antenna black-brown; arista reddish brown; gena 0.25X eye height; 2 pairs of proclinate orbital bristles; frontal bristles not reaching below lower margin of pedicel; first flagellomere short of facial margin by 2X length of pedicel. **Thorax** (Fig. 5a, c): dorsum gold tomentose with 4 distinct dorsal vittae visible presuturally, postsuturally vittae smudging together, covering slightly over ½ of postsutural scutum; scutellum gold tomentose over posterior 1/2, up to insertion of scutellar discals; 1 pair of discal scutellar bristles; apical scutellars weak and convergent; 3 strong katepisternal bristles, with ventral katepisternal bristle appearing greatly reduced compared to other 2, but still well developed; anepisternum, anepimeron and katepisternum slightly gold tomentose. Legs: reddish brown on all segments. Wings: smoky gray, bearing 2 short setulae dorsally at the base of R4+5. **Abdomen** (Fig. 5a): ground color of abdomen dark brown-black overall; ST1+2 all black; mid-dorsal depression extending to margin of syntergite; anterior margin of T3, T4 and T5 bearing gold tomentum over more than ½ of tergal surface; ST1+2 bearing 1 pair of median marginal bristles; T4 and T5 each bearing 1 complete row of marginal bristles; T3 with 1 pair of median discal bristles; T3, T4, and T5 ventrolaterally flattened; mid-ventral portion of T3–T5 abdominal tergites with a row of strong stout spines.

#### Diagnosis

*Vibrissina hallwachsorum* **sp. n.** can be differentiated from its congeners by the combination of the following traits: parafacial and parafrontal gold; tergite 5 bearing all black ground color with silver tomentum over its entirety; and only 1 pair of discal bristles on T3.

#### Etymology

*Vibrissina hallwachsorum* **sp. n.** is dedicated to Robert and Marianne Hallwachs of Philadelphia, Pennsylvania in recognition of their seminal support in acquiring the buildings in which the INBio national biodiversity inventory collections have grown and thrived since 1989, and which are now donated to the Museo Nacional de Costa Rica.

#### Distribution

Costa Rica, ACG, Prov. Guanacaste, cloud forest, at 1090m.

#### Ecology

**Hosts**: reared once from a larva of the sawfly *Waldheimia interstitialis* (Cameron) (Tenthredinidae) feeding on the leaves of *Hamelia patens* Jacq. (Rubiaceae).

### Vibrissina randycurtisi

Fleming & Wood sp. n.

urn:lsid:zoobank.org:act:C9C5BAD2-1DB9-4DFB-8C37-8633FFBB935F

#### Materials

- **Type status:** Holotype. **Occurrence:** occurrenceDetails: http://janzen.sas.upenn.edu; catalogNumber: DHJPAR0007167; recordedBy: D.H. Janzen, W. Hallwachs & Roster Moraga; individualID: DHJPAR0007167; individualCount: 1; sex: male; lifeStage: adult; preparations: pinned; otherCatalogNumbers: ASTAV409-06, 06-SRNP-20395, BOLD:AAD6317; **Taxon:** scientificName: Vibrissina randycurtisi; phylum: Arthropoda; class: Insecta; order: Diptera; family: Tachinidae; genus: Vibrissina; specificEpithet: randycurtisi; scientificNameAuthorship: Fleming & Wood, 2016; **Location:** continent: Central America; country: Costa Rica; countryCode: CR; stateProvince: Guanacaste; county: Sector Del Oro; locality: Area de Conservación Guanacaste; verbatimLocality: Quebrada Raiz; verbatimElevation: 280; verbatimLatitude: 11.0287; verbatimLongitude: -85.4867; verbatimCoordinateSystem: Decimal; decimalLatitude: 11.0287; decimalLongitude: -85.4867; **Identification:** identifiedBy: AJ Fleming; dateIdentified: 2016; **Event:** samplingProtocol: Reared from the larvae of the Argid sawfly, Sericoceros gibbus; verbatimEventDate: 18-Feb-2006; **Record Level:** language: en; institutionCode: CNC; collectionCode: Insects; basisOfRecord: Pinned Specimen
- **Type status:** Paratype. **Occurrence:** occurrenceDetails: http://janzen.sas.upenn.edu; catalogNumber: DHJPAR0007162; recordedBy: D.H. Janzen, W. Hallwachs & Roster Moraga; individualID: DHJPAR0007162; individualCount: 1; sex: female; lifeStage: adult; preparations: pinned; otherCatalogNumbers: ASTAV404-06, 06-SRNP-20394, BOLD:AAD6317; **Taxon:** scientificName: Vibrissina randycurtisi; phylum: Arthropoda; class: Insecta; order: Diptera; family: Tachinidae; genus: Vibrissina; specificEpithet: randycurtisi; scientificNameAuthorship: Fleming & Wood, 2016; **Location:** continent: Central America; country: Costa Rica; countryCode: CR; stateProvince: Guanacaste; county: Sector Del Oro; locality: Area de Conservación Guanacaste; verbatimLocality: Quebrada Raiz; verbatimElevation: 280; verbatimLatitude: 11.0287; verbatimLongitude: -85.4867; verbatimCoordinateSystem: Decimal; decimalLatitude: 11.0287; decimalLongitude: -85.4867; **Identification:** identifiedBy: AJ Fleming; dateIdentified: 2016; **Event:** samplingProtocol: Reared from the larvae of the Argid sawfly, Sericoceros gibbus; verbatimEventDate: 10-Feb-2006; **Record Level:** language: en; institutionCode: CNC; collectionCode: Insects; basisOfRecord: Pinned Specimen
- **Type status:** Paratype. **Occurrence:** occurrenceDetails: http://janzen.sas.upenn.edu; catalogNumber: DHJPAR0007164; recordedBy: D.H. Janzen, W. Hallwachs & Roster Moraga; individualID: DHJPAR0007164; individualCount: 1; sex: female; lifeStage: adult; preparations: pinned; otherCatalogNumbers: ASTAV406-06, 06-SRNP-20393, BOLD:AAD6317; **Taxon:** scientificName: Vibrissina randycurtisi; phylum: Arthropoda; class: Insecta; order: Diptera; family: Tachinidae; genus: Vibrissina; specificEpithet: randycurtisi; scientificNameAuthorship: Fleming & Wood, 2016; **Location:** continent: Central America; country: Costa Rica; countryCode: CR; stateProvince: Guanacaste; county: Sector Del Oro; locality: Area de Conservación Guanacaste; verbatimLocality: Quebrada Raiz; verbatimElevation: 280; verbatimLatitude: 11.0287; verbatimLongitude: -85.4867; verbatimCoordinateSystem: Decimal; decimalLatitude: 11.0287; decimalLongitude: -85.4867; **Identification:** identifiedBy: AJ Fleming; dateIdentified: 2016; **Event:** samplingProtocol: Reared from the larvae of the Argid sawfly, Sericoceros gibbus; verbatimEventDate: 15-Feb-2006; **Record Level:** language: en; institutionCode: CNC; collectionCode: Insects; basisOfRecord: Pinned Specimen
- **Type status:** Paratype. **Occurrence:** occurrenceDetails: http://janzen.sas.upenn.edu; catalogNumber: DHJPAR0007168; recordedBy: D.H. Janzen, W. Hallwachs & Roster Moraga; individualID: DHJPAR0007168; individualCount: 1; sex: female; lifeStage: adult; preparations: pinned; otherCatalogNumbers: ASTAV410-06, 06-SRNP-20396, BOLD:AAD6317; **Taxon:** scientificName: Vibrissina randycurtisi; phylum: Arthropoda; class: Insecta; order: Diptera; family: Tachinidae; genus: Vibrissina; specificEpithet: randycurtisi; scientificNameAuthorship: Fleming & Wood, 2016; **Location:** continent: Central America; country: Costa Rica; countryCode: CR; stateProvince: Guanacaste; county: Sector Del Oro; locality: Area de Conservación Guanacaste; verbatimLocality: Quebrada Raiz; verbatimElevation: 280; verbatimLatitude: 11.0287; verbatimLongitude: -85.4867; verbatimCoordinateSystem: Decimal; decimalLatitude: 11.0287; decimalLongitude: -85.4867; **Identification:** identifiedBy: AJ Fleming; dateIdentified: 2016; **Event:** samplingProtocol: Reared from the larvae of the Argid sawfly, Sericoceros gibbus; verbatimEventDate: 18-Feb-2006; **Record Level:** language: en; institutionCode: CNC; collectionCode: Insects; basisOfRecord: Pinned Specimen
- **Type status:** Paratype. **Occurrence:** occurrenceDetails: http://janzen.sas.upenn.edu; catalogNumber: DHJPAR0007165; recordedBy: D.H. Janzen, W. Hallwachs & Roster Moraga; individualID: DHJPAR0007165; individualCount: 1; sex: male; lifeStage: adult; preparations: pinned; otherCatalogNumbers: ASTAV407-06, 06-SRNP-20306, BOLD:AAD6317; **Taxon:** scientificName: Vibrissina randycurtisi; phylum: Arthropoda; class: Insecta; order: Diptera; family: Tachinidae; genus: Vibrissina; specificEpithet: randycurtisi; scientificNameAuthorship: Fleming & Wood, 2016; **Location:** continent: Central America; country: Costa Rica; countryCode: CR; stateProvince: Guanacaste; county: Sector Del Oro; locality: Area de Conservación Guanacaste; verbatimLocality: Quebrada Raiz; verbatimElevation: 280; verbatimLatitude: 11.0287; verbatimLongitude: -85.4867; verbatimCoordinateSystem: Decimal; decimalLatitude: 11.0287; decimalLongitude: -85.4867; **Identification:** identifiedBy: AJ Fleming; dateIdentified: 2016; **Event:** samplingProtocol: Reared from the larvae of the Argid sawfly, Sericoceros gibbus; verbatimEventDate: 27-Feb-2006; **Record Level:** language: en; institutionCode: CNC; collectionCode: Insects; basisOfRecord: Pinned Specimen
- **Type status:** Paratype. **Occurrence:** occurrenceDetails: http://janzen.sas.upenn.edu; catalogNumber: DHJPAR0007163; recordedBy: D.H. Janzen, W. Hallwachs & Roster Moraga; individualID: DHJPAR0007163; individualCount: 1; sex: female; lifeStage: adult; preparations: pinned; otherCatalogNumbers: ASTAV405-06, 06-SRNP-20397, BOLD:AAD6317; **Taxon:** scientificName: Vibrissina randycurtisi; phylum: Arthropoda; class: Insecta; order: Diptera; family: Tachinidae; genus: Vibrissina; specificEpithet: randycurtisi; scientificNameAuthorship: Fleming & Wood, 2016; **Location:** continent: Central America; country: Costa Rica; countryCode: CR; stateProvince: Guanacaste; county: Sector Del Oro; locality: Area de Conservación Guanacaste; verbatimLocality: Quebrada Raiz; verbatimElevation: 280; verbatimLatitude: 11.0287; verbatimLongitude: -85.4867; verbatimCoordinateSystem: Decimal; decimalLatitude: 11.0287; decimalLongitude: -85.4867; **Identification:** identifiedBy: AJ Fleming; dateIdentified: 2016; **Event:** samplingProtocol: Reared from the larvae of the Argid sawfly, Sericoceros gibbus; verbatimEventDate: 10-Feb-2006; **Record Level:** language: en; institutionCode: CNC; collectionCode: Insects; basisOfRecord: Pinned Specimen
- **Type status:** Paratype. **Occurrence:** occurrenceDetails: http://janzen.sas.upenn.edu; catalogNumber: DHJPAR0007161; recordedBy: D.H. Janzen, W. Hallwachs & Roster Moraga; individualID: DHJPAR0007161; individualCount: 1; sex: female; lifeStage: adult; preparations: pinned; otherCatalogNumbers: ASTAV403-06, 06-SRNP-20392; **Taxon:** scientificName: Vibrissina randycurtisi; phylum: Arthropoda; class: Insecta; order: Diptera; family: Tachinidae; genus: Vibrissina; specificEpithet: randycurtisi; scientificNameAuthorship: Fleming & Wood, 2016; **Location:** continent: Central America; country: Costa Rica; countryCode: CR; stateProvince: Guanacaste; county: Sector Del Oro; locality: Area de Conservación Guanacaste; verbatimLocality: Quebrada Raiz; verbatimElevation: 280; verbatimLatitude: 11.0287; verbatimLongitude: -85.4867; verbatimCoordinateSystem: Decimal; decimalLatitude: 11.0287; decimalLongitude: -85.4867; **Identification:** identifiedBy: AJ Fleming; dateIdentified: 2016; **Event:** samplingProtocol: Reared from the larvae of the Argid sawfly, Sericoceros gibbus; verbatimEventDate: 09-Feb-2006; **Record Level:** language: en; institutionCode: CNC; collectionCode: Insects; basisOfRecord: Pinned Specimen

#### Description

**Male** (Fig. 6a, b, c). Length: 7–8mm. **Head** (Fig. 6b): parafrontal golden yellow tomentose; parafacial, face, gena and postorbit golden yellow tomentose; postgena silver tomentose; antenna black-brown; arista reddish brown basally, black apically; gena 0.17X eye height; 3 reclinate orbital bristles; 3 lower frontal bristles, below the level of the arista, lowest reaching level of first flagellomere; first flagellomere shorter than facial margin by length of pedicel. **Thorax** (Fig. 6a, c): dorsum gold tomentose with 4 distinct dorsal vittae visible presuturally, postsuturally all blurred together into a black smudge; scutellum black except along outer margin where it is gold tomentose; 3 strong katepisternal bristles; anepisternum gold tomentose with silver margins; anepimeron bearing gold tinge on anterior 1/3; 1 pair of discal scutellar bristles. Legs: black on all segments. Wings: smoky gray, bearing 2–3 short setulae at the base of R4+5. **Abdomen** (Fig. 6a): ST1+2 and T3 with light orange-brown ground color on sides; ST1+2 all black, mid-dorsal depression extending to margin of syntergite; anterior margins of T3 and T4 bearing gold tomentum over ⅓ of tergal surface; T5 with gold tomentum over 1/2 or more; T3 and T4 bearing 3 pairs of median discal bristles in addition to 1 pair of median marginal bristles; T5 bearing 2 rows of discal bristles, and 1 row of marginal bristles. **Male terminalia** (Fig. 7): sternite 5 with two prominent rounded lobes, flanking a wide U-shaped median cleft; posterior lobes 0.48X length of anterior plate; inner margin covered in dense tomentum, covering approximately half of the surface area of each lobe; posterior lobes bearing short stout bristles throughout; anterior plate bare, subequal in width to posterior lobes; cerci subtriangular in dorsal view, 1.5X as long as wide, separated along entire length; in lateral view cerci dorsally straight, apically rounded, each cercus appearing blunt and blade-like; postgonite parallel-sided and rounded at tip when viewed laterally, entire structure curving along midpoint giving it a rounded C shaped appearance; anterobasal heel slightly rounded, bearing few minute cilia; ventral sclerite of distiphallus linear along basal half, terminating in a strongly dilated and rounded apex; membranous acrophallus displaying a strongly angled apex when viewed laterally.

**Female** (Fig. 6d, e, f). Length: 5–6mm. As male, with the exception of the following characters: **abdomen**: abdominal tomentum silver-gray; abdomen ventrolaterally flattened, lacking discal bristles; mid-ventral portion of T3–T5 abdominal tergites with a row of strong stout spines.

#### Diagnosis

*Vibrissina randycurtisi* **sp. n.** can be differentiated from its congeners by the combination of the following traits: parafacial and parafrontal all gold; ground color of T5 black in both sexes; abdominal tomentum covering over 30% of ST1+2, T3, and T4; females bearing only 1 pair of marginal bristles on segments ST1+2 and T3, and a single row of marginal bristles on T4.

#### Etymology

*Vibrissina randycurtisi* **sp. n.** is dedicated to Mr. Randy Curtis of Arlington, Virginia in recognition of his seminal support in acquiring the land on which the INBio national biodiversity inventory collections grew and thrived since their founding in 1989, and still do, now as part of Museo Nacional de Costa Rica.

#### Distribution

Costa Rica, ACG, Prov. Guanacaste. Rain forest, in a dry-rain lowland intergrade at 280m.

#### Ecology

**Hosts**: reared seven times from the larvae of the sawfly *Sericoceros gibbus* (Klug) (Argidae), feeding on the leaves of *Coccoloba tuerckheimii* Donn. Sm. (Polygonaceae).

### Vibrissina randyjonesi

Fleming & Wood sp. n.

urn:lsid:zoobank.org:act:7B512E3E-F4AC-4922-8C7A-F456246D1473

#### Materials

- **Type status:** Holotype. **Occurrence:** occurrenceDetails: http://janzen.sas.upenn.edu; catalogNumber: DHJPAR0052639; recordedBy: D.H. Janzen, W. Hallwachs & Guillermo Pereira; individualID: DHJPAR0052639; individualCount: 1; sex: male; lifeStage: adult; preparations: pinned; otherCatalogNumbers: ASHYM1993-13, 13-SRNP-18684, BOLD:AAC2768; **Taxon:** scientificName: Vibrissina randyjonesi; phylum: Arthropoda; class: Insecta; order: Diptera; family: Tachinidae; genus: Vibrissina; specificEpithet: randyjonesi; scientificNameAuthorship: Fleming & Wood, 2016; **Location:** continent: Central America; country: Costa Rica; countryCode: CR; stateProvince: Guanacaste; county: Sector Santa Rosa; locality: Area de Conservación Guanacaste; verbatimLocality: Area Administrativa; verbatimElevation: 295; verbatimLatitude: 10.8376; verbatimLongitude: -85.6187; verbatimCoordinateSystem: Decimal; decimalLatitude: 10.8376; decimalLongitude: -85.6187; **Identification:** identifiedBy: AJ Fleming; dateIdentified: 2016; **Event:** samplingProtocol: Reared from the larvae of the Argid sawfly, Durgoa mattogrossensis; verbatimEventDate: 16-Aug-2013; **Record Level:** language: en; institutionCode: CNC; collectionCode: Insects; basisOfRecord: Pinned Specimen
- **Type status:** Paratype. **Occurrence:** occurrenceDetails: http://janzen.sas.upenn.edu; catalogNumber: DHJPAR0053332; recordedBy: D.H. Janzen, W. Hallwachs & Johan Vargas; individualID: DHJPAR0053332; individualCount: 1; sex: female; lifeStage: adult; preparations: pinned; otherCatalogNumbers: ASHYM2686-13, 13-SRNP-18696, BOLD:AAC2768; **Taxon:** scientificName: Vibrissina randyjonesi; phylum: Arthropoda; class: Insecta; order: Diptera; family: Tachinidae; genus: Vibrissina; specificEpithet: randyjonesi; scientificNameAuthorship: Fleming & Wood, 2016; **Location:** continent: Central America; country: Costa Rica; countryCode: CR; stateProvince: Guanacaste; county: Sector Santa Rosa; locality: Area de Conservación Guanacaste; verbatimLocality: Area Administrativa; verbatimElevation: 295; verbatimLatitude: 10.8376; verbatimLongitude: -85.6187; verbatimCoordinateSystem: Decimal; decimalLatitude: 10.8376; decimalLongitude: -85.6187; **Identification:** identifiedBy: AJ Fleming; dateIdentified: 2016; **Event:** samplingProtocol: Reared from the larvae of the Argid sawfly, Durgoa mattogrossensis; verbatimEventDate: 23-Aug-2013; **Record Level:** language: en; institutionCode: CNC; collectionCode: Insects; basisOfRecord: Pinned Specimen
- **Type status:** Paratype. **Occurrence:** occurrenceDetails: http://janzen.sas.upenn.edu; catalogNumber: DHJPAR0053313; recordedBy: D.H. Janzen, W. Hallwachs & Johan Vargas; individualID: DHJPAR0053313; individualCount: 1; sex: female; lifeStage: adult; preparations: pinned; otherCatalogNumbers: ASHYM2667-13, 13-SRNP-18794, BOLD:AAC2768; **Taxon:** scientificName: Vibrissina randyjonesi; phylum: Arthropoda; class: Insecta; order: Diptera; family: Tachinidae; genus: Vibrissina; specificEpithet: randyjonesi; scientificNameAuthorship: Fleming & Wood, 2016; **Location:** continent: Central America; country: Costa Rica; countryCode: CR; stateProvince: Guanacaste; county: Sector Santa Rosa; locality: Area de Conservación Guanacaste; verbatimLocality: Area Administrativa; verbatimElevation: 295; verbatimLatitude: 10.8376; verbatimLongitude: -85.6187; verbatimCoordinateSystem: Decimal; decimalLatitude: 10.8376; decimalLongitude: -85.6187; **Identification:** identifiedBy: AJ Fleming; dateIdentified: 2016; **Event:** samplingProtocol: Reared from the larvae of the Argid sawfly, Durgoa mattogrossensis; verbatimEventDate: 02-Sep-2013; **Record Level:** language: en; institutionCode: CNC; collectionCode: Insects; basisOfRecord: Pinned Specimen
- **Type status:** Paratype. **Occurrence:** occurrenceDetails: http://janzen.sas.upenn.edu; catalogNumber: DHJPAR0053317; recordedBy: D.H. Janzen, W. Hallwachs & Guillermo Pereira; individualID: DHJPAR0053317; individualCount: 1; sex: male; lifeStage: adult; preparations: pinned; otherCatalogNumbers: ASHYM2671-13, 13-SRNP-18862, BOLD:AAC2768; **Taxon:** scientificName: Vibrissina randyjonesi; phylum: Arthropoda; class: Insecta; order: Diptera; family: Tachinidae; genus: Vibrissina; specificEpithet: randyjonesi; scientificNameAuthorship: Fleming & Wood, 2016; **Location:** continent: Central America; country: Costa Rica; countryCode: CR; stateProvince: Guanacaste; county: Sector Santa Rosa; locality: Area de Conservación Guanacaste; verbatimLocality: Area Administrativa; verbatimElevation: 295; verbatimLatitude: 10.8376; verbatimLongitude: -85.6187; verbatimCoordinateSystem: Decimal; decimalLatitude: 10.8376; decimalLongitude: -85.6187; **Identification:** identifiedBy: AJ Fleming; dateIdentified: 2016; **Event:** samplingProtocol: Reared from the larvae of the Argid sawfly, Durgoa mattogrossensis; verbatimEventDate: 01-Sep-2013; **Record Level:** language: en; institutionCode: CNC; collectionCode: Insects; basisOfRecord: Pinned Specimen
- **Type status:** Paratype. **Occurrence:** occurrenceDetails: http://janzen.sas.upenn.edu; catalogNumber: DHJPAR0053299; recordedBy: D.H. Janzen, W. Hallwachs & Guillermo Pereira; individualID: DHJPAR0053299; individualCount: 1; sex: female; lifeStage: adult; preparations: pinned; otherCatalogNumbers: ASHYM2653-13, 13-SRNP-18844, BOLD:AAC2768; **Taxon:** scientificName: Vibrissina randyjonesi; phylum: Arthropoda; class: Insecta; order: Diptera; family: Tachinidae; genus: Vibrissina; specificEpithet: randyjonesi; scientificNameAuthorship: Fleming & Wood, 2016; **Location:** continent: Central America; country: Costa Rica; countryCode: CR; stateProvince: Guanacaste; county: Sector Santa Rosa; locality: Area de Conservación Guanacaste; verbatimLocality: Area Administrativa; verbatimElevation: 295; verbatimLatitude: 10.8376; verbatimLongitude: -85.6187; verbatimCoordinateSystem: Decimal; decimalLatitude: 10.8376; decimalLongitude: -85.6187; **Identification:** identifiedBy: AJ Fleming; dateIdentified: 2016; **Event:** samplingProtocol: Reared from the larvae of the Argid sawfly, Durgoa mattogrossensis; verbatimEventDate: 23-Sep-2013; **Record Level:** language: en; institutionCode: CNC; collectionCode: Insects; basisOfRecord: Pinned Specimen
- **Type status:** Paratype. **Occurrence:** occurrenceDetails: http://janzen.sas.upenn.edu; catalogNumber: DHJPAR0052643; recordedBy: D.H. Janzen, W. Hallwachs & Guillermo Pereira; individualID: DHJPAR0052643; individualCount: 1; sex: male; lifeStage: adult; preparations: pinned; otherCatalogNumbers: ASHYM1997-13, 13-SRNP-18653, BOLD:AAC2768; **Taxon:** scientificName: Vibrissina randyjonesi; phylum: Arthropoda; class: Insecta; order: Diptera; family: Tachinidae; genus: Vibrissina; specificEpithet: randyjonesi; scientificNameAuthorship: Fleming & Wood, 2016; **Location:** continent: Central America; country: Costa Rica; countryCode: CR; stateProvince: Guanacaste; county: Sector Santa Rosa; locality: Area de Conservación Guanacaste; verbatimLocality: Area Administrativa; verbatimElevation: 295; verbatimLatitude: 10.8376; verbatimLongitude: -85.6187; verbatimCoordinateSystem: Decimal; decimalLatitude: 10.8376; decimalLongitude: -85.6187; **Identification:** identifiedBy: AJ Fleming; dateIdentified: 2016; **Event:** samplingProtocol: Reared from the larvae of the Argid sawfly, Durgoa mattogrossensis; verbatimEventDate: 11-Aug-2013; **Record Level:** language: en; institutionCode: CNC; collectionCode: Insects; basisOfRecord: Pinned Specimen
- **Type status:** Paratype. **Occurrence:** occurrenceDetails: http://janzen.sas.upenn.edu; catalogNumber: DHJPAR0052657; recordedBy: D.H. Janzen, W. Hallwachs & Guillermo Pereira; individualID: DHJPAR0052657; individualCount: 1; sex: male; lifeStage: adult; preparations: pinned; otherCatalogNumbers: ASHYM2011-13, 13-SRNP-18727, BOLD:AAC2768; **Taxon:** scientificName: Vibrissina randyjonesi; phylum: Arthropoda; class: Insecta; order: Diptera; family: Tachinidae; genus: Vibrissina; specificEpithet: randyjonesi; scientificNameAuthorship: Fleming & Wood, 2016; **Location:** continent: Central America; country: Costa Rica; countryCode: CR; stateProvince: Guanacaste; county: Sector Santa Rosa; locality: Area de Conservación Guanacaste; verbatimLocality: Area Administrativa; verbatimElevation: 295; verbatimLatitude: 10.8376; verbatimLongitude: -85.6187; verbatimCoordinateSystem: Decimal; decimalLatitude: 10.8376; decimalLongitude: -85.6187; **Identification:** identifiedBy: AJ Fleming; dateIdentified: 2016; **Event:** samplingProtocol: Reared from the larvae of the Argid sawfly, Durgoa mattogrossensis; verbatimEventDate: 02-Aug-2013; **Record Level:** language: en; institutionCode: CNC; collectionCode: Insects; basisOfRecord: Pinned Specimen
- **Type status:** Paratype. **Occurrence:** occurrenceDetails: http://janzen.sas.upenn.edu; catalogNumber: DHJPAR0052636; recordedBy: D.H. Janzen, W. Hallwachs & Guillermo Pereira; individualID: DHJPAR0052636; individualCount: 1; sex: female; lifeStage: adult; preparations: pinned; otherCatalogNumbers: ASHYM1990-13, 13-SRNP-18626, BOLD:AAC2768; **Taxon:** scientificName: Vibrissina randyjonesi; phylum: Arthropoda; class: Insecta; order: Diptera; family: Tachinidae; genus: Vibrissina; specificEpithet: randyjonesi; scientificNameAuthorship: Fleming & Wood, 2016; **Location:** continent: Central America; country: Costa Rica; countryCode: CR; stateProvince: Guanacaste; county: Sector Santa Rosa; locality: Area de Conservación Guanacaste; verbatimLocality: Area Administrativa; verbatimElevation: 295; verbatimLatitude: 10.8376; verbatimLongitude: -85.6187; verbatimCoordinateSystem: Decimal; decimalLatitude: 10.8376; decimalLongitude: -85.6187; **Identification:** identifiedBy: AJ Fleming; dateIdentified: 2016; **Event:** samplingProtocol: Reared from the larvae of the Argid sawfly, Durgoa mattogrossensis; verbatimEventDate: 14-Aug-2013; **Record Level:** language: en; institutionCode: CNC; collectionCode: Insects; basisOfRecord: Pinned Specimen
- **Type status:** Paratype. **Occurrence:** occurrenceDetails: http://janzen.sas.upenn.edu; catalogNumber: DHJPAR0052637; recordedBy: D.H. Janzen, W. Hallwachs & Guillermo Pereira; individualID: DHJPAR0052637; individualCount: 1; sex: male; lifeStage: adult; preparations: pinned; otherCatalogNumbers: ASHYM1991-13, 13-SRNP-18679, BOLD:AAC2768; **Taxon:** scientificName: Vibrissina randyjonesi; phylum: Arthropoda; class: Insecta; order: Diptera; family: Tachinidae; genus: Vibrissina; specificEpithet: randyjonesi; scientificNameAuthorship: Fleming & Wood, 2016; **Location:** continent: Central America; country: Costa Rica; countryCode: CR; stateProvince: Guanacaste; county: Sector Santa Rosa; locality: Area de Conservación Guanacaste; verbatimLocality: Area Administrativa; verbatimElevation: 295; verbatimLatitude: 10.8376; verbatimLongitude: -85.6187; verbatimCoordinateSystem: Decimal; decimalLatitude: 10.8376; decimalLongitude: -85.6187; **Identification:** identifiedBy: AJ Fleming; dateIdentified: 2016; **Event:** samplingProtocol: Reared from the larvae of the Argid sawfly, Durgoa mattogrossensis; verbatimEventDate: 14-Aug-2013; **Record Level:** language: en; institutionCode: CNC; collectionCode: Insects; basisOfRecord: Pinned Specimen
- **Type status:** Paratype. **Occurrence:** occurrenceDetails: http://janzen.sas.upenn.edu; catalogNumber: DHJPAR0017871; recordedBy: D.H. Janzen, W. Hallwachs & gusaneros; individualID: DHJPAR0017871; individualCount: 1; sex: female; lifeStage: adult; preparations: pinned; otherCatalogNumbers: ASTAR581-07, 99-SRNP-9589, BOLD:AAC2768; **Taxon:** scientificName: Vibrissina randyjonesi; phylum: Arthropoda; class: Insecta; order: Diptera; family: Tachinidae; genus: Vibrissina; specificEpithet: randyjonesi; scientificNameAuthorship: Fleming & Wood, 2016; **Location:** continent: Central America; country: Costa Rica; countryCode: CR; stateProvince: Guanacaste; county: Sector Santa Rosa; locality: Area de Conservación Guanacaste; verbatimLocality: Area Administrativa; verbatimElevation: 295; verbatimLatitude: 10.8376; verbatimLongitude: -85.6187; verbatimCoordinateSystem: Decimal; decimalLatitude: 10.8376; decimalLongitude: -85.6187; **Identification:** identifiedBy: AJ Fleming; dateIdentified: 2016; **Event:** samplingProtocol: Reared from the larvae of the Argid sawfly, Durgoa mattogrossensis; verbatimEventDate: 08-Oct-1999; **Record Level:** language: en; institutionCode: CNC; collectionCode: Insects; basisOfRecord: Pinned Specimen
- **Type status:** Paratype. **Occurrence:** occurrenceDetails: http://janzen.sas.upenn.edu; catalogNumber: DHJPAR0053301; recordedBy: D.H. Janzen, W. Hallwachs & Guillermo Pereira; individualID: DHJPAR0053301; individualCount: 1; sex: female; lifeStage: adult; preparations: pinned; otherCatalogNumbers: ASHYM2655-13, 13-SRNP-18824, BOLD:AAC2768; **Taxon:** scientificName: Vibrissina randyjonesi; phylum: Arthropoda; class: Insecta; order: Diptera; family: Tachinidae; genus: Vibrissina; specificEpithet: randyjonesi; scientificNameAuthorship: Fleming & Wood, 2016; **Location:** continent: Central America; country: Costa Rica; countryCode: CR; stateProvince: Guanacaste; county: Sector Santa Rosa; locality: Area de Conservación Guanacaste; verbatimLocality: Area Administrativa; verbatimElevation: 295; verbatimLatitude: 10.8376; verbatimLongitude: -85.6187; verbatimCoordinateSystem: Decimal; decimalLatitude: 10.8376; decimalLongitude: -85.6187; **Identification:** identifiedBy: AJ Fleming; dateIdentified: 2016; **Event:** samplingProtocol: Reared from the larvae of the Argid sawfly, Durgoa mattogrossensis; verbatimEventDate: 05-Sep-2013; **Record Level:** language: en; institutionCode: CNC; collectionCode: Insects; basisOfRecord: Pinned Specimen
- **Type status:** Paratype. **Occurrence:** occurrenceDetails: http://janzen.sas.upenn.edu; catalogNumber: DHJPAR0052549; recordedBy: D.H. Janzen, W. Hallwachs & Johan Vargas; individualID: DHJPAR0052549; individualCount: 1; sex: female; lifeStage: adult; preparations: pinned; otherCatalogNumbers: ASHYM1903-13, 13-SRNP-18703, BOLD:AAC2768; **Taxon:** scientificName: Vibrissina randyjonesi; phylum: Arthropoda; class: Insecta; order: Diptera; family: Tachinidae; genus: Vibrissina; specificEpithet: randyjonesi; scientificNameAuthorship: Fleming & Wood, 2016; **Location:** continent: Central America; country: Costa Rica; countryCode: CR; stateProvince: Guanacaste; county: Sector Santa Rosa; locality: Area de Conservación Guanacaste; verbatimLocality: Area Administrativa; verbatimElevation: 295; verbatimLatitude: 10.8376; verbatimLongitude: -85.6187; verbatimCoordinateSystem: Decimal; decimalLatitude: 10.8376; decimalLongitude: -85.6187; **Identification:** identifiedBy: AJ Fleming; dateIdentified: 2016; **Event:** samplingProtocol: Reared from the larvae of the Argid sawfly, Durgoa mattogrossensis; verbatimEventDate: 17-Aug-2013; **Record Level:** language: en; institutionCode: CNC; collectionCode: Insects; basisOfRecord: Pinned Specimen
- **Type status:** Paratype. **Occurrence:** occurrenceDetails: http://janzen.sas.upenn.edu; catalogNumber: DHJPAR0017872; recordedBy: D.H. Janzen, W. Hallwachs & gusaneros; individualID: DHJPAR0017872; individualCount: 1; sex: female; lifeStage: adult; preparations: pinned; otherCatalogNumbers: ASTAR582-07, 99-SRNP-9817, BOLD:AAC2768; **Taxon:** scientificName: Vibrissina randyjonesi; phylum: Arthropoda; class: Insecta; order: Diptera; family: Tachinidae; genus: Vibrissina; specificEpithet: randyjonesi; scientificNameAuthorship: Fleming & Wood, 2016; **Location:** continent: Central America; country: Costa Rica; countryCode: CR; stateProvince: Guanacaste; county: Sector Santa Rosa; locality: Area de Conservación Guanacaste; verbatimLocality: Area Administrativa; verbatimElevation: 295; verbatimLatitude: 10.8376; verbatimLongitude: -85.6187; verbatimCoordinateSystem: Decimal; decimalLatitude: 10.8376; decimalLongitude: -85.6187; **Identification:** identifiedBy: AJ Fleming; dateIdentified: 2016; **Event:** samplingProtocol: Reared from the larvae of the Argid sawfly, Durgoa mattogrossensis; verbatimEventDate: 08-Jan-1999; **Record Level:** language: en; institutionCode: CNC; collectionCode: Insects; basisOfRecord: Pinned Specimen
- **Type status:** Paratype. **Occurrence:** occurrenceDetails: http://janzen.sas.upenn.edu; catalogNumber: DHJPAR0053315; recordedBy: D.H. Janzen, W. Hallwachs & Guillermo Pereira; individualID: DHJPAR0053315; individualCount: 1; sex: male; lifeStage: adult; preparations: pinned; otherCatalogNumbers: ASHYM2669-13, 13-SRNP-18666, BOLD:AAC2768; **Taxon:** scientificName: Vibrissina randyjonesi; phylum: Arthropoda; class: Insecta; order: Diptera; family: Tachinidae; genus: Vibrissina; specificEpithet: randyjonesi; scientificNameAuthorship: Fleming & Wood, 2016; **Location:** continent: Central America; country: Costa Rica; countryCode: CR; stateProvince: Guanacaste; county: Sector Santa Rosa; locality: Area de Conservación Guanacaste; verbatimLocality: Area Administrativa; verbatimElevation: 295; verbatimLatitude: 10.8376; verbatimLongitude: -85.6187; verbatimCoordinateSystem: Decimal; decimalLatitude: 10.8376; decimalLongitude: -85.6187; **Identification:** identifiedBy: AJ Fleming; dateIdentified: 2016; **Event:** samplingProtocol: Reared from the larvae of the Argid sawfly, Durgoa mattogrossensis; verbatimEventDate: 29-Aug-2013; **Record Level:** language: en; institutionCode: CNC; collectionCode: Insects; basisOfRecord: Pinned Specimen
- **Type status:** Paratype. **Occurrence:** occurrenceDetails: http://janzen.sas.upenn.edu; catalogNumber: DHJPAR0052649; recordedBy: D.H. Janzen, W. Hallwachs & Guillermo Pereira; individualID: DHJPAR0052649; individualCount: 1; sex: female; lifeStage: adult; preparations: pinned; otherCatalogNumbers: ASHYM2003-13, 13-SRNP-18648, BOLD:AAC2768; **Taxon:** scientificName: Vibrissina randyjonesi; phylum: Arthropoda; class: Insecta; order: Diptera; family: Tachinidae; genus: Vibrissina; specificEpithet: randyjonesi; scientificNameAuthorship: Fleming & Wood, 2016; **Location:** continent: Central America; country: Costa Rica; countryCode: CR; stateProvince: Guanacaste; county: Sector Santa Rosa; locality: Area de Conservación Guanacaste; verbatimLocality: Area Administrativa; verbatimElevation: 295; verbatimLatitude: 10.8376; verbatimLongitude: -85.6187; verbatimCoordinateSystem: Decimal; decimalLatitude: 10.8376; decimalLongitude: -85.6187; **Identification:** identifiedBy: AJ Fleming; dateIdentified: 2016; **Event:** samplingProtocol: Reared from the larvae of the Argid sawfly, Durgoa mattogrossensis; verbatimEventDate: 11-Aug-2013; **Record Level:** language: en; institutionCode: CNC; collectionCode: Insects; basisOfRecord: Pinned Specimen
- **Type status:** Paratype. **Occurrence:** occurrenceDetails: http://janzen.sas.upenn.edu; catalogNumber: DHJPAR0053319; recordedBy: D.H. Janzen, W. Hallwachs & Guillermo Pereira; individualID: DHJPAR0053319; individualCount: 1; sex: male; lifeStage: adult; preparations: pinned; otherCatalogNumbers: ASHYM2673-13, 13-SRNP-18848, BOLD:AAC2768; **Taxon:** scientificName: Vibrissina randyjonesi; phylum: Arthropoda; class: Insecta; order: Diptera; family: Tachinidae; genus: Vibrissina; specificEpithet: randyjonesi; scientificNameAuthorship: Fleming & Wood, 2016; **Location:** continent: Central America; country: Costa Rica; countryCode: CR; stateProvince: Guanacaste; county: Sector Santa Rosa; locality: Area de Conservación Guanacaste; verbatimLocality: Area Administrativa; verbatimElevation: 295; verbatimLatitude: 10.8376; verbatimLongitude: -85.6187; verbatimCoordinateSystem: Decimal; decimalLatitude: 10.8376; decimalLongitude: -85.6187; **Identification:** identifiedBy: AJ Fleming; dateIdentified: 2016; **Event:** samplingProtocol: Reared from the larvae of the Argid sawfly, Durgoa mattogrossensis; verbatimEventDate: 27-Aug-2013; **Record Level:** language: en; institutionCode: CNC; collectionCode: Insects; basisOfRecord: Pinned Specimen
- **Type status:** Paratype. **Occurrence:** occurrenceDetails: http://janzen.sas.upenn.edu; catalogNumber: DHJPAR0053340; recordedBy: D.H. Janzen, W. Hallwachs & Johan Vargas; individualID: DHJPAR0053340; individualCount: 1; sex: female; lifeStage: adult; preparations: pinned; otherCatalogNumbers: ASHYM2694-13, 13-SRNP-18778, BOLD:AAC2768; **Taxon:** scientificName: Vibrissina randyjonesi; phylum: Arthropoda; class: Insecta; order: Diptera; family: Tachinidae; genus: Vibrissina; specificEpithet: randyjonesi; scientificNameAuthorship: Fleming & Wood, 2016; **Location:** continent: Central America; country: Costa Rica; countryCode: CR; stateProvince: Guanacaste; county: Sector Santa Rosa; locality: Area de Conservación Guanacaste; verbatimLocality: Area Administrativa; verbatimElevation: 295; verbatimLatitude: 10.8376; verbatimLongitude: -85.6187; verbatimCoordinateSystem: Decimal; decimalLatitude: 10.8376; decimalLongitude: -85.6187; **Identification:** identifiedBy: AJ Fleming; dateIdentified: 2016; **Event:** samplingProtocol: Reared from the larvae of the Argid sawfly, Durgoa mattogrossensis; verbatimEventDate: 24-Aug-2013; **Record Level:** language: en; institutionCode: CNC; collectionCode: Insects; basisOfRecord: Pinned Specimen
- **Type status:** Paratype. **Occurrence:** occurrenceDetails: http://janzen.sas.upenn.edu; catalogNumber: DHJPAR0052519; recordedBy: D.H. Janzen, W. Hallwachs & Guillermo Pereira; individualID: DHJPAR0052519; individualCount: 1; sex: male; lifeStage: adult; preparations: pinned; otherCatalogNumbers: ASHYM1873-13, 13-SRNP-18875, BOLD:AAC2768; **Taxon:** scientificName: Vibrissina randyjonesi; phylum: Arthropoda; class: Insecta; order: Diptera; family: Tachinidae; genus: Vibrissina; specificEpithet: randyjonesi; scientificNameAuthorship: Fleming & Wood, 2016; **Location:** continent: Central America; country: Costa Rica; countryCode: CR; stateProvince: Guanacaste; county: Sector Santa Rosa; locality: Area de Conservación Guanacaste; verbatimLocality: Area Administrativa; verbatimElevation: 295; verbatimLatitude: 10.8376; verbatimLongitude: -85.6187; verbatimCoordinateSystem: Decimal; decimalLatitude: 10.8376; decimalLongitude: -85.6187; **Identification:** identifiedBy: AJ Fleming; dateIdentified: 2016; **Event:** samplingProtocol: Reared from the larvae of the Argid sawfly, Durgoa mattogrossensis; verbatimEventDate: 17-Aug-2013; **Record Level:** language: en; institutionCode: CNC; collectionCode: Insects; basisOfRecord: Pinned Specimen
- **Type status:** Paratype. **Occurrence:** occurrenceDetails: http://janzen.sas.upenn.edu; catalogNumber: DHJPAR0053316; recordedBy: D.H. Janzen, W. Hallwachs & Johan Vargas; individualID: DHJPAR0053316; individualCount: 1; sex: female; lifeStage: adult; preparations: pinned; otherCatalogNumbers: ASHYM2670-13, 13-SRNP-18784, BOLD:AAC2768; **Taxon:** scientificName: Vibrissina randyjonesi; phylum: Arthropoda; class: Insecta; order: Diptera; family: Tachinidae; genus: Vibrissina; specificEpithet: randyjonesi; scientificNameAuthorship: Fleming & Wood, 2016; **Location:** continent: Central America; country: Costa Rica; countryCode: CR; stateProvince: Guanacaste; county: Sector Santa Rosa; locality: Area de Conservación Guanacaste; verbatimLocality: Area Administrativa; verbatimElevation: 295; verbatimLatitude: 10.8376; verbatimLongitude: -85.6187; verbatimCoordinateSystem: Decimal; decimalLatitude: 10.8376; decimalLongitude: -85.6187; **Identification:** identifiedBy: AJ Fleming; dateIdentified: 2016; **Event:** samplingProtocol: Reared from the larvae of the Argid sawfly, Durgoa mattogrossensis; verbatimEventDate: 01-Sep-2013; **Record Level:** language: en; institutionCode: CNC; collectionCode: Insects; basisOfRecord: Pinned Specimen
- **Type status:** Paratype. **Occurrence:** occurrenceDetails: http://janzen.sas.upenn.edu; catalogNumber: DHJPAR0052600; recordedBy: D.H. Janzen, W. Hallwachs & Guillermo Pereira; individualID: DHJPAR0052600; individualCount: 1; sex: male; lifeStage: adult; preparations: pinned; otherCatalogNumbers: ASHYM1954-13, 13-SRNP-18631, BOLD:AAC2768; **Taxon:** scientificName: Vibrissina randyjonesi; phylum: Arthropoda; class: Insecta; order: Diptera; family: Tachinidae; genus: Vibrissina; specificEpithet: randyjonesi; scientificNameAuthorship: Fleming & Wood, 2016; **Location:** continent: Central America; country: Costa Rica; countryCode: CR; stateProvince: Guanacaste; county: Sector Santa Rosa; locality: Area de Conservación Guanacaste; verbatimLocality: Area Administrativa; verbatimElevation: 295; verbatimLatitude: 10.8376; verbatimLongitude: -85.6187; verbatimCoordinateSystem: Decimal; decimalLatitude: 10.8376; decimalLongitude: -85.6187; **Identification:** identifiedBy: AJ Fleming; dateIdentified: 2016; **Event:** samplingProtocol: Reared from the larvae of the Argid sawfly, Durgoa mattogrossensis; verbatimEventDate: 10-Aug-2013; **Record Level:** language: en; institutionCode: CNC; collectionCode: Insects; basisOfRecord: Pinned Specimen
- **Type status:** Paratype. **Occurrence:** occurrenceDetails: http://janzen.sas.upenn.edu; catalogNumber: DHJPAR0053300; recordedBy: D.H. Janzen, W. Hallwachs & Guillermo Pereira; individualID: DHJPAR0053300; individualCount: 1; sex: female; lifeStage: adult; preparations: pinned; otherCatalogNumbers: ASHYM2654-13, 13-SRNP-18828, BOLD:AAC2768; **Taxon:** scientificName: Vibrissina randyjonesi; phylum: Arthropoda; class: Insecta; order: Diptera; family: Tachinidae; genus: Vibrissina; specificEpithet: randyjonesi; scientificNameAuthorship: Fleming & Wood, 2016; **Location:** continent: Central America; country: Costa Rica; countryCode: CR; stateProvince: Guanacaste; county: Sector Santa Rosa; locality: Area de Conservación Guanacaste; verbatimLocality: Area Administrativa; verbatimElevation: 295; verbatimLatitude: 10.8376; verbatimLongitude: -85.6187; verbatimCoordinateSystem: Decimal; decimalLatitude: 10.8376; decimalLongitude: -85.6187; **Identification:** identifiedBy: AJ Fleming; dateIdentified: 2016; **Event:** samplingProtocol: Reared from the larvae of the Argid sawfly, Durgoa mattogrossensis; verbatimEventDate: 20-Sep-2013; **Record Level:** language: en; institutionCode: CNC; collectionCode: Insects; basisOfRecord: Pinned Specimen
- **Type status:** Paratype. **Occurrence:** occurrenceDetails: http://janzen.sas.upenn.edu; catalogNumber: DHJPAR0017883; recordedBy: D.H. Janzen, W. Hallwachs & gusaneros; individualID: DHJPAR0017883; individualCount: 1; sex: female; lifeStage: adult; preparations: pinned; otherCatalogNumbers: ASTAR593-07, 99-SRNP-9982, BOLD:AAC2768; **Taxon:** scientificName: Vibrissina randyjonesi; phylum: Arthropoda; class: Insecta; order: Diptera; family: Tachinidae; genus: Vibrissina; specificEpithet: randyjonesi; scientificNameAuthorship: Fleming & Wood, 2016; **Location:** continent: Central America; country: Costa Rica; countryCode: CR; stateProvince: Guanacaste; county: Sector Santa Rosa; locality: Area de Conservación Guanacaste; verbatimLocality: Parcela John Sullivan; verbatimElevation: 280; verbatimLatitude: 10.8734; verbatimLongitude: -85.6265; verbatimCoordinateSystem: Decimal; decimalLatitude: 10.8734; decimalLongitude: -85.6265; **Identification:** identifiedBy: AJ Fleming; dateIdentified: 2016; **Event:** samplingProtocol: Reared from the larvae of the Argid sawfly, Durgoa mattogrossensis; verbatimEventDate: 24-Jun-2000; **Record Level:** language: en; institutionCode: CNC; collectionCode: Insects; basisOfRecord: Pinned Specimen
- **Type status:** Paratype. **Occurrence:** occurrenceDetails: http://janzen.sas.upenn.edu; catalogNumber: DHJPAR0052517; recordedBy: D.H. Janzen, W. Hallwachs & Guillermo Pereira; individualID: DHJPAR0052517; individualCount: 1; sex: female; lifeStage: adult; preparations: pinned; otherCatalogNumbers: ASHYM1871-13, 13-SRNP-18765, BOLD:AAC2768; **Taxon:** scientificName: Vibrissina randyjonesi; phylum: Arthropoda; class: Insecta; order: Diptera; family: Tachinidae; genus: Vibrissina; specificEpithet: randyjonesi; scientificNameAuthorship: Fleming & Wood, 2016; **Location:** continent: Central America; country: Costa Rica; countryCode: CR; stateProvince: Guanacaste; county: Sector Santa Rosa; locality: Area de Conservación Guanacaste; verbatimLocality: Area Administrativa; verbatimElevation: 295; verbatimLatitude: 10.8376; verbatimLongitude: -85.6187; verbatimCoordinateSystem: Decimal; decimalLatitude: 10.8376; decimalLongitude: -85.6187; **Identification:** identifiedBy: AJ Fleming; dateIdentified: 2016; **Event:** samplingProtocol: Reared from the larvae of the Argid sawfly, Durgoa mattogrossensis; verbatimEventDate: 19-Aug-2013; **Record Level:** language: en; institutionCode: CNC; collectionCode: Insects; basisOfRecord: Pinned Specimen
- **Type status:** Paratype. **Occurrence:** occurrenceDetails: http://janzen.sas.upenn.edu; catalogNumber: DHJPAR0053312; recordedBy: D.H. Janzen, W. Hallwachs & Guillermo Pereira; individualID: DHJPAR0053312; individualCount: 1; sex: male; lifeStage: adult; preparations: pinned; otherCatalogNumbers: ASHYM2666-13, 13-SRNP-18854, BOLD:AAC2768; **Taxon:** scientificName: Vibrissina randyjonesi; phylum: Arthropoda; class: Insecta; order: Diptera; family: Tachinidae; genus: Vibrissina; specificEpithet: randyjonesi; scientificNameAuthorship: Fleming & Wood, 2016; **Location:** continent: Central America; country: Costa Rica; countryCode: CR; stateProvince: Guanacaste; county: Sector Santa Rosa; locality: Area de Conservación Guanacaste; verbatimLocality: Area Administrativa; verbatimElevation: 295; verbatimLatitude: 10.8376; verbatimLongitude: -85.6187; verbatimCoordinateSystem: Decimal; decimalLatitude: 10.8376; decimalLongitude: -85.6187; **Identification:** identifiedBy: AJ Fleming; dateIdentified: 2016; **Event:** samplingProtocol: Reared from the larvae of the Argid sawfly, Durgoa mattogrossensis; verbatimEventDate: 31-Aug-2013; **Record Level:** language: en; institutionCode: CNC; collectionCode: Insects; basisOfRecord: Pinned Specimen
- **Type status:** Paratype. **Occurrence:** occurrenceDetails: http://janzen.sas.upenn.edu; catalogNumber: DHJPAR0052632; recordedBy: D.H. Janzen, W. Hallwachs & Guillermo Pereira; individualID: DHJPAR0052632; individualCount: 1; sex: male; lifeStage: adult; preparations: pinned; otherCatalogNumbers: ASHYM1986-13, 13-SRNP-18623, BOLD:AAC2768; **Taxon:** scientificName: Vibrissina randyjonesi; phylum: Arthropoda; class: Insecta; order: Diptera; family: Tachinidae; genus: Vibrissina; specificEpithet: randyjonesi; scientificNameAuthorship: Fleming & Wood, 2016; **Location:** continent: Central America; country: Costa Rica; countryCode: CR; stateProvince: Guanacaste; county: Sector Santa Rosa; locality: Area de Conservación Guanacaste; verbatimLocality: Area Administrativa; verbatimElevation: 295; verbatimLatitude: 10.8376; verbatimLongitude: -85.6187; verbatimCoordinateSystem: Decimal; decimalLatitude: 10.8376; decimalLongitude: -85.6187; **Identification:** identifiedBy: AJ Fleming; dateIdentified: 2016; **Event:** samplingProtocol: Reared from the larvae of the Argid sawfly, Durgoa mattogrossensis; verbatimEventDate: 04-Aug-2013; **Record Level:** language: en; institutionCode: CNC; collectionCode: Insects; basisOfRecord: Pinned Specimen
- **Type status:** Paratype. **Occurrence:** occurrenceDetails: http://janzen.sas.upenn.edu; catalogNumber: DHJPAR0053324; recordedBy: D.H. Janzen, W. Hallwachs & Johan Vargas; individualID: DHJPAR0053324; individualCount: 1; sex: male; lifeStage: adult; preparations: pinned; otherCatalogNumbers: ASHYM2678-13, 13-SRNP-18776, BOLD:AAC2768; **Taxon:** scientificName: Vibrissina randyjonesi; phylum: Arthropoda; class: Insecta; order: Diptera; family: Tachinidae; genus: Vibrissina; specificEpithet: randyjonesi; scientificNameAuthorship: Fleming & Wood, 2016; **Location:** continent: Central America; country: Costa Rica; countryCode: CR; stateProvince: Guanacaste; county: Sector Santa Rosa; locality: Area de Conservación Guanacaste; verbatimLocality: Area Administrativa; verbatimElevation: 295; verbatimLatitude: 10.8376; verbatimLongitude: -85.6187; verbatimCoordinateSystem: Decimal; decimalLatitude: 10.8376; decimalLongitude: -85.6187; **Identification:** identifiedBy: AJ Fleming; dateIdentified: 2016; **Event:** samplingProtocol: Reared from the larvae of the Argid sawfly, Durgoa mattogrossensis; verbatimEventDate: 22-Aug-2013; **Record Level:** language: en; institutionCode: CNC; collectionCode: Insects; basisOfRecord: Pinned Specimen
- **Type status:** Paratype. **Occurrence:** occurrenceDetails: http://janzen.sas.upenn.edu; catalogNumber: DHJPAR0052606; recordedBy: D.H. Janzen, W. Hallwachs & Guillermo Pereira; individualID: DHJPAR0052606; individualCount: 1; sex: male; lifeStage: adult; preparations: pinned; otherCatalogNumbers: ASHYM1960-13, 13-SRNP-18627, BOLD:AAC2768; **Taxon:** scientificName: Vibrissina randyjonesi; phylum: Arthropoda; class: Insecta; order: Diptera; family: Tachinidae; genus: Vibrissina; specificEpithet: randyjonesi; scientificNameAuthorship: Fleming & Wood, 2016; **Location:** continent: Central America; country: Costa Rica; countryCode: CR; stateProvince: Guanacaste; county: Sector Santa Rosa; locality: Area de Conservación Guanacaste; verbatimLocality: Area Administrativa; verbatimElevation: 295; verbatimLatitude: 10.8376; verbatimLongitude: -85.6187; verbatimCoordinateSystem: Decimal; decimalLatitude: 10.8376; decimalLongitude: -85.6187; **Identification:** identifiedBy: AJ Fleming; dateIdentified: 2016; **Event:** samplingProtocol: Reared from the larvae of the Argid sawfly, Durgoa mattogrossensis; verbatimEventDate: 08-Aug-2013; **Record Level:** language: en; institutionCode: CNC; collectionCode: Insects; basisOfRecord: Pinned Specimen
- **Type status:** Paratype. **Occurrence:** occurrenceDetails: http://janzen.sas.upenn.edu; catalogNumber: DHJPAR0017876; recordedBy: D.H. Janzen, W. Hallwachs & gusaneros; individualID: DHJPAR0017876; individualCount: 1; sex: female; lifeStage: adult; preparations: pinned; otherCatalogNumbers: ASTAR586-07, 99-SRNP-10114, BOLD:AAC2768; **Taxon:** scientificName: Vibrissina randyjonesi; phylum: Arthropoda; class: Insecta; order: Diptera; family: Tachinidae; genus: Vibrissina; specificEpithet: randyjonesi; scientificNameAuthorship: Fleming & Wood, 2016; **Location:** continent: Central America; country: Costa Rica; countryCode: CR; stateProvince: Guanacaste; county: Sector Santa Rosa; locality: Area de Conservación Guanacaste; verbatimLocality: Parcela John Sullivan; verbatimElevation: 280; verbatimLatitude: 10.8734; verbatimLongitude: -85.6265; verbatimCoordinateSystem: Decimal; decimalLatitude: 10.8734; decimalLongitude: -85.6265; **Identification:** identifiedBy: AJ Fleming; dateIdentified: 2016; **Event:** samplingProtocol: Reared from the larvae of the Argid sawfly, Durgoa mattogrossensis; verbatimEventDate: 31-Jul-1999; **Record Level:** language: en; institutionCode: CNC; collectionCode: Insects; basisOfRecord: Pinned Specimen
- **Type status:** Paratype. **Occurrence:** occurrenceDetails: http://janzen.sas.upenn.edu; catalogNumber: DHJPAR0052640; recordedBy: D.H. Janzen, W. Hallwachs & Guillermo Pereira; individualID: DHJPAR0052640; individualCount: 1; sex: female; lifeStage: adult; preparations: pinned; otherCatalogNumbers: ASHYM1994-13, 13-SRNP-18674, BOLD:AAC2768; **Taxon:** scientificName: Vibrissina randyjonesi; phylum: Arthropoda; class: Insecta; order: Diptera; family: Tachinidae; genus: Vibrissina; specificEpithet: randyjonesi; scientificNameAuthorship: Fleming & Wood, 2016; **Location:** continent: Central America; country: Costa Rica; countryCode: CR; stateProvince: Guanacaste; county: Sector Santa Rosa; locality: Area de Conservación Guanacaste; verbatimLocality: Area Administrativa; verbatimElevation: 295; verbatimLatitude: 10.8376; verbatimLongitude: -85.6187; verbatimCoordinateSystem: Decimal; decimalLatitude: 10.8376; decimalLongitude: -85.6187; **Identification:** identifiedBy: AJ Fleming; dateIdentified: 2016; **Event:** samplingProtocol: Reared from the larvae of the Argid sawfly, Durgoa mattogrossensis; verbatimEventDate: 11-Aug-2013; **Record Level:** language: en; institutionCode: CNC; collectionCode: Insects; basisOfRecord: Pinned Specimen
- **Type status:** Paratype. **Occurrence:** occurrenceDetails: http://janzen.sas.upenn.edu; catalogNumber: DHJPAR0052524; recordedBy: D.H. Janzen, W. Hallwachs & Guillermo Pereira; individualID: DHJPAR0052524; individualCount: 1; sex: female; lifeStage: adult; preparations: pinned; otherCatalogNumbers: ASHYM1878-13, 13-SRNP-18739, BOLD:AAC2768; **Taxon:** scientificName: Vibrissina randyjonesi; phylum: Arthropoda; class: Insecta; order: Diptera; family: Tachinidae; genus: Vibrissina; specificEpithet: randyjonesi; scientificNameAuthorship: Fleming & Wood, 2016; **Location:** continent: Central America; country: Costa Rica; countryCode: CR; stateProvince: Guanacaste; county: Sector Santa Rosa; locality: Area de Conservación Guanacaste; verbatimLocality: Area Administrativa; verbatimElevation: 295; verbatimLatitude: 10.8376; verbatimLongitude: -85.6187; verbatimCoordinateSystem: Decimal; decimalLatitude: 10.8376; decimalLongitude: -85.6187; **Identification:** identifiedBy: AJ Fleming; dateIdentified: 2016; **Event:** samplingProtocol: Reared from the larvae of the Argid sawfly, Durgoa mattogrossensis; verbatimEventDate: 20-Aug-2013; **Record Level:** language: en; institutionCode: CNC; collectionCode: Insects; basisOfRecord: Pinned Specimen
- **Type status:** Paratype. **Occurrence:** occurrenceDetails: http://janzen.sas.upenn.edu; catalogNumber: DHJPAR0053314; recordedBy: D.H. Janzen, W. Hallwachs & Guillermo Pereira; individualID: DHJPAR0053314; individualCount: 1; sex: female; lifeStage: adult; preparations: pinned; otherCatalogNumbers: ASHYM2668-13, 13-SRNP-18830, BOLD:AAC2768; **Taxon:** scientificName: Vibrissina randyjonesi; phylum: Arthropoda; class: Insecta; order: Diptera; family: Tachinidae; genus: Vibrissina; specificEpithet: randyjonesi; scientificNameAuthorship: Fleming & Wood, 2016; **Location:** continent: Central America; country: Costa Rica; countryCode: CR; stateProvince: Guanacaste; county: Sector Santa Rosa; locality: Area de Conservación Guanacaste; verbatimLocality: Area Administrativa; verbatimElevation: 295; verbatimLatitude: 10.8376; verbatimLongitude: -85.6187; verbatimCoordinateSystem: Decimal; decimalLatitude: 10.8376; decimalLongitude: -85.6187; **Identification:** identifiedBy: AJ Fleming; dateIdentified: 2016; **Event:** samplingProtocol: Reared from the larvae of the Argid sawfly, Durgoa mattogrossensis; verbatimEventDate: 01-Sep-2013; **Record Level:** language: en; institutionCode: CNC; collectionCode: Insects; basisOfRecord: Pinned Specimen
- **Type status:** Paratype. **Occurrence:** occurrenceDetails: http://janzen.sas.upenn.edu; catalogNumber: DHJPAR0052651; recordedBy: D.H. Janzen, W. Hallwachs & Guillermo Pereira; individualID: DHJPAR0052651; individualCount: 1; sex: male; lifeStage: adult; preparations: pinned; otherCatalogNumbers: ASHYM2005-13, 13-SRNP-18874, BOLD:AAC2768; **Taxon:** scientificName: Vibrissina randyjonesi; phylum: Arthropoda; class: Insecta; order: Diptera; family: Tachinidae; genus: Vibrissina; specificEpithet: randyjonesi; scientificNameAuthorship: Fleming & Wood, 2016; **Location:** continent: Central America; country: Costa Rica; countryCode: CR; stateProvince: Guanacaste; county: Sector Santa Rosa; locality: Area de Conservación Guanacaste; verbatimLocality: Area Administrativa; verbatimElevation: 295; verbatimLatitude: 10.8376; verbatimLongitude: -85.6187; verbatimCoordinateSystem: Decimal; decimalLatitude: 10.8376; decimalLongitude: -85.6187; **Identification:** identifiedBy: AJ Fleming; dateIdentified: 2016; **Event:** samplingProtocol: Reared from the larvae of the Argid sawfly, Durgoa mattogrossensis; **Record Level:** language: en; institutionCode: CNC; collectionCode: Insects; basisOfRecord: Pinned Specimen
- **Type status:** Paratype. **Occurrence:** occurrenceDetails: http://janzen.sas.upenn.edu; catalogNumber: DHJPAR0053329; recordedBy: D.H. Janzen, W. Hallwachs & Johan Vargas; individualID: DHJPAR0053329; individualCount: 1; sex: female; lifeStage: adult; preparations: pinned; otherCatalogNumbers: ASHYM2683-13, 13-SRNP-18709, BOLD:AAC2768; **Taxon:** scientificName: Vibrissina randyjonesi; phylum: Arthropoda; class: Insecta; order: Diptera; family: Tachinidae; genus: Vibrissina; specificEpithet: randyjonesi; scientificNameAuthorship: Fleming & Wood, 2016; **Location:** continent: Central America; country: Costa Rica; countryCode: CR; stateProvince: Guanacaste; county: Sector Santa Rosa; locality: Area de Conservación Guanacaste; verbatimLocality: Area Administrativa; verbatimElevation: 295; verbatimLatitude: 10.8376; verbatimLongitude: -85.6187; verbatimCoordinateSystem: Decimal; decimalLatitude: 10.8376; decimalLongitude: -85.6187; **Identification:** identifiedBy: AJ Fleming; dateIdentified: 2016; **Event:** samplingProtocol: Reared from the larvae of the Argid sawfly, Durgoa mattogrossensis; verbatimEventDate: 23-Aug-2013; **Record Level:** language: en; institutionCode: CNC; collectionCode: Insects; basisOfRecord: Pinned Specimen
- **Type status:** Paratype. **Occurrence:** occurrenceDetails: http://janzen.sas.upenn.edu; catalogNumber: DHJPAR0017877; recordedBy: D.H. Janzen, W. Hallwachs & gusaneros; individualID: DHJPAR0017877; individualCount: 1; sex: female; lifeStage: adult; preparations: pinned; otherCatalogNumbers: ASTAR587-07, 99-SRNP-10207,; **Taxon:** scientificName: Vibrissina randyjonesi; phylum: Arthropoda; class: Insecta; order: Diptera; family: Tachinidae; genus: Vibrissina; specificEpithet: randyjonesi; scientificNameAuthorship: Fleming & Wood, 2016; **Location:** continent: Central America; country: Costa Rica; countryCode: CR; stateProvince: Guanacaste; county: Sector Santa Rosa; locality: Area de Conservación Guanacaste; verbatimLocality: Parcela John Sullivan; verbatimElevation: 280; verbatimLatitude: 10.8734; verbatimLongitude: -85.6265; verbatimCoordinateSystem: Decimal; decimalLatitude: 10.8734; decimalLongitude: -85.6265; **Identification:** identifiedBy: AJ Fleming; dateIdentified: 2016; **Event:** samplingProtocol: Reared from the larvae of the Argid sawfly, Durgoa mattogrossensis; verbatimEventDate: 08-Feb-1999; **Record Level:** language: en; institutionCode: CNC; collectionCode: Insects; basisOfRecord: Pinned Specimen
- **Type status:** Paratype. **Occurrence:** occurrenceDetails: http://janzen.sas.upenn.edu; catalogNumber: DHJPAR0053311; recordedBy: D.H. Janzen, W. Hallwachs & Johan Vargas; individualID: DHJPAR0053311; individualCount: 1; sex: male; lifeStage: adult; preparations: pinned; otherCatalogNumbers: ASHYM2665-13, 13-SRNP-18812, BOLD:AAC2768; **Taxon:** scientificName: Vibrissina randyjonesi; phylum: Arthropoda; class: Insecta; order: Diptera; family: Tachinidae; genus: Vibrissina; specificEpithet: randyjonesi; scientificNameAuthorship: Fleming & Wood, 2016; **Location:** continent: Central America; country: Costa Rica; countryCode: CR; stateProvince: Guanacaste; county: Sector Santa Rosa; locality: Area de Conservación Guanacaste; verbatimLocality: Area Administrativa; verbatimElevation: 295; verbatimLatitude: 10.8376; verbatimLongitude: -85.6187; verbatimCoordinateSystem: Decimal; decimalLatitude: 10.8376; decimalLongitude: -85.6187; **Identification:** identifiedBy: AJ Fleming; dateIdentified: 2016; **Event:** samplingProtocol: Reared from the larvae of the Argid sawfly, Durgoa mattogrossensis; verbatimEventDate: 08-Sep-2013; **Record Level:** language: en; institutionCode: CNC; collectionCode: Insects; basisOfRecord: Pinned Specimen
- **Type status:** Paratype. **Occurrence:** occurrenceDetails: http://janzen.sas.upenn.edu; catalogNumber: DHJPAR0052516; recordedBy: D.H. Janzen, W. Hallwachs & Guillermo Pereira; individualID: DHJPAR0052516; individualCount: 1; sex: male; lifeStage: adult; preparations: pinned; otherCatalogNumbers: ASHYM1870-13, 13-SRNP-18881, BOLD:AAC2768; **Taxon:** scientificName: Vibrissina randyjonesi; phylum: Arthropoda; class: Insecta; order: Diptera; family: Tachinidae; genus: Vibrissina; specificEpithet: randyjonesi; scientificNameAuthorship: Fleming & Wood, 2016; **Location:** continent: Central America; country: Costa Rica; countryCode: CR; stateProvince: Guanacaste; county: Sector Santa Rosa; locality: Area de Conservación Guanacaste; verbatimLocality: Area Administrativa; verbatimElevation: 295; verbatimLatitude: 10.8376; verbatimLongitude: -85.6187; verbatimCoordinateSystem: Decimal; decimalLatitude: 10.8376; decimalLongitude: -85.6187; **Identification:** identifiedBy: AJ Fleming; dateIdentified: 2016; **Event:** samplingProtocol: Reared from the larvae of the Argid sawfly, Durgoa mattogrossensis; verbatimEventDate: 19-Aug-2013; **Record Level:** language: en; institutionCode: CNC; collectionCode: Insects; basisOfRecord: Pinned Specimen
- **Type status:** Paratype. **Occurrence:** occurrenceDetails: http://janzen.sas.upenn.edu; catalogNumber: DHJPAR0053310; recordedBy: D.H. Janzen, W. Hallwachs & Johan Vargas; individualID: DHJPAR0053310; individualCount: 1; sex: female; lifeStage: adult; preparations: pinned; otherCatalogNumbers: ASHYM2664-13, 13-SRNP-18722, BOLD:AAC2768; **Taxon:** scientificName: Vibrissina randyjonesi; phylum: Arthropoda; class: Insecta; order: Diptera; family: Tachinidae; genus: Vibrissina; specificEpithet: randyjonesi; scientificNameAuthorship: Fleming & Wood, 2016; **Location:** continent: Central America; country: Costa Rica; countryCode: CR; stateProvince: Guanacaste; county: Sector Santa Rosa; locality: Area de Conservación Guanacaste; verbatimLocality: Area Administrativa; verbatimElevation: 295; verbatimLatitude: 10.8376; verbatimLongitude: -85.6187; verbatimCoordinateSystem: Decimal; decimalLatitude: 10.8376; decimalLongitude: -85.6187; **Identification:** identifiedBy: AJ Fleming; dateIdentified: 2016; **Event:** samplingProtocol: Reared from the larvae of the Argid sawfly, Durgoa mattogrossensis; verbatimEventDate: 04-Sep-2013; **Record Level:** language: en; institutionCode: CNC; collectionCode: Insects; basisOfRecord: Pinned Specimen
- **Type status:** Paratype. **Occurrence:** occurrenceDetails: http://janzen.sas.upenn.edu; catalogNumber: DHJPAR0053325; recordedBy: D.H. Janzen, W. Hallwachs & Johan Vargas; individualID: DHJPAR0053325; individualCount: 1; sex: male; lifeStage: adult; preparations: pinned; otherCatalogNumbers: ASHYM2679-13, 13-SRNP-18781, BOLD:AAC2768; **Taxon:** scientificName: Vibrissina randyjonesi; phylum: Arthropoda; class: Insecta; order: Diptera; family: Tachinidae; genus: Vibrissina; specificEpithet: randyjonesi; scientificNameAuthorship: Fleming & Wood, 2016; **Location:** continent: Central America; country: Costa Rica; countryCode: CR; stateProvince: Guanacaste; county: Sector Santa Rosa; locality: Area de Conservación Guanacaste; verbatimLocality: Area Administrativa; verbatimElevation: 295; verbatimLatitude: 10.8376; verbatimLongitude: -85.6187; verbatimCoordinateSystem: Decimal; decimalLatitude: 10.8376; decimalLongitude: -85.6187; **Identification:** identifiedBy: AJ Fleming; dateIdentified: 2016; **Event:** samplingProtocol: Reared from the larvae of the Argid sawfly, Durgoa mattogrossensis; verbatimEventDate: 24-Aug-2013; **Record Level:** language: en; institutionCode: CNC; collectionCode: Insects; basisOfRecord: Pinned Specimen
- **Type status:** Paratype. **Occurrence:** occurrenceDetails: http://janzen.sas.upenn.edu; catalogNumber: DHJPAR0017873; recordedBy: D.H. Janzen, W. Hallwachs & gusaneros; individualID: DHJPAR0017873; individualCount: 1; sex: male; lifeStage: adult; preparations: pinned; otherCatalogNumbers: ASTAR583-07, 99-SRNP-10137, BOLD:AAC2768; **Taxon:** scientificName: Vibrissina randyjonesi; phylum: Arthropoda; class: Insecta; order: Diptera; family: Tachinidae; genus: Vibrissina; specificEpithet: randyjonesi; scientificNameAuthorship: Fleming & Wood, 2016; **Location:** continent: Central America; country: Costa Rica; countryCode: CR; stateProvince: Guanacaste; county: Sector Santa Rosa; locality: Area de Conservación Guanacaste; verbatimLocality: Parcela John Sullivan; verbatimElevation: 280; verbatimLatitude: 10.8734; verbatimLongitude: -85.6265; verbatimCoordinateSystem: Decimal; decimalLatitude: 10.8734; decimalLongitude: -85.6265; **Identification:** identifiedBy: AJ Fleming; dateIdentified: 2016; **Event:** samplingProtocol: Reared from the larvae of the Argid sawfly, Durgoa mattogrossensis; verbatimEventDate: 08-Sep-1999; **Record Level:** language: en; institutionCode: CNC; collectionCode: Insects; basisOfRecord: Pinned Specimen
- **Type status:** Paratype. **Occurrence:** occurrenceDetails: http://janzen.sas.upenn.edu; catalogNumber: DHJPAR0053305; recordedBy: D.H. Janzen, W. Hallwachs & Johan Vargas; individualID: DHJPAR0053305; individualCount: 1; sex: female; lifeStage: adult; preparations: pinned; otherCatalogNumbers: ASHYM2659-13, 13-SRNP-18713, BOLD:AAC2768; **Taxon:** scientificName: Vibrissina randyjonesi; phylum: Arthropoda; class: Insecta; order: Diptera; family: Tachinidae; genus: Vibrissina; specificEpithet: randyjonesi; scientificNameAuthorship: Fleming & Wood, 2016; **Location:** continent: Central America; country: Costa Rica; countryCode: CR; stateProvince: Guanacaste; county: Sector Santa Rosa; locality: Area de Conservación Guanacaste; verbatimLocality: Area Administrativa; verbatimElevation: 295; verbatimLatitude: 10.8376; verbatimLongitude: -85.6187; verbatimCoordinateSystem: Decimal; decimalLatitude: 10.8376; decimalLongitude: -85.6187; **Identification:** identifiedBy: AJ Fleming; dateIdentified: 2016; **Event:** samplingProtocol: Reared from the larvae of the Argid sawfly, Durgoa mattogrossensis; verbatimEventDate: 18-Sep-2013; **Record Level:** language: en; institutionCode: CNC; collectionCode: Insects; basisOfRecord: Pinned Specimen
- **Type status:** Paratype. **Occurrence:** occurrenceDetails: http://janzen.sas.upenn.edu; catalogNumber: DHJPAR0017879; recordedBy: D.H. Janzen, W. Hallwachs & gusaneros; individualID: DHJPAR0017879; individualCount: 1; sex: male; lifeStage: adult; preparations: pinned; otherCatalogNumbers: ASTAR589-07, 99-SRNP-10237, BOLD:AAC2768; **Taxon:** scientificName: Vibrissina randyjonesi; phylum: Arthropoda; class: Insecta; order: Diptera; family: Tachinidae; genus: Vibrissina; specificEpithet: randyjonesi; scientificNameAuthorship: Fleming & Wood, 2016; **Location:** continent: Central America; country: Costa Rica; countryCode: CR; stateProvince: Guanacaste; county: Sector Santa Rosa; locality: Area de Conservación Guanacaste; verbatimLocality: Parcela John Sullivan; verbatimElevation: 280; verbatimLatitude: 10.8734; verbatimLongitude: -85.6265; verbatimCoordinateSystem: Decimal; decimalLatitude: 10.8734; decimalLongitude: -85.6265; **Identification:** identifiedBy: AJ Fleming; dateIdentified: 2016; **Event:** samplingProtocol: Reared from the larvae of the Argid sawfly, Durgoa mattogrossensis; verbatimEventDate: 29-Jun-2000; **Record Level:** language: en; institutionCode: CNC; collectionCode: Insects; basisOfRecord: Pinned Specimen
- **Type status:** Paratype. **Occurrence:** occurrenceDetails: http://janzen.sas.upenn.edu; catalogNumber: DHJPAR0017881; recordedBy: D.H. Janzen, W. Hallwachs & gusaneros; individualID: DHJPAR0017881; individualCount: 1; sex: male; lifeStage: adult; preparations: pinned; otherCatalogNumbers: ASTAR591-07, 99-SRNP-10053, BOLD:AAC2768; **Taxon:** scientificName: Vibrissina randyjonesi; phylum: Arthropoda; class: Insecta; order: Diptera; family: Tachinidae; genus: Vibrissina; specificEpithet: randyjonesi; scientificNameAuthorship: Fleming & Wood, 2016; **Location:** continent: Central America; country: Costa Rica; countryCode: CR; stateProvince: Guanacaste; county: Sector Santa Rosa; locality: Area de Conservación Guanacaste; verbatimLocality: Parcela John Sullivan; verbatimElevation: 280; verbatimLatitude: 10.8734; verbatimLongitude: -85.6265; verbatimCoordinateSystem: Decimal; decimalLatitude: 10.8734; decimalLongitude: -85.6265; **Identification:** identifiedBy: AJ Fleming; dateIdentified: 2016; **Event:** samplingProtocol: Reared from the larvae of the Argid sawfly, Durgoa mattogrossensis; verbatimEventDate: 23-Jun-2000; **Record Level:** language: en; institutionCode: CNC; collectionCode: Insects; basisOfRecord: Pinned Specimen
- **Type status:** Paratype. **Occurrence:** occurrenceDetails: http://janzen.sas.upenn.edu; catalogNumber: DHJPAR0053308; recordedBy: D.H. Janzen, W. Hallwachs & Guillermo Pereira; individualID: DHJPAR0053308; individualCount: 1; sex: male; lifeStage: adult; preparations: pinned; otherCatalogNumbers: ASHYM2662-13, 13-SRNP-18863, BOLD:AAC2768; **Taxon:** scientificName: Vibrissina randyjonesi; phylum: Arthropoda; class: Insecta; order: Diptera; family: Tachinidae; genus: Vibrissina; specificEpithet: randyjonesi; scientificNameAuthorship: Fleming & Wood, 2016; **Location:** continent: Central America; country: Costa Rica; countryCode: CR; stateProvince: Guanacaste; county: Sector Santa Rosa; locality: Area de Conservación Guanacaste; verbatimLocality: Area Administrativa; verbatimElevation: 295; verbatimLatitude: 10.8376; verbatimLongitude: -85.6187; verbatimCoordinateSystem: Decimal; decimalLatitude: 10.8376; decimalLongitude: -85.6187; **Identification:** identifiedBy: AJ Fleming; dateIdentified: 2016; **Event:** samplingProtocol: Reared from the larvae of the Argid sawfly, Durgoa mattogrossensis; verbatimEventDate: 31-Aug-2013; **Record Level:** language: en; institutionCode: CNC; collectionCode: Insects; basisOfRecord: Pinned Specimen
- **Type status:** Paratype. **Occurrence:** occurrenceDetails: http://janzen.sas.upenn.edu; catalogNumber: DHJPAR0053321; recordedBy: D.H. Janzen, W. Hallwachs & Guillermo Pereira; individualID: DHJPAR0053321; individualCount: 1; sex: male; lifeStage: adult; preparations: pinned; otherCatalogNumbers: ASHYM2675-13, 13-SRNP-18852, BOLD:AAC2768; **Taxon:** scientificName: Vibrissina randyjonesi; phylum: Arthropoda; class: Insecta; order: Diptera; family: Tachinidae; genus: Vibrissina; specificEpithet: randyjonesi; scientificNameAuthorship: Fleming & Wood, 2016; **Location:** continent: Central America; country: Costa Rica; countryCode: CR; stateProvince: Guanacaste; county: Sector Santa Rosa; locality: Area de Conservación Guanacaste; verbatimLocality: Area Administrativa; verbatimElevation: 295; verbatimLatitude: 10.8376; verbatimLongitude: -85.6187; verbatimCoordinateSystem: Decimal; decimalLatitude: 10.8376; decimalLongitude: -85.6187; **Identification:** identifiedBy: AJ Fleming; dateIdentified: 2016; **Event:** samplingProtocol: Reared from the larvae of the Argid sawfly, Durgoa mattogrossensis; verbatimEventDate: 08-Sep-2013; **Record Level:** language: en; institutionCode: CNC; collectionCode: Insects; basisOfRecord: Pinned Specimen
- **Type status:** Paratype. **Occurrence:** occurrenceDetails: http://janzen.sas.upenn.edu; catalogNumber: DHJPAR0052532; recordedBy: D.H. Janzen, W. Hallwachs & Johan Vargas; individualID: DHJPAR0052532; individualCount: 1; sex: male; lifeStage: adult; preparations: pinned; otherCatalogNumbers: ASHYM1886-13, 13-SRNP-18706, BOLD:AAC2768; **Taxon:** scientificName: Vibrissina randyjonesi; phylum: Arthropoda; class: Insecta; order: Diptera; family: Tachinidae; genus: Vibrissina; specificEpithet: randyjonesi; scientificNameAuthorship: Fleming & Wood, 2016; **Location:** continent: Central America; country: Costa Rica; countryCode: CR; stateProvince: Guanacaste; county: Sector Santa Rosa; locality: Area de Conservación Guanacaste; verbatimLocality: Area Administrativa; verbatimElevation: 295; verbatimLatitude: 10.8376; verbatimLongitude: -85.6187; verbatimCoordinateSystem: Decimal; decimalLatitude: 10.8376; decimalLongitude: -85.6187; **Identification:** identifiedBy: AJ Fleming; dateIdentified: 2016; **Event:** samplingProtocol: Reared from the larvae of the Argid sawfly, Durgoa mattogrossensis; verbatimEventDate: 21-Aug-2013; **Record Level:** language: en; institutionCode: CNC; collectionCode: Insects; basisOfRecord: Pinned Specimen
- **Type status:** Paratype. **Occurrence:** occurrenceDetails: http://janzen.sas.upenn.edu; catalogNumber: DHJPAR0053307; recordedBy: D.H. Janzen, W. Hallwachs & Guillermo Pereira; individualID: DHJPAR0053307; individualCount: 1; sex: female; lifeStage: adult; preparations: pinned; otherCatalogNumbers: ASHYM2661-13, 13-SRNP-18754, BOLD:AAC2768; **Taxon:** scientificName: Vibrissina randyjonesi; phylum: Arthropoda; class: Insecta; order: Diptera; family: Tachinidae; genus: Vibrissina; specificEpithet: randyjonesi; scientificNameAuthorship: Fleming & Wood, 2016; **Location:** continent: Central America; country: Costa Rica; countryCode: CR; stateProvince: Guanacaste; county: Sector Santa Rosa; locality: Area de Conservación Guanacaste; verbatimLocality: Area Administrativa; verbatimElevation: 295; verbatimLatitude: 10.8376; verbatimLongitude: -85.6187; verbatimCoordinateSystem: Decimal; decimalLatitude: 10.8376; decimalLongitude: -85.6187; **Identification:** identifiedBy: AJ Fleming; dateIdentified: 2016; **Event:** samplingProtocol: Reared from the larvae of the Argid sawfly, Durgoa mattogrossensis; verbatimEventDate: 01-Sep-2013; **Record Level:** language: en; institutionCode: CNC; collectionCode: Insects; basisOfRecord: Pinned Specimen
- **Type status:** Paratype. **Occurrence:** occurrenceDetails: http://janzen.sas.upenn.edu; catalogNumber: DHJPAR0052604; recordedBy: D.H. Janzen, W. Hallwachs & Guillermo Pereira; individualID: DHJPAR0052604; individualCount: 1; sex: male; lifeStage: adult; preparations: pinned; otherCatalogNumbers: ASHYM1958-13, 13-SRNP-18642, BOLD:AAC2768; **Taxon:** scientificName: Vibrissina randyjonesi; phylum: Arthropoda; class: Insecta; order: Diptera; family: Tachinidae; genus: Vibrissina; specificEpithet: randyjonesi; scientificNameAuthorship: Fleming & Wood, 2016; **Location:** continent: Central America; country: Costa Rica; countryCode: CR; stateProvince: Guanacaste; county: Sector Santa Rosa; locality: Area de Conservación Guanacaste; verbatimLocality: Area Administrativa; verbatimElevation: 295; verbatimLatitude: 10.8376; verbatimLongitude: -85.6187; verbatimCoordinateSystem: Decimal; decimalLatitude: 10.8376; decimalLongitude: -85.6187; **Identification:** identifiedBy: AJ Fleming; dateIdentified: 2016; **Event:** samplingProtocol: Reared from the larvae of the Argid sawfly, Durgoa mattogrossensis; verbatimEventDate: 10-Aug-2013; **Record Level:** language: en; institutionCode: CNC; collectionCode: Insects; basisOfRecord: Pinned Specimen
- **Type status:** Paratype. **Occurrence:** occurrenceDetails: http://janzen.sas.upenn.edu; catalogNumber: DHJPAR0053302; recordedBy: D.H. Janzen, W. Hallwachs & Guillermo Pereira; individualID: DHJPAR0053302; individualCount: 1; sex: female; lifeStage: adult; preparations: pinned; otherCatalogNumbers: ASHYM2656-13, 13-SRNP-18763, BOLD:AAC2768; **Taxon:** scientificName: Vibrissina randyjonesi; phylum: Arthropoda; class: Insecta; order: Diptera; family: Tachinidae; genus: Vibrissina; specificEpithet: randyjonesi; scientificNameAuthorship: Fleming & Wood, 2016; **Location:** continent: Central America; country: Costa Rica; countryCode: CR; stateProvince: Guanacaste; county: Sector Santa Rosa; locality: Area de Conservación Guanacaste; verbatimLocality: Area Administrativa; verbatimElevation: 295; verbatimLatitude: 10.8376; verbatimLongitude: -85.6187; verbatimCoordinateSystem: Decimal; decimalLatitude: 10.8376; decimalLongitude: -85.6187; **Identification:** identifiedBy: AJ Fleming; dateIdentified: 2016; **Event:** samplingProtocol: Reared from the larvae of the Argid sawfly, Durgoa mattogrossensis; verbatimEventDate: 05-Sep-2013; **Record Level:** language: en; institutionCode: CNC; collectionCode: Insects; basisOfRecord: Pinned Specimen
- **Type status:** Paratype. **Occurrence:** occurrenceDetails: http://janzen.sas.upenn.edu; catalogNumber: DHJPAR0052601; recordedBy: D.H. Janzen, W. Hallwachs & Guillermo Pereira; individualID: DHJPAR0052601; individualCount: 1; sex: female; lifeStage: adult; preparations: pinned; otherCatalogNumbers: ASHYM1955-13, 13-SRNP-18677, BOLD:AAC2768; **Taxon:** scientificName: Vibrissina randyjonesi; phylum: Arthropoda; class: Insecta; order: Diptera; family: Tachinidae; genus: Vibrissina; specificEpithet: randyjonesi; scientificNameAuthorship: Fleming & Wood, 2016; **Location:** continent: Central America; country: Costa Rica; countryCode: CR; stateProvince: Guanacaste; county: Sector Santa Rosa; locality: Area de Conservación Guanacaste; verbatimLocality: Area Administrativa; verbatimElevation: 295; verbatimLatitude: 10.8376; verbatimLongitude: -85.6187; verbatimCoordinateSystem: Decimal; decimalLatitude: 10.8376; decimalLongitude: -85.6187; **Identification:** identifiedBy: AJ Fleming; dateIdentified: 2016; **Event:** samplingProtocol: Reared from the larvae of the Argid sawfly, Durgoa mattogrossensis; verbatimEventDate: 10-Aug-2013; **Record Level:** language: en; institutionCode: CNC; collectionCode: Insects; basisOfRecord: Pinned Specimen
- **Type status:** Paratype. **Occurrence:** occurrenceDetails: http://janzen.sas.upenn.edu; catalogNumber: DHJPAR0017880; recordedBy: D.H. Janzen, W. Hallwachs & gusaneros; individualID: DHJPAR0017880; individualCount: 1; sex: female; lifeStage: adult; preparations: pinned; otherCatalogNumbers: ASTAR590-07, 99-SRNP-10231,; **Taxon:** scientificName: Vibrissina randyjonesi; phylum: Arthropoda; class: Insecta; order: Diptera; family: Tachinidae; genus: Vibrissina; specificEpithet: randyjonesi; scientificNameAuthorship: Fleming & Wood, 2016; **Location:** continent: Central America; country: Costa Rica; countryCode: CR; stateProvince: Guanacaste; county: Sector Santa Rosa; locality: Area de Conservación Guanacaste; verbatimLocality: Parcela John Sullivan; verbatimElevation: 280; verbatimLatitude: 10.8734; verbatimLongitude: -85.6265; verbatimCoordinateSystem: Decimal; decimalLatitude: 10.8734; decimalLongitude: -85.6265; **Identification:** identifiedBy: AJ Fleming; dateIdentified: 2016; **Event:** samplingProtocol: Reared from the larvae of the Argid sawfly, Durgoa mattogrossensis; verbatimEventDate: 06-Oct-2000; **Record Level:** language: en; institutionCode: CNC; collectionCode: Insects; basisOfRecord: Pinned Specimen
- **Type status:** Paratype. **Occurrence:** occurrenceDetails: http://janzen.sas.upenn.edu; catalogNumber: DHJPAR0053320; recordedBy: D.H. Janzen, W. Hallwachs & Johan Vargas; individualID: DHJPAR0053320; individualCount: 1; sex: female; lifeStage: adult; preparations: pinned; otherCatalogNumbers: ASHYM2674-13, 13-SRNP-18777, BOLD:AAC2768; **Taxon:** scientificName: Vibrissina randyjonesi; phylum: Arthropoda; class: Insecta; order: Diptera; family: Tachinidae; genus: Vibrissina; specificEpithet: randyjonesi; scientificNameAuthorship: Fleming & Wood, 2016; **Location:** continent: Central America; country: Costa Rica; countryCode: CR; stateProvince: Guanacaste; county: Sector Santa Rosa; locality: Area de Conservación Guanacaste; verbatimLocality: Area Administrativa; verbatimElevation: 295; verbatimLatitude: 10.8376; verbatimLongitude: -85.6187; verbatimCoordinateSystem: Decimal; decimalLatitude: 10.8376; decimalLongitude: -85.6187; **Identification:** identifiedBy: AJ Fleming; dateIdentified: 2016; **Event:** samplingProtocol: Reared from the larvae of the Argid sawfly, Durgoa mattogrossensis; verbatimEventDate: 27-Aug-2013; **Record Level:** language: en; institutionCode: CNC; collectionCode: Insects; basisOfRecord: Pinned Specimen
- **Type status:** Paratype. **Occurrence:** occurrenceDetails: http://janzen.sas.upenn.edu; catalogNumber: DHJPAR0053304; recordedBy: D.H. Janzen, W. Hallwachs & Guillermo Pereira; individualID: DHJPAR0053304; individualCount: 1; sex: female; lifeStage: adult; preparations: pinned; otherCatalogNumbers: ASHYM2658-13, 13-SRNP-18837, BOLD:AAC2768; **Taxon:** scientificName: Vibrissina randyjonesi; phylum: Arthropoda; class: Insecta; order: Diptera; family: Tachinidae; genus: Vibrissina; specificEpithet: randyjonesi; scientificNameAuthorship: Fleming & Wood, 2016; **Location:** continent: Central America; country: Costa Rica; countryCode: CR; stateProvince: Guanacaste; county: Sector Santa Rosa; locality: Area de Conservación Guanacaste; verbatimLocality: Area Administrativa; verbatimElevation: 295; verbatimLatitude: 10.8376; verbatimLongitude: -85.6187; verbatimCoordinateSystem: Decimal; decimalLatitude: 10.8376; decimalLongitude: -85.6187; **Identification:** identifiedBy: AJ Fleming; dateIdentified: 2016; **Event:** samplingProtocol: Reared from the larvae of the Argid sawfly, Durgoa mattogrossensis; verbatimEventDate: 10-Sep-2013; **Record Level:** language: en; institutionCode: CNC; collectionCode: Insects; basisOfRecord: Pinned Specimen
- **Type status:** Paratype. **Occurrence:** occurrenceDetails: http://janzen.sas.upenn.edu; catalogNumber: DHJPAR0017878; recordedBy: D.H. Janzen, W. Hallwachs & gusaneros; individualID: DHJPAR0017878; individualCount: 1; sex: female; lifeStage: adult; preparations: pinned; otherCatalogNumbers: ASTAR588-07, 99-SRNP-9622, BOLD:AAC2768; **Taxon:** scientificName: Vibrissina randyjonesi; phylum: Arthropoda; class: Insecta; order: Diptera; family: Tachinidae; genus: Vibrissina; specificEpithet: randyjonesi; scientificNameAuthorship: Fleming & Wood, 2016; **Location:** continent: Central America; country: Costa Rica; countryCode: CR; stateProvince: Guanacaste; county: Sector Santa Rosa; locality: Area de Conservación Guanacaste; verbatimLocality: Area Administrativa; verbatimElevation: 295; verbatimLatitude: 10.8376; verbatimLongitude: -85.6187; verbatimCoordinateSystem: Decimal; decimalLatitude: 10.8376; decimalLongitude: -85.6187; **Identification:** identifiedBy: AJ Fleming; dateIdentified: 2016; **Event:** samplingProtocol: Reared from the larvae of the Argid sawfly, Durgoa mattogrossensis; verbatimEventDate: 30-Jul-1999; **Record Level:** language: en; institutionCode: CNC; collectionCode: Insects; basisOfRecord: Pinned Specimen
- **Type status:** Paratype. **Occurrence:** occurrenceDetails: http://janzen.sas.upenn.edu; catalogNumber: DHJPAR0052529; recordedBy: D.H. Janzen, W. Hallwachs & Johan Vargas; individualID: DHJPAR0052529; individualCount: 1; sex: male; lifeStage: adult; preparations: pinned; otherCatalogNumbers: ASHYM1883-13, 13-SRNP-18690, BOLD:AAC2768; **Taxon:** scientificName: Vibrissina randyjonesi; phylum: Arthropoda; class: Insecta; order: Diptera; family: Tachinidae; genus: Vibrissina; specificEpithet: randyjonesi; scientificNameAuthorship: Fleming & Wood, 2016; **Location:** continent: Central America; country: Costa Rica; countryCode: CR; stateProvince: Guanacaste; county: Sector Santa Rosa; locality: Area de Conservación Guanacaste; verbatimLocality: Area Administrativa; verbatimElevation: 295; verbatimLatitude: 10.8376; verbatimLongitude: -85.6187; verbatimCoordinateSystem: Decimal; decimalLatitude: 10.8376; decimalLongitude: -85.6187; **Identification:** identifiedBy: AJ Fleming; dateIdentified: 2016; **Event:** samplingProtocol: Reared from the larvae of the Argid sawfly, Durgoa mattogrossensis; verbatimEventDate: 21-Aug-2013; **Record Level:** language: en; institutionCode: CNC; collectionCode: Insects; basisOfRecord: Pinned Specimen
- **Type status:** Paratype. **Occurrence:** occurrenceDetails: http://janzen.sas.upenn.edu; catalogNumber: DHJPAR0053331; recordedBy: D.H. Janzen, W. Hallwachs & Guillermo Pereira; individualID: DHJPAR0053331; individualCount: 1; sex: male; lifeStage: adult; preparations: pinned; otherCatalogNumbers: ASHYM2685-13, 13-SRNP-18668, BOLD:AAC2768; **Taxon:** scientificName: Vibrissina randyjonesi; phylum: Arthropoda; class: Insecta; order: Diptera; family: Tachinidae; genus: Vibrissina; specificEpithet: randyjonesi; scientificNameAuthorship: Fleming & Wood, 2016; **Location:** continent: Central America; country: Costa Rica; countryCode: CR; stateProvince: Guanacaste; county: Sector Santa Rosa; locality: Area de Conservación Guanacaste; verbatimLocality: Area Administrativa; verbatimElevation: 295; verbatimLatitude: 10.8376; verbatimLongitude: -85.6187; verbatimCoordinateSystem: Decimal; decimalLatitude: 10.8376; decimalLongitude: -85.6187; **Identification:** identifiedBy: AJ Fleming; dateIdentified: 2016; **Event:** samplingProtocol: Reared from the larvae of the Argid sawfly, Durgoa mattogrossensis; verbatimEventDate: 22-Aug-2013; **Record Level:** language: en; institutionCode: CNC; collectionCode: Insects; basisOfRecord: Pinned Specimen
- **Type status:** Paratype. **Occurrence:** occurrenceDetails: http://janzen.sas.upenn.edu; catalogNumber: DHJPAR0052647; recordedBy: D.H. Janzen, W. Hallwachs & Guillermo Pereira; individualID: DHJPAR0052647; individualCount: 1; sex: female; lifeStage: adult; preparations: pinned; otherCatalogNumbers: ASHYM2001-13, 13-SRNP-18650, BOLD:AAC2768; **Taxon:** scientificName: Vibrissina randyjonesi; phylum: Arthropoda; class: Insecta; order: Diptera; family: Tachinidae; genus: Vibrissina; specificEpithet: randyjonesi; scientificNameAuthorship: Fleming & Wood, 2016; **Location:** continent: Central America; country: Costa Rica; countryCode: CR; stateProvince: Guanacaste; county: Sector Santa Rosa; locality: Area de Conservación Guanacaste; verbatimLocality: Area Administrativa; verbatimElevation: 295; verbatimLatitude: 10.8376; verbatimLongitude: -85.6187; verbatimCoordinateSystem: Decimal; decimalLatitude: 10.8376; decimalLongitude: -85.6187; **Identification:** identifiedBy: AJ Fleming; dateIdentified: 2016; **Event:** samplingProtocol: Reared from the larvae of the Argid sawfly, Durgoa mattogrossensis; verbatimEventDate: 17-Aug-2013; **Record Level:** language: en; institutionCode: CNC; collectionCode: Insects; basisOfRecord: Pinned Specimen
- **Type status:** Paratype. **Occurrence:** occurrenceDetails: http://janzen.sas.upenn.edu; catalogNumber: DHJPAR0017882; recordedBy: D.H. Janzen, W. Hallwachs & gusaneros; individualID: DHJPAR0017882; individualCount: 1; sex: male; lifeStage: adult; preparations: pinned; otherCatalogNumbers: ASTAR592-07, 99-SRNP-10081, BOLD:AAC2768; **Taxon:** scientificName: Vibrissina randyjonesi; phylum: Arthropoda; class: Insecta; order: Diptera; family: Tachinidae; genus: Vibrissina; specificEpithet: randyjonesi; scientificNameAuthorship: Fleming & Wood, 2016; **Location:** continent: Central America; country: Costa Rica; countryCode: CR; stateProvince: Guanacaste; county: Sector Santa Rosa; locality: Area de Conservación Guanacaste; verbatimLocality: Parcela John Sullivan; verbatimElevation: 280; verbatimLatitude: 10.8734; verbatimLongitude: -85.6265; verbatimCoordinateSystem: Decimal; decimalLatitude: 10.8734; decimalLongitude: -85.6265; **Identification:** identifiedBy: AJ Fleming; dateIdentified: 2016; **Event:** samplingProtocol: Reared from the larvae of the Argid sawfly, Durgoa mattogrossensis; verbatimEventDate: 21-Jun-2000; **Record Level:** language: en; institutionCode: CNC; collectionCode: Insects; basisOfRecord: Pinned Specimen
- **Type status:** Paratype. **Occurrence:** occurrenceDetails: http://janzen.sas.upenn.edu; catalogNumber: DHJPAR0053303; recordedBy: D.H. Janzen, W. Hallwachs & Johan Vargas; individualID: DHJPAR0053303; individualCount: 1; sex: female; lifeStage: adult; preparations: pinned; otherCatalogNumbers: ASHYM2657-13, 13-SRNP-18786, BOLD:AAC2768; **Taxon:** scientificName: Vibrissina randyjonesi; phylum: Arthropoda; class: Insecta; order: Diptera; family: Tachinidae; genus: Vibrissina; specificEpithet: randyjonesi; scientificNameAuthorship: Fleming & Wood, 2016; **Location:** continent: Central America; country: Costa Rica; countryCode: CR; stateProvince: Guanacaste; county: Sector Santa Rosa; locality: Area de Conservación Guanacaste; verbatimLocality: Area Administrativa; verbatimElevation: 295; verbatimLatitude: 10.8376; verbatimLongitude: -85.6187; verbatimCoordinateSystem: Decimal; decimalLatitude: 10.8376; decimalLongitude: -85.6187; **Identification:** identifiedBy: AJ Fleming; dateIdentified: 2016; **Event:** samplingProtocol: Reared from the larvae of the Argid sawfly, Durgoa mattogrossensis; **Record Level:** language: en; institutionCode: CNC; collectionCode: Insects; basisOfRecord: Pinned Specimen
- **Type status:** Paratype. **Occurrence:** occurrenceDetails: http://janzen.sas.upenn.edu; catalogNumber: DHJPAR0053334; recordedBy: D.H. Janzen, W. Hallwachs & Guillermo Pereira; individualID: DHJPAR0053334; individualCount: 1; sex: female; lifeStage: adult; preparations: pinned; otherCatalogNumbers: ASHYM2688-13, 13-SRNP-18759, BOLD:AAC2768; **Taxon:** scientificName: Vibrissina randyjonesi; phylum: Arthropoda; class: Insecta; order: Diptera; family: Tachinidae; genus: Vibrissina; specificEpithet: randyjonesi; scientificNameAuthorship: Fleming & Wood, 2016; **Location:** continent: Central America; country: Costa Rica; countryCode: CR; stateProvince: Guanacaste; county: Sector Santa Rosa; locality: Area de Conservación Guanacaste; verbatimLocality: Area Administrativa; verbatimElevation: 295; verbatimLatitude: 10.8376; verbatimLongitude: -85.6187; verbatimCoordinateSystem: Decimal; decimalLatitude: 10.8376; decimalLongitude: -85.6187; **Identification:** identifiedBy: AJ Fleming; dateIdentified: 2016; **Event:** samplingProtocol: Reared from the larvae of the Argid sawfly, Durgoa mattogrossensis; verbatimEventDate: 23-Aug-2013; **Record Level:** language: en; institutionCode: CNC; collectionCode: Insects; basisOfRecord: Pinned Specimen
- **Type status:** Paratype. **Occurrence:** occurrenceDetails: http://janzen.sas.upenn.edu; catalogNumber: DHJPAR0052603; recordedBy: D.H. Janzen, W. Hallwachs & Guillermo Pereira; individualID: DHJPAR0052603; individualCount: 1; sex: female; lifeStage: adult; preparations: pinned; otherCatalogNumbers: ASHYM1957-13, 13-SRNP-18733, BOLD:AAC2768; **Taxon:** scientificName: Vibrissina randyjonesi; phylum: Arthropoda; class: Insecta; order: Diptera; family: Tachinidae; genus: Vibrissina; specificEpithet: randyjonesi; scientificNameAuthorship: Fleming & Wood, 2016; **Location:** continent: Central America; country: Costa Rica; countryCode: CR; stateProvince: Guanacaste; county: Sector Santa Rosa; locality: Area de Conservación Guanacaste; verbatimLocality: Area Administrativa; verbatimElevation: 295; verbatimLatitude: 10.8376; verbatimLongitude: -85.6187; verbatimCoordinateSystem: Decimal; decimalLatitude: 10.8376; decimalLongitude: -85.6187; **Identification:** identifiedBy: AJ Fleming; dateIdentified: 2016; **Event:** samplingProtocol: Reared from the larvae of the Argid sawfly, Durgoa mattogrossensis; verbatimEventDate: 10-Aug-2013; **Record Level:** language: en; institutionCode: CNC; collectionCode: Insects; basisOfRecord: Pinned Specimen
- **Type status:** Paratype. **Occurrence:** occurrenceDetails: http://janzen.sas.upenn.edu; catalogNumber: DHJPAR0053205; recordedBy: D.H. Janzen, W. Hallwachs & Guillermo Pereira; individualID: DHJPAR0053205; individualCount: 1; sex: female; lifeStage: adult; preparations: pinned; otherCatalogNumbers: ASHYM2559-13, 13-SRNP-18757, BOLD:AAC2768; **Taxon:** scientificName: Vibrissina randyjonesi; phylum: Arthropoda; class: Insecta; order: Diptera; family: Tachinidae; genus: Vibrissina; specificEpithet: randyjonesi; scientificNameAuthorship: Fleming & Wood, 2016; **Location:** continent: Central America; country: Costa Rica; countryCode: CR; stateProvince: Guanacaste; county: Sector Santa Rosa; locality: Area de Conservación Guanacaste; verbatimLocality: Area Administrativa; verbatimElevation: 295; verbatimLatitude: 10.8376; verbatimLongitude: -85.6187; verbatimCoordinateSystem: Decimal; decimalLatitude: 10.8376; decimalLongitude: -85.6187; **Identification:** identifiedBy: AJ Fleming; dateIdentified: 2016; **Event:** samplingProtocol: Reared from the larvae of the Argid sawfly, Durgoa mattogrossensis; verbatimEventDate: 17-Oct-2013; **Record Level:** language: en; institutionCode: CNC; collectionCode: Insects; basisOfRecord: Pinned Specimen

#### Description

**Male.** (Fig. 8a, b, c). Length: 5–9mm. **Head** (Fig. 8b): parafrontal golden yellow tomentose; parafacial, face, gena, postgena and postorbit golden gold tomentose; antenna black-brown; arista reddish brown basally, black apically; gena 0.24X eye height; 3 reclinate orbital bristles; lower frontal bristle not reaching beyond lower level of pedicel; first flagellomere short of facial margin by 1.5X length of pedicel. **Thorax** (Fig. 8a, c): dorsum gold tomentose with 4 distinct dorsal vittae visible pre and postsuturally; scutellum almost entirely gold tomentose; anepisternum, anepimeron and katepisternum all gold tinged with silver around margins; 3 strong katepisternal bristles; 2 pairs of discal scutellar bristles; apical scutellars weak and strongly divergent. Legs: black on all segments. Wings: smoky gray, bearing 1–3 short bristles dorsally at the base of R4+5. **Abdomen** (Fig. 8a): ground color of abdomen black on ST1+2, T3, and T4; T5 of light orange ground color at apex; mid-dorsal depression on ST1+2 extending almost to margin of syntergite; anterior margins of T3, T4, and T5 bearing silver tomentum over 1/2 of tergal surface, with a slight gold tinge directly adjacent to the tergal margin; T5 with silver tomentum over 4/5 or more; T3 and T4 bearing 3 pairs of median discal bristles; T5 bearing 2 rows of discal bristles; ST1+2 and T3 with 1 pair of median marginal bristles; T4 and T5 bearing 1 complete row of marginal bristles. **Male terminalia** (Fig. 9): sternite 5 with two narrow rounded lobes, flanking a wide U-shaped median cleft; posterior lobes 0.67X length of anterior plate; inner margin covered in dense tomentum not appearing darker than surrounding cuticle; posterior lobes bearing short stout bristles throughout; anterior plate bare, wider than posterior lobes; cerci subtriangular in dorsal view, tapering rapidly to a slender apex, 1.37X as long as wide and separate along entire length; dorsally straight; in lateral view, with a slight dilation at apex, apically rounded and blade-like; surstylus, in lateral view, with straight ventral edge and curved dorsal edge, of cleaver-like appearance; postgonite parallel-sided and rounded at tip when viewed laterally, entire structure with a strong 90 degree bend along midopoint, giving it a rounded L-shaped appearance; anterobasal heel strongly rounded and ciliate; ventral sclerite of distiphallus linear along its entire length; membranous portion of distiphallus, when viewed laterally, displaying a small, upward-curved apical hook.

**Female** (Fig. 8d, e, f). Length: 5–7mm. As male with the exception of the following characters: **abdomen**: ventrolaterally flattened, bearing only one pair of discal bristles per segment; 1 pair of marginal bristles on ST1+2 and T3; mid-ventral portion of T3–T5 with a row of strong stout spines.

#### Diagnosis

*Vibrissina randyjonesi* **sp. n.** can be differentiated from its congeners by the combination of the following traits: parafacial and parafrontal all gold; tergite 5 of strong orange ground color at apex, this trait present in both males and females.

#### Etymology

*Vibrissina randyjonesi* **sp. n.** is dedicated to Mr. Randy Jones of Poland, Ohio, in recognition of his seminal support in acquiring the buildings in which the INBio national biodiversity inventory collections grew and thrived since their founding in 1989, and still do, now as part of Museo Nacional de Costa Rica.

#### Distribution

Costa Rica, ACG, Prov. Guanacaste, dry forest, between 280–300m.

#### Ecology

**Hosts**: reared 100+ times from larvae of *Durgoa mattogrossensis* (Argidae) feeding on the leaves of *Bauhinia ungulata* (Fabaceae).

### Vibrissina robertwellsi

Fleming & Wood sp. n.

urn:lsid:zoobank.org:act:EF73600B-8D7B-4BB4-8C65-7EE27FB80EDE

#### Materials

- **Type status:** Holotype. **Occurrence:** occurrenceDetails: http://janzen.sas.upenn.edu; catalogNumber: DHJPAR0017875; recordedBy: D.H. Janzen, W. Hallwachs & Roster Moraga; individualID: DHJPAR0017875; individualCount: 1; sex: male; lifeStage: adult; preparations: pinned; otherCatalogNumbers: ASTAR585-07, 00-SRNP-3100, BOLD:AAC2770; **Taxon:** scientificName: Vibrissina robertwellsi; phylum: Arthropoda; class: Insecta; order: Diptera; family: Tachinidae; genus: Vibrissina; specificEpithet: robertwellsi; scientificNameAuthorship: Fleming & Wood, 2016; **Location:** continent: Central America; country: Costa Rica; countryCode: CR; stateProvince: Guanacaste; county: Sector El Hacha; locality: Area de Conservación Guanacaste; verbatimLocality: Sendero Bejuquilla; verbatimElevation: 280; verbatimLatitude: 11.03; verbatimLongitude: -85.527; verbatimCoordinateSystem: Decimal; decimalLatitude: 11.03; decimalLongitude: -85.527; **Identification:** identifiedBy: AJ Fleming; dateIdentified: 2016; **Event:** samplingProtocol: Reared from the larvae of the Argid sawfly, Sericoceros vumirus; verbatimEventDate: 24-Jul-2000; **Record Level:** language: en; institutionCode: CNC; collectionCode: Insects; basisOfRecord: Pinned Specimen
- **Type status:** Paratype. **Occurrence:** occurrenceDetails: http://janzen.sas.upenn.edu; catalogNumber: DHJPAR0058285; recordedBy: D.H. Janzen, W. Hallwachs & gusaneros; individualID: DHJPAR0058285; individualCount: 1; sex: female; lifeStage: adult; preparations: pinned; otherCatalogNumbers: MHMYK11332-15, 89-SRNP-343, BOLD:AAC2770; **Taxon:** scientificName: Vibrissina robertwellsi; phylum: Arthropoda; class: Insecta; order: Diptera; family: Tachinidae; genus: Vibrissina; specificEpithet: robertwellsi; scientificNameAuthorship: Fleming & Wood, 2016; **Location:** continent: Central America; country: Costa Rica; countryCode: CR; stateProvince: Guanacaste; county: Sector Murcielago; locality: Area de Conservación Guanacaste; verbatimLocality: Camino Bahia Hachal; verbatimElevation: 5; verbatimLatitude: 10.9334; verbatimLongitude: -85.7291; verbatimCoordinateSystem: Decimal; decimalLatitude: 10.9334; decimalLongitude: -85.7291; **Identification:** identifiedBy: AJ Fleming; dateIdentified: 2016; **Event:** samplingProtocol: Reared from the larvae of the Argid sawfly, Sericoceros mexicanus; verbatimEventDate: 18-Jul-1989; **Record Level:** language: en; institutionCode: CNC; collectionCode: Insects; basisOfRecord: Pinned Specimen
- **Type status:** Paratype. **Occurrence:** occurrenceDetails: http://janzen.sas.upenn.edu; catalogNumber: DHJPAR0058284; recordedBy: D.H. Janzen, W. Hallwachs & gusaneros; individualID: DHJPAR0058284; individualCount: 1; sex: female; lifeStage: adult; preparations: pinned; otherCatalogNumbers: MHMYK11331-15, 86-SRNP-527, BOLD:AAC2770; **Taxon:** scientificName: Vibrissina robertwellsi; phylum: Arthropoda; class: Insecta; order: Diptera; family: Tachinidae; genus: Vibrissina; specificEpithet: robertwellsi; scientificNameAuthorship: Fleming & Wood, 2016; **Location:** continent: Central America; country: Costa Rica; countryCode: CR; stateProvince: Guanacaste; county: Sector Santa Rosa; locality: Area de Conservación Guanacaste; verbatimLocality: Area Administrativa; verbatimElevation: 295; verbatimLatitude: 10.8376; verbatimLongitude: -85.6187; verbatimCoordinateSystem: Decimal; decimalLatitude: 10.8376; decimalLongitude: -85.6187; **Identification:** identifiedBy: AJ Fleming; dateIdentified: 2016; **Event:** samplingProtocol: Reared from the larvae of the Argid sawfly, Sericoceros vumirus; verbatimEventDate: 25-Nov-1986; **Record Level:** language: en; institutionCode: CNC; collectionCode: Insects; basisOfRecord: Pinned Specimen
- **Type status:** Paratype. **Occurrence:** occurrenceDetails: http://janzen.sas.upenn.edu; catalogNumber: DHJPAR0017808; recordedBy: D.H. Janzen, W. Hallwachs & Roster Moraga; individualID: DHJPAR0017808; individualCount: 1; sex: male; lifeStage: adult; preparations: pinned; otherCatalogNumbers: ASTAR519-07, 00-SRNP-3104, BOLD:AAC2770; **Taxon:** scientificName: Vibrissina robertwellsi; phylum: Arthropoda; class: Insecta; order: Diptera; family: Tachinidae; genus: Vibrissina; specificEpithet: robertwellsi; scientificNameAuthorship: Fleming & Wood, 2016; **Location:** continent: Central America; country: Costa Rica; countryCode: CR; stateProvince: Guanacaste; county: Sector El Hacha; locality: Area de Conservación Guanacaste; verbatimLocality: Sendero Bejuquilla; verbatimElevation: 280; verbatimLatitude: 11.03; verbatimLongitude: -85.527; verbatimCoordinateSystem: Decimal; decimalLatitude: 11.03; decimalLongitude: -85.527; **Identification:** identifiedBy: AJ Fleming; dateIdentified: 2016; **Event:** samplingProtocol: Reared from the larvae of the Argid sawfly, Sericoceros vumirus; verbatimEventDate: 25-Jul-2000; **Record Level:** language: en; institutionCode: CNC; collectionCode: Insects; basisOfRecord: Pinned Specimen

#### Description

**Male** (Fig. 10a, b, c). Length: 8mm. **Head** (Fig. 10b): parafrontal and postorbit golden-yellow tomentose; parafacial, face, gena, and postgena silver tomentose; antenna black-brown; arista reddish brown basally, black apically; gena 0.25X eye height; 2 reclinate orbital bristles; frontal bristles not reaching below lower margin of pedicel; first flagellomere short of facial margin by 2X length of pedicel. **Thorax** (Fig. 10a, c): dorsum pale gold tomentose with 4 distinct dorsal vittae visible presuturally, postsuturally still distinct but widened and slightly smudging together; scutellum gold tomentose over almost its entirety; 1 short pair of discal scutellar bristles; apical scutellars weak and strongly divergent; 3 katepisternal bristles, ventral katepisternal bristle appearing greatly reduced compared to other 2; anepisternum slightly gold tomentose, anepimeron very lightly gold tomentose along anterior 1/2 only; katepisternum silver-gray tomentose with a gold tinge confined to dorsal edge. Legs: femora dark brown black, tibiae and tarsi with a light brown-reddish tinge under certain angles of light. Wings: smoky gray, bearing 2 short setulae at the base of R4+5. **Abdomen** (Fig. 10a): ground color of abdomen light brown laterally, black dorsally; ST1+2 all black, mid-dorsal depression extending to margin of syntergite; anterior margins of T3, T4 and T5 bearing gold tomentum over approximately ½ of tergal surface; ST1+2 bearing 1 pair of median marginal bristles; T3, T4 and T5 each bearing complete rows of marginal bristles; T3 and T4 with 3 pairs of median discal bristles; T5 bearing 1 row of discal bristles. **Male terminalia** (Fig. 11): sternite 5 with two broad rounded lobes, flanking a wide U-shaped median cleft; posterior lobes 0.62X length of anterior plate; inner margin covered in dense tomentum extending to upper margin of lobe, making it appear darker than surrounding cuticle; posterior lobes bearing short stout bristles throughout; anterior plate bare, wider than posterior lobes, with 3–5 small unsclerotized dots along midline; cerci triangular in dorsal view, tapering rapidly, 1.69X as long as wide, separate along entire length; cerci in lateral view dorsally slightly convex with a very slight upturn at apex, apically rounded and blade-like; surstylus, in lateral view, with ventral edge straight and with a curved dorsal edge, of a leaf-like appearance; postgonite parallel-sided and clubbed at tip when viewed laterally, entire structure with a strong curve along midopoint, giving it a rounded C shaped appearance; anterobasal heel strongly rounded; ventral sclerite of distiphallus slightly curved at apex; membranous portion of distiphallus, when viewed laterally, displaying a small downward-curved apical hook.

**Female** (Fig. 10d, e, f). Length: 5mm. As male except for the following traits: **head**: parafrontal, postorbit and parafacial gold tomentose; gena and postgena silver tomentose; antenna brown; gena 0.18X eye height. **Thorax**: dorsum densely gold tomentose with 4 distinct dorsal vittae visible presuturally; postsuturally vittae still separate and distinct, covering slightly over 1/2 of postsutural scutum; scutellum gold tomentose over posterior 3/4 or more; 3 strong katepisternal bristles, with ventral katepisternal bristle appearing greatly reduced compared to the other 2, but still well developed; anepisternum, anepimeron and katepisternum very slightly gold tomentose along dorsal margin. Legs: reddish brown on all segments. Wings: smoky gray, bearing 2–3 short setulae at the base of R4+5. **Abdomen**: ground color dark brown-black overall except for T5, whose ground color is a prominently yellow-orange. ST1+2 all black, mid-dorsal depression extending to margin of syntergite; anterior margin of T3, T4 and T5 bearing gold-gray tomentum over more than 1/2 of tergal surface; ST1+2 bearing 1 pair of median marginal bristles; T3, T4 and T5 each bearing rows of marginal bristles; T3 with 1 pair of median discal bristles; T3, T4, and T5 ventrolaterally flattened; mid-ventral portion of T3–T5 with a row of strong stout spines.

#### Diagnosis

*Vibrissina robertwellsi* **sp. n.** can be differentiated from its congeners by the combination of the following traits: parafacial silver and parafrontal gold; tergite 5 of strong orange ground color over more than 50% of tergite, a trait most prominent in females.

#### Etymology

*Vibrissina robertwellsi* **sp. n.** is dedicated to Mr. Robert Wells of San José, Costa Rica in recognition of his legal manoeuvering and contract management for INBio’s purchase of the lands on which the national biodiversity inventory collections have grown and thrived since 1989, and which have now been donated to the Museo Nacional de Costa Rica.

#### Distribution

Costa Rica, ACG, Prov. Guanacaste, dry forest, in a dry-rain lowland intergrade at 5–295m.

#### Ecology

**Hosts**: reared on six occasions from larvae of the sawfly *Sericoceros vumirus* Smith (Argidae), feeding on the leaves of *Ipomoea nil* (L.) (Convolvulaceae), *Coccoloba uvifera* L. (Polygonaceae), *Lonchocarpus guatemalensis* Benth. and *Lonchocarpus minimiflorus* Donn. Sm. (Fabaceae).

## Analysis

**Figure 12.**
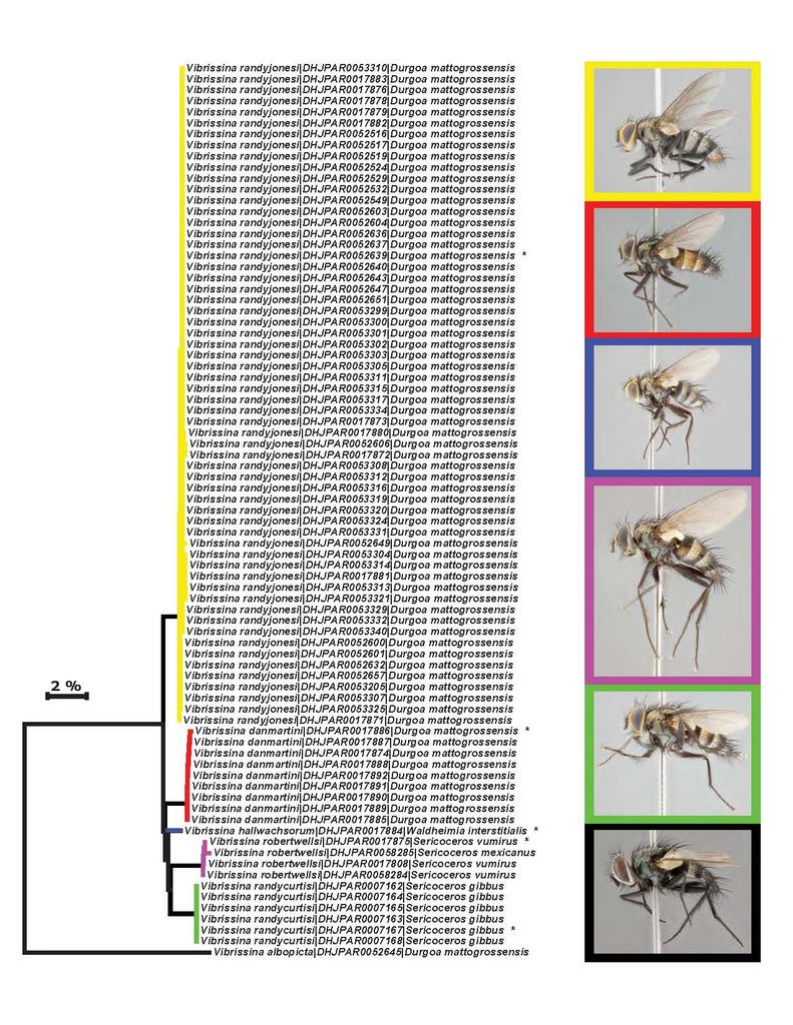
Neighbor-Joining displaying the inter- and intra-specific variation within the DNA barcode region for the 6 species of *Vibrissina* Rondani reared from ACG. Tip labels include the species name|specimen accession number|host species; the holotype of each species is indicated with an asterisk. Habitus photographs in lateral view are presented for the holotypes of the new species. An ACG-reared voucher specimen is presented for *V. albopicta*.

A neighbor-joining (NJ) (Saitou and Nei 1987) tree based on Kimura 2-parameter was used to visually demonstrate the variation present within and between each species in the DNA barcode locus and is presented in Fig. 12. The variation illustrated is based on the evolutionary distances computed using the Tamura-Nei (TN) method (Tamura and Nei 1993). The TN method was selected due to the lowest BIC (Bayesian Information Criterion) scores of the Maximum Likelihood fits of 24 different nucleotide substitution models run in MEGA6 (Tamura et al. 2013). Interested readers can consult the Barcode of Life Data System (BOLD) (Ratnasingham and Hebert 2007) for all information associated with each sequence (including GenBank accessions) derived from each individual specimen using the DOI dx.doi.org/10.5883/DS-ASVIBRI.

## Supplementary Material

XML Treatment for Vibrissina

XML Treatment for Vibrissina albopicta

XML Treatment for Vibrissina danmartini

XML Treatment for Vibrissina hallwachsorum

XML Treatment for Vibrissina randycurtisi

XML Treatment for Vibrissina randyjonesi

XML Treatment for Vibrissina robertwellsi

## Figures and Tables

**Figure 1a.**
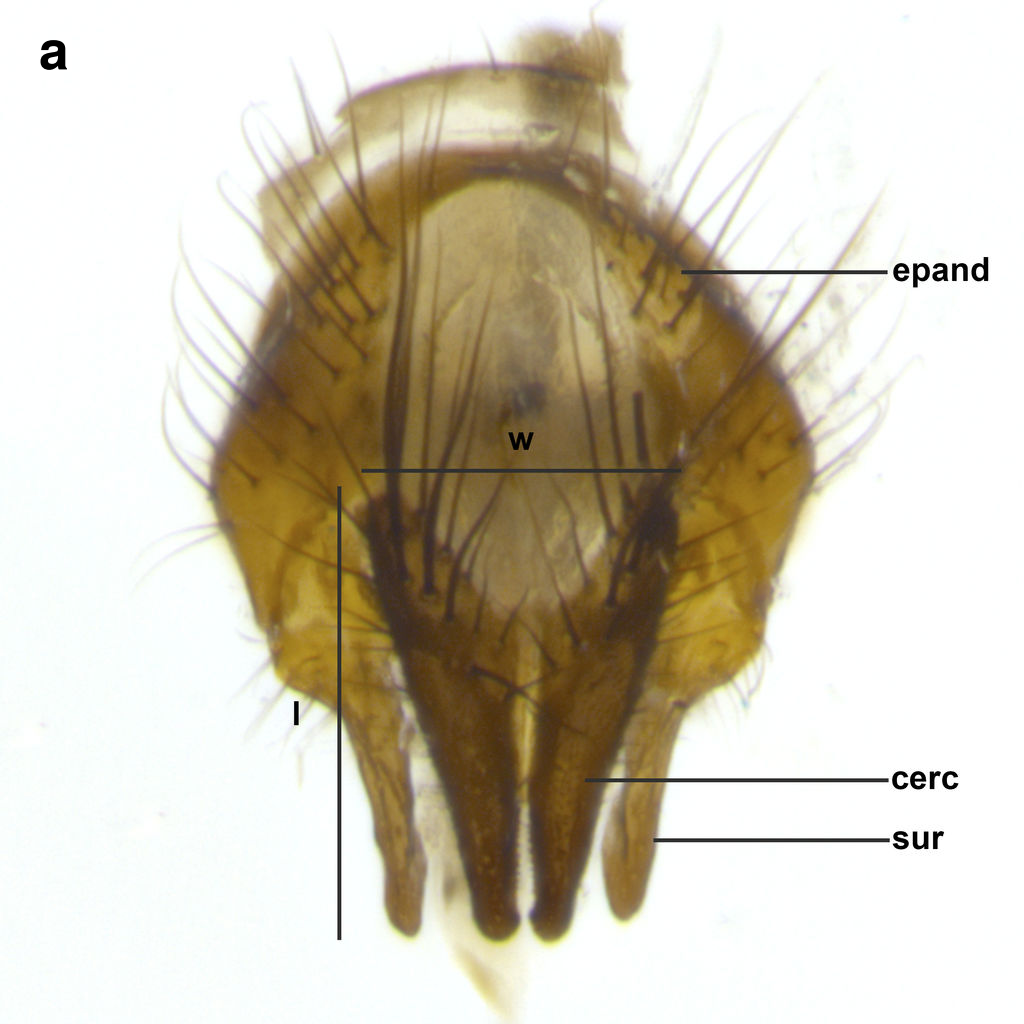
lateral view of terminalia of *V. danmartini* **sp. n.**

**Figure 1b.**
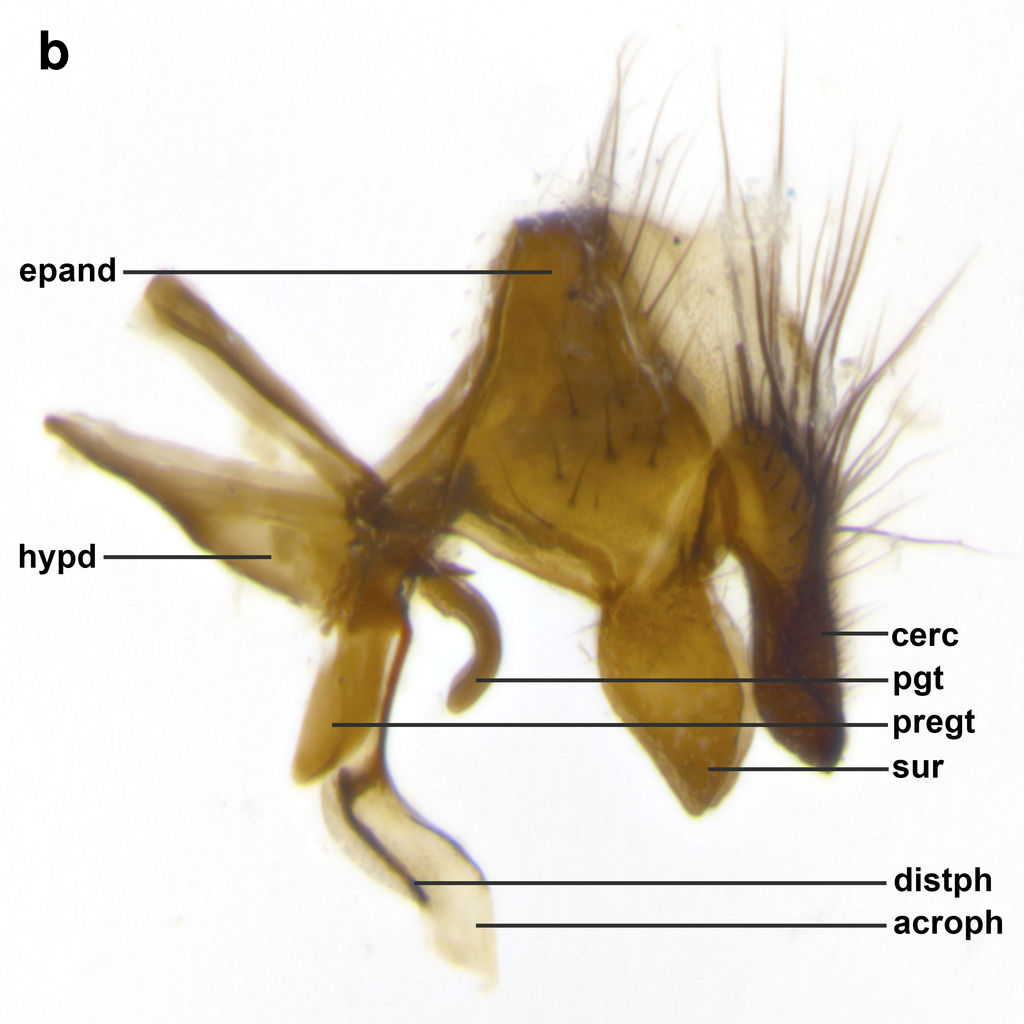
dorsal view of terminalia of *V. danmartini* **sp. n.**

**Figure 1c.**
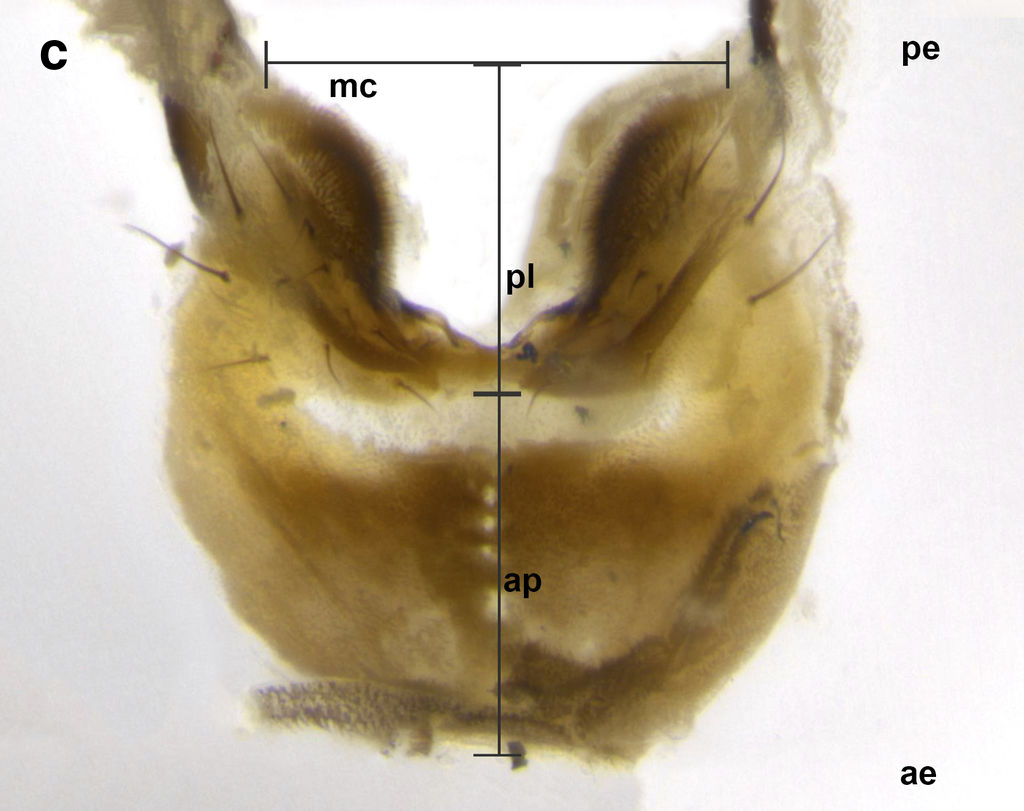
ventral view of sternite 5 of *V. robertwellsii* **sp. n.**

**Figure 2a.**
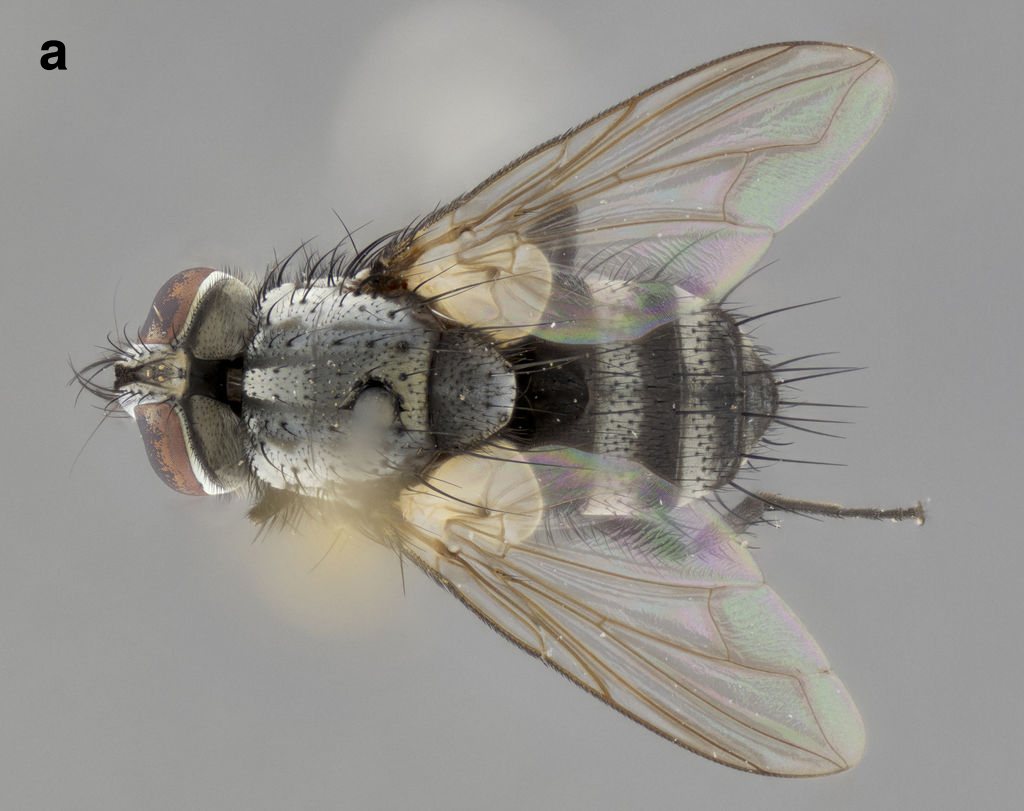
habitus in dorsal view

**Figure 2b.**
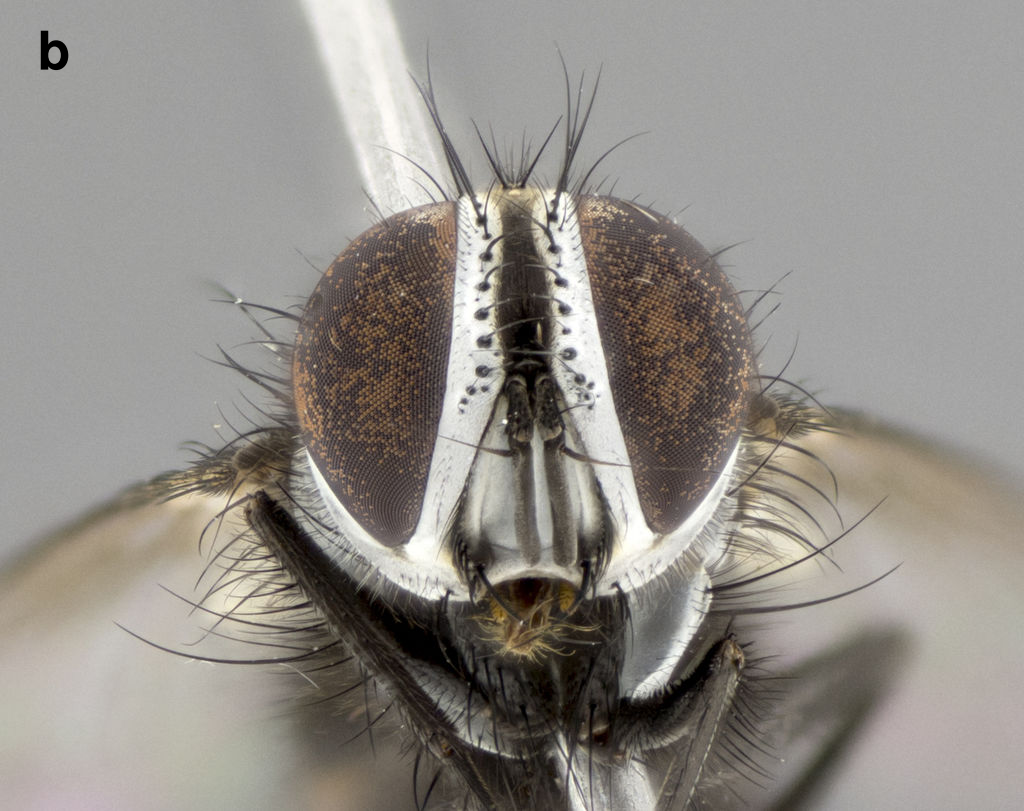
head in frontal view

**Figure 2c.**
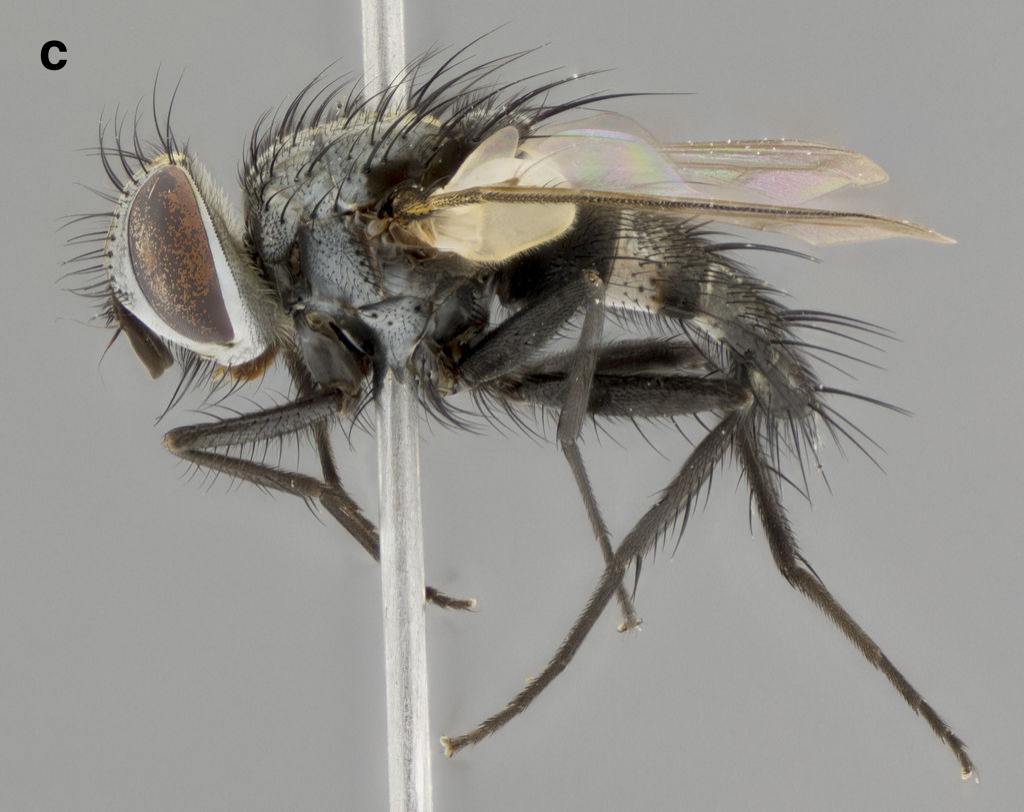
habitus in lateral view

**Figure 3a.**
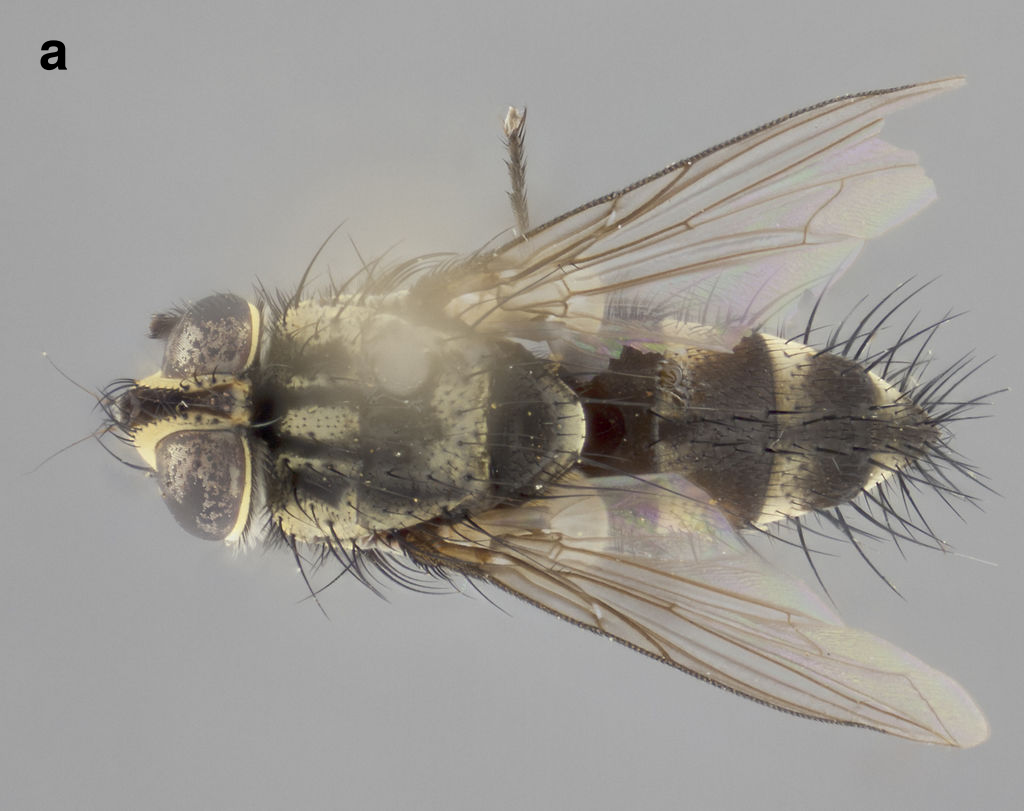
habitus in dorsal view

**Figure 3b.**
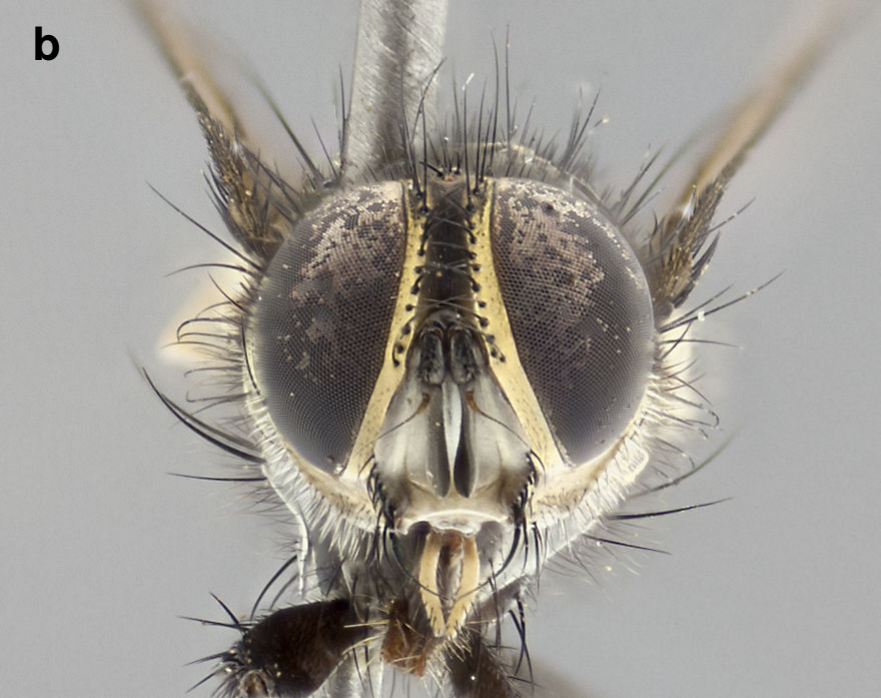
head in frontal view

**Figure 3c.**
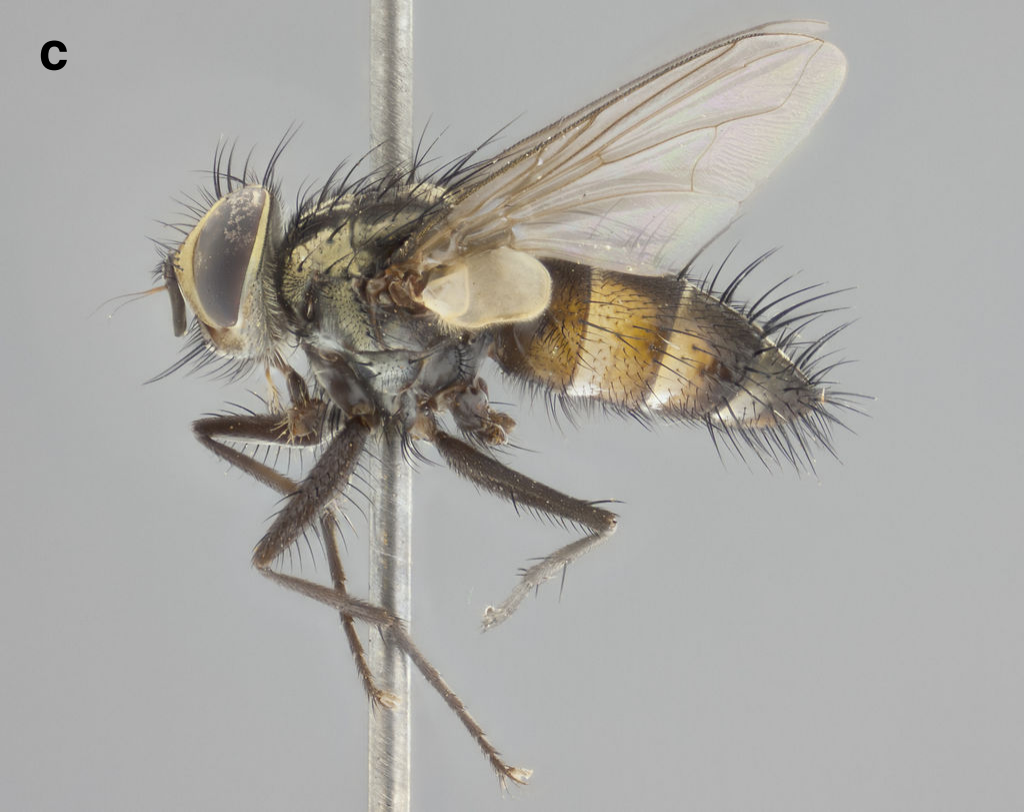
habitus in lateral view

**Figure 3d.**
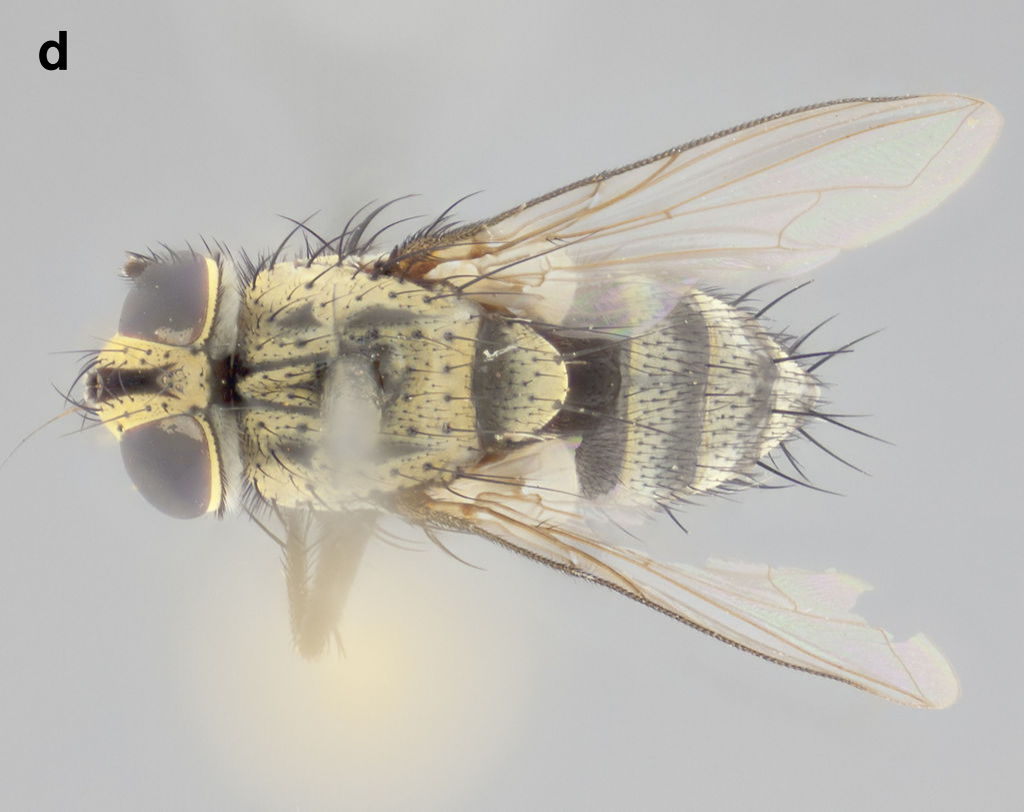
habitus in dorsal view

**Figure 3e.**
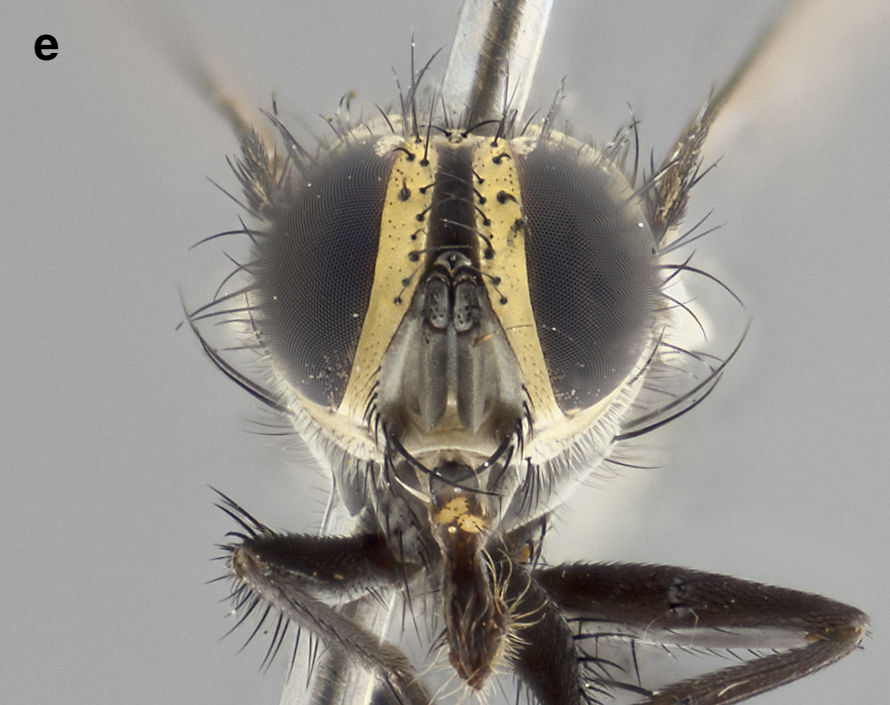
head in frontal view

**Figure 3f.**
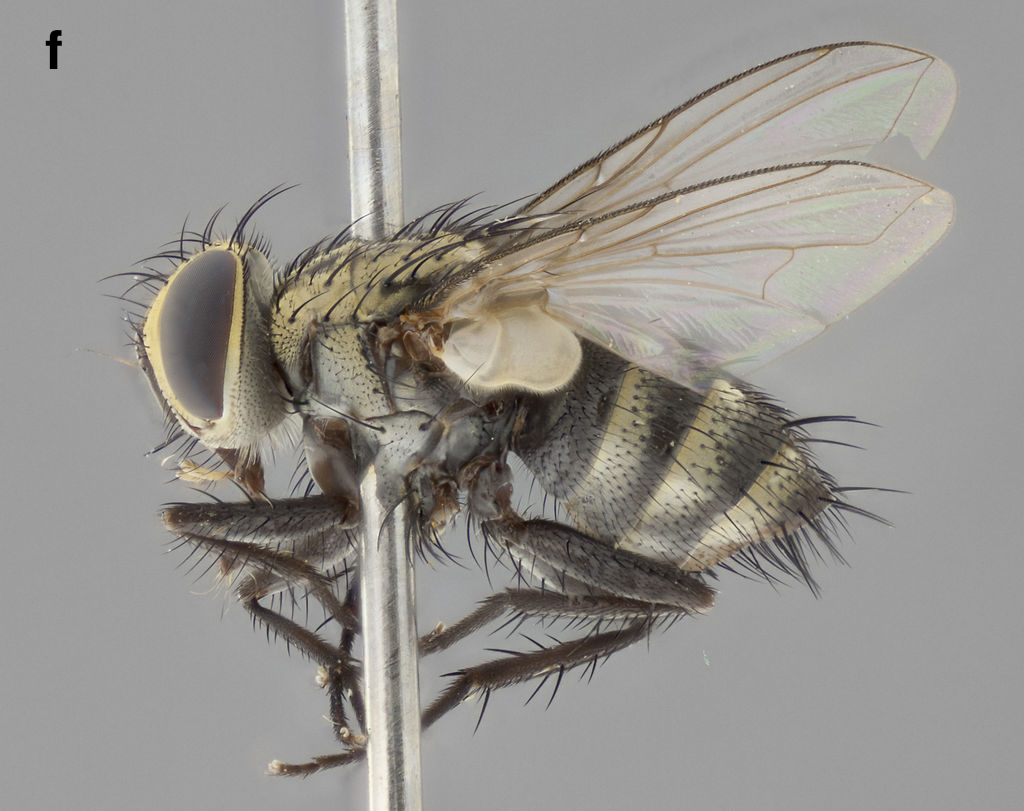
habitus in lateral view

**Figure 4a.**
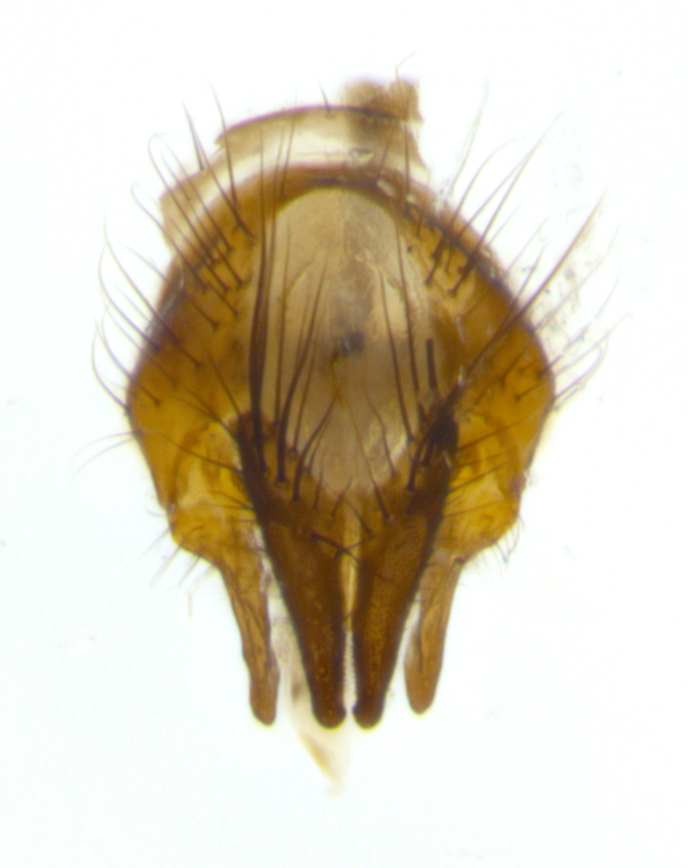
cerci and surstyli in dorsal view

**Figure 4b.**
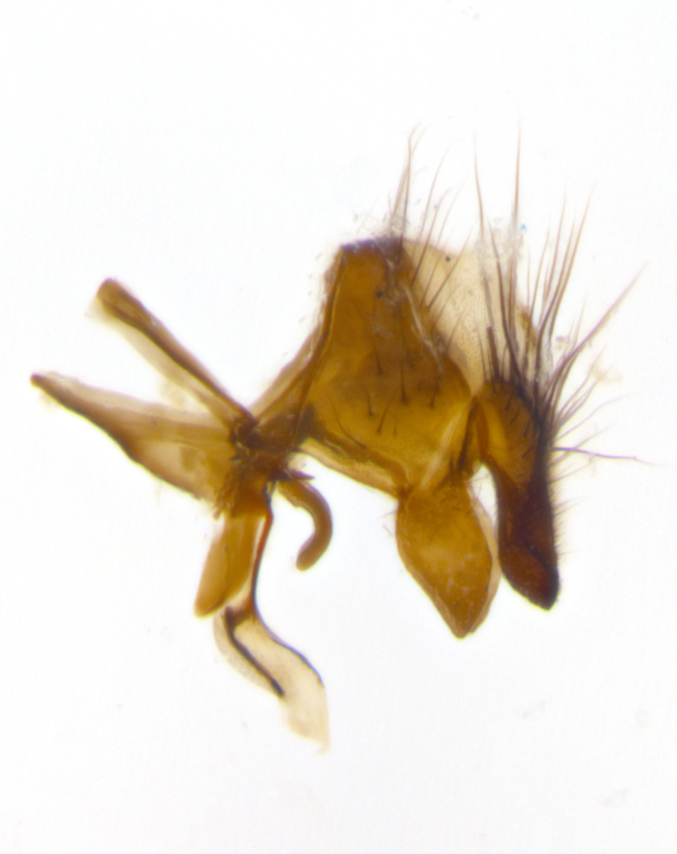
terminalia in lateral view

**Figure 4c.**
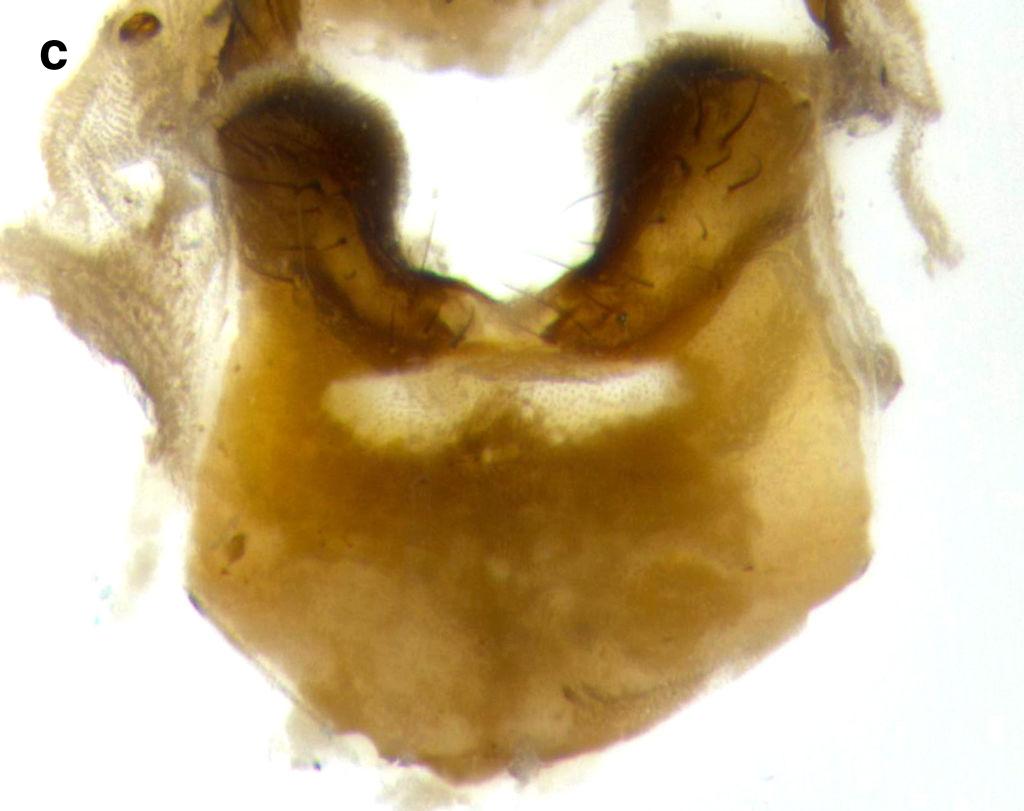
sternite 5 in ventral view

**Figure 5a.**
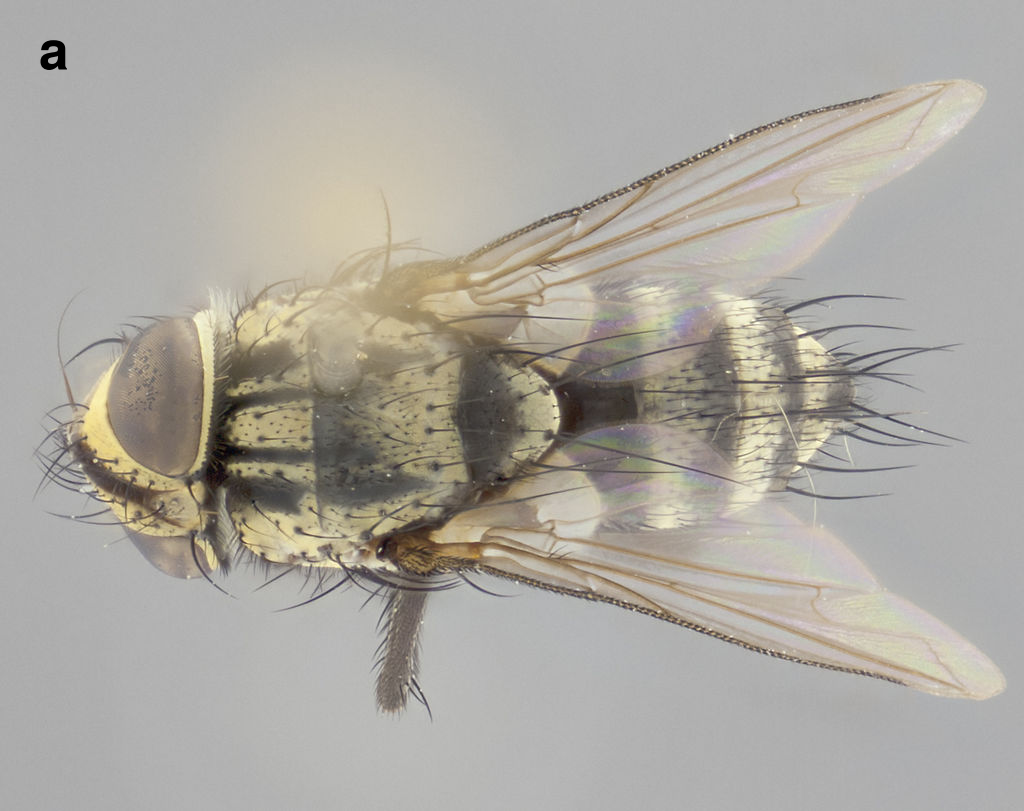
habitus in dorsal view

**Figure 5b.**
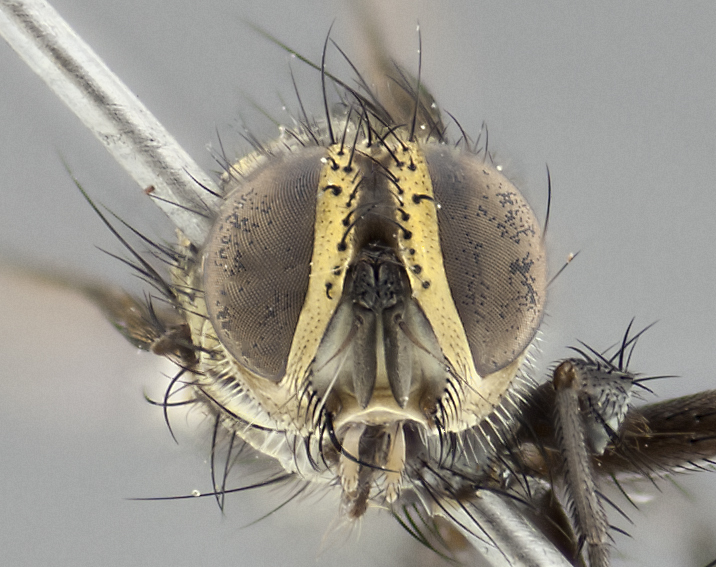
head in frontal view

**Figure 5c.**
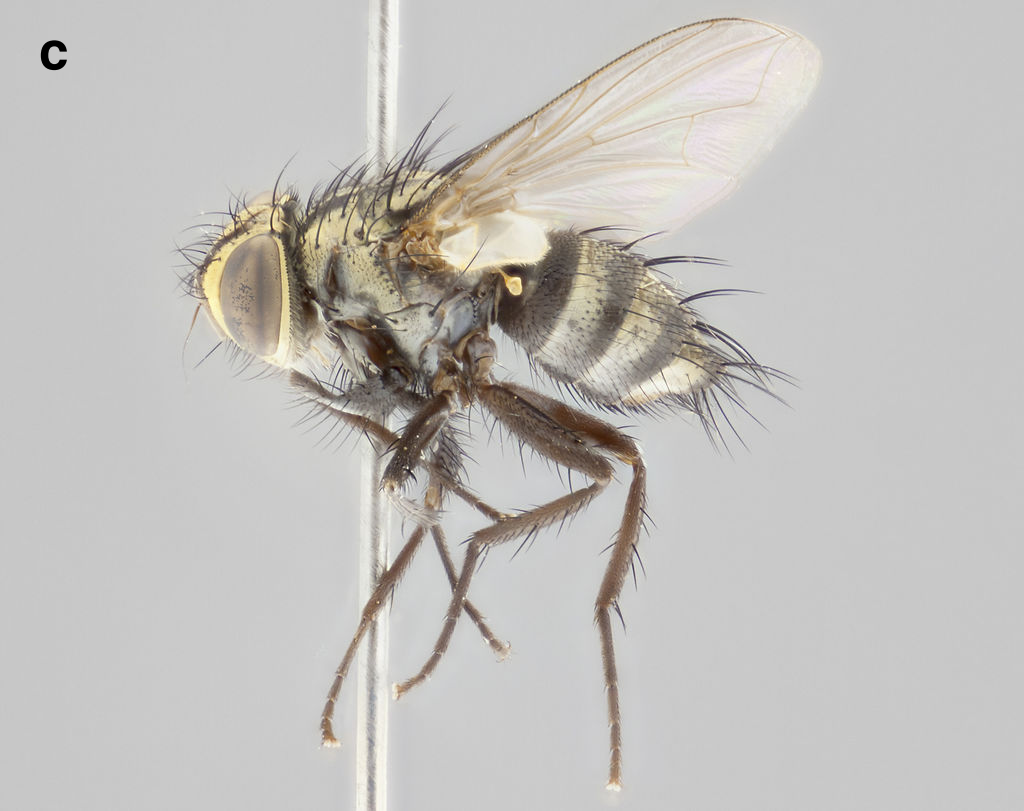
habitus in lateral view

**Figure 6a.**
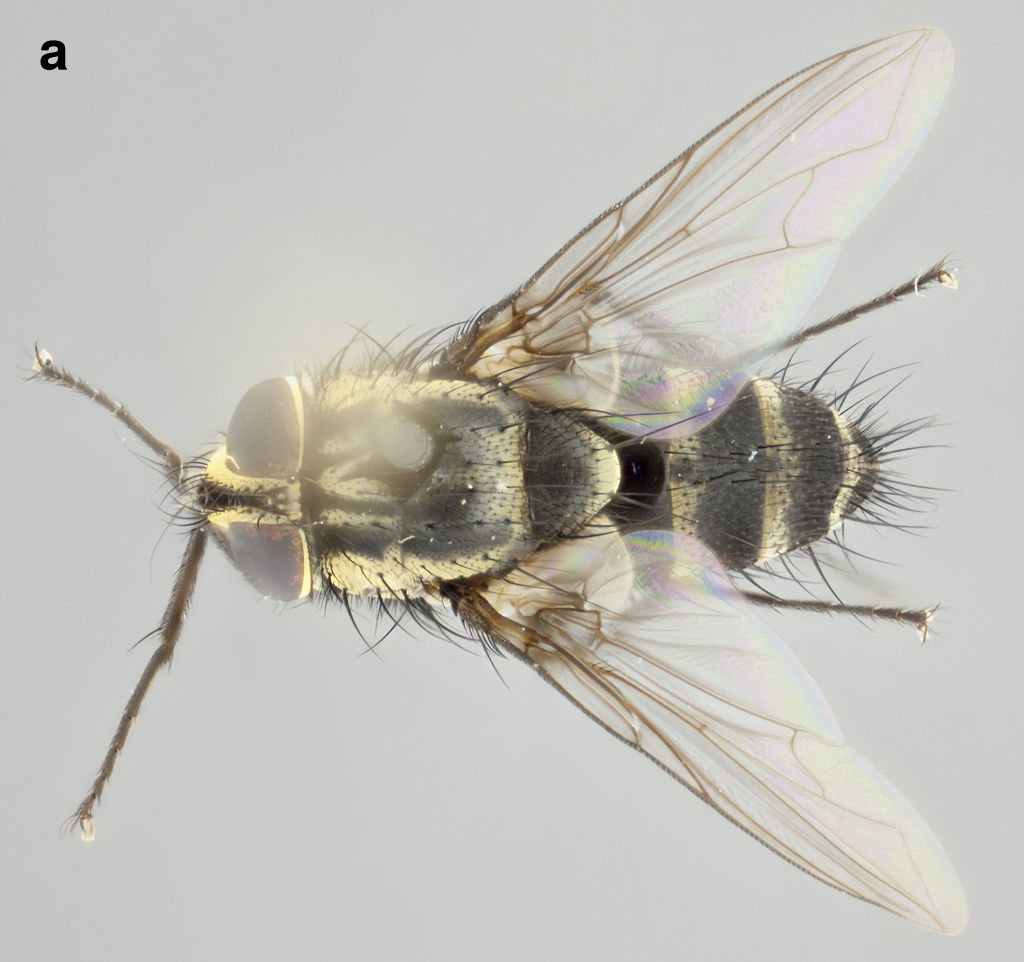
habitus in dorsal view

**Figure 6b.**
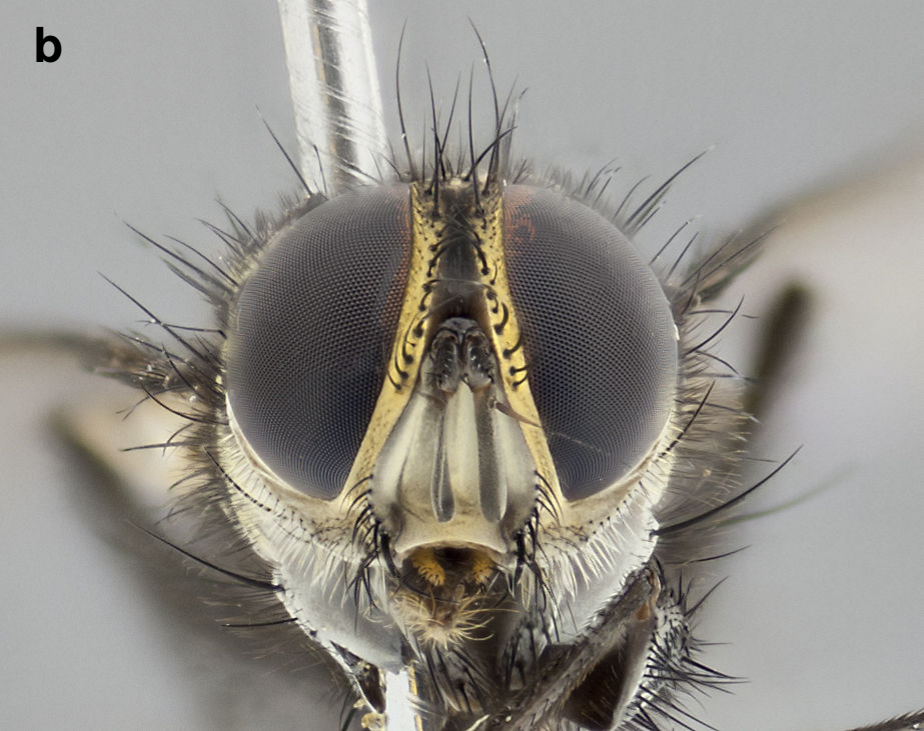
head in frontal view

**Figure 6c.**
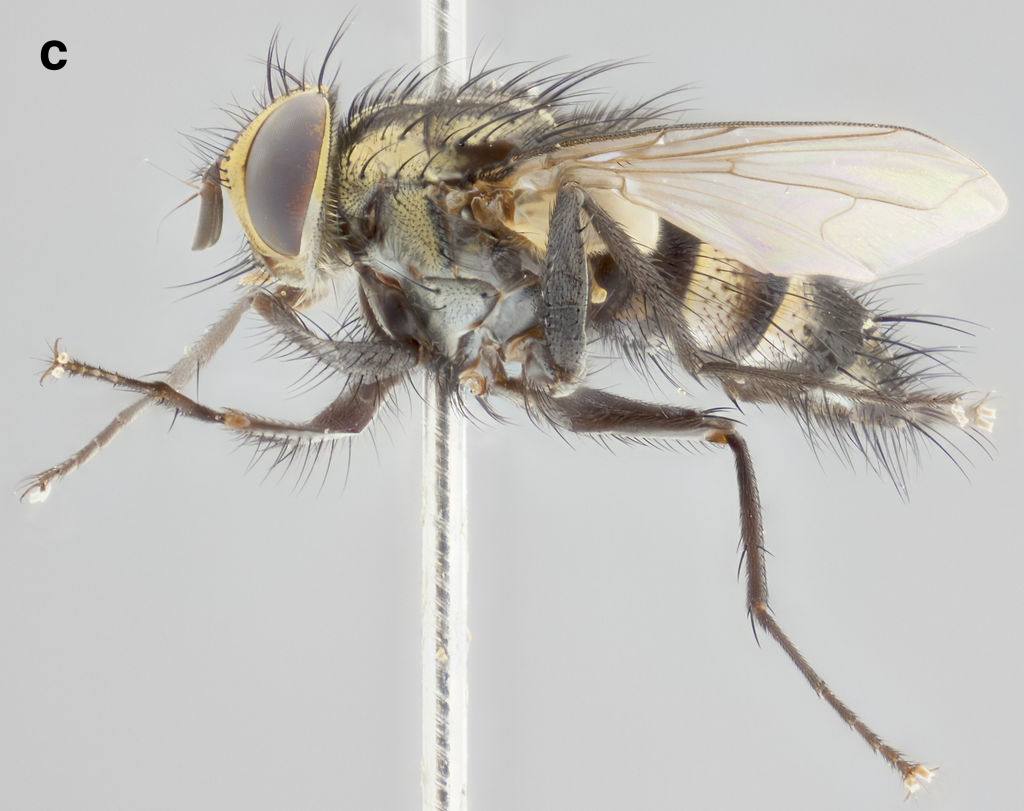
habitus in lateral view

**Figure 6d.**
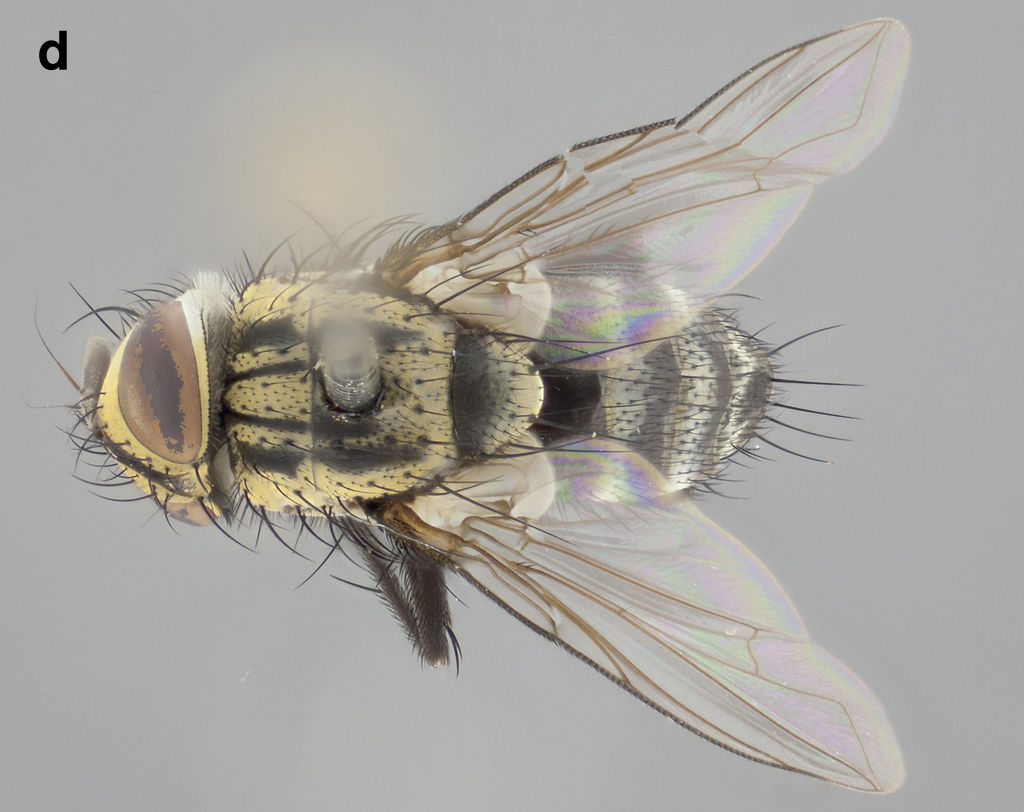
habitus in dorsal view

**Figure 6e.**
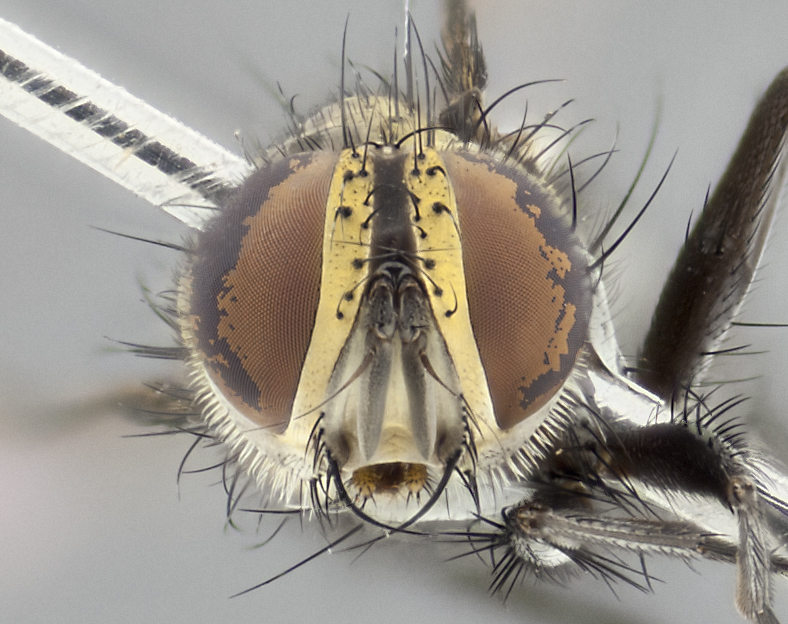
head in frontal view

**Figure 6f.**
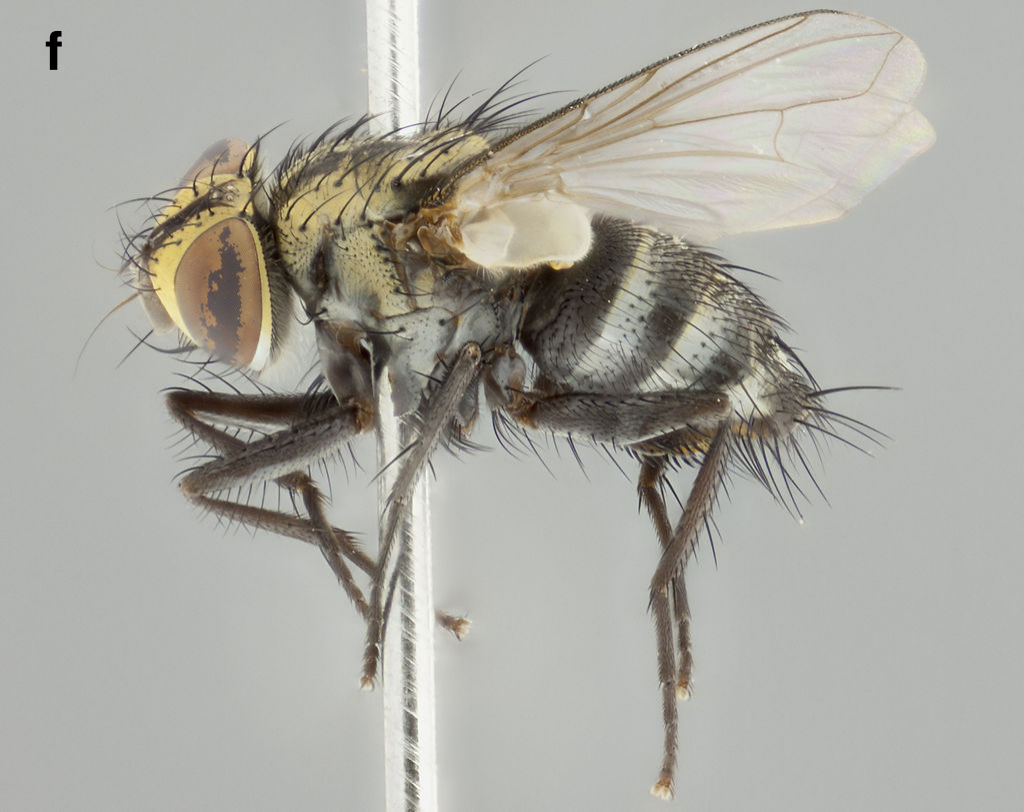
habitus in lateral view

**Figure 7a.**
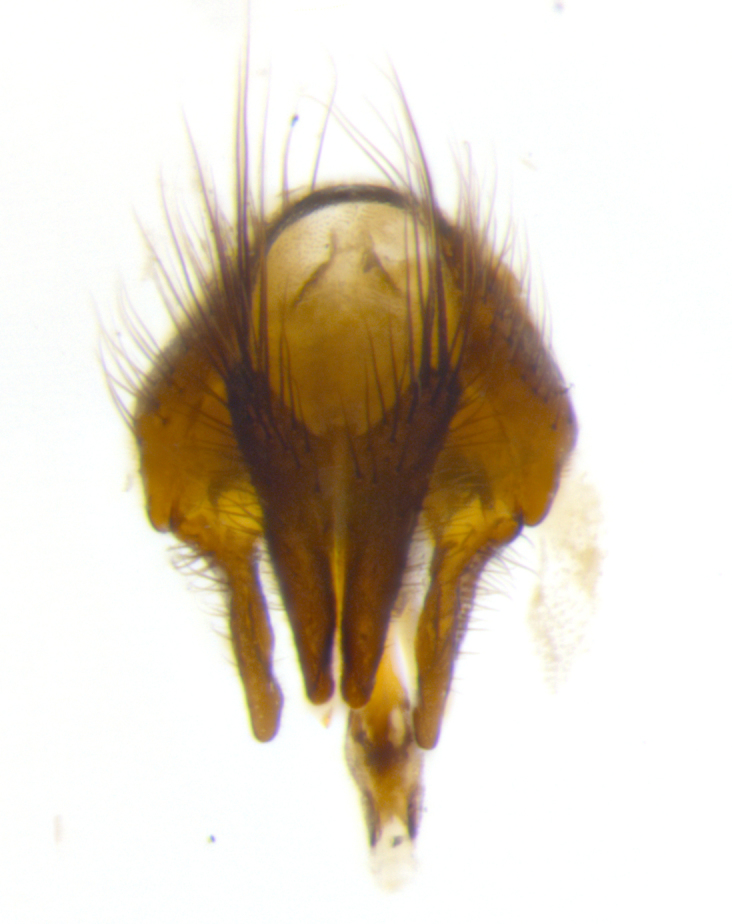
terminalia in dorsal view

**Figure 7b.**
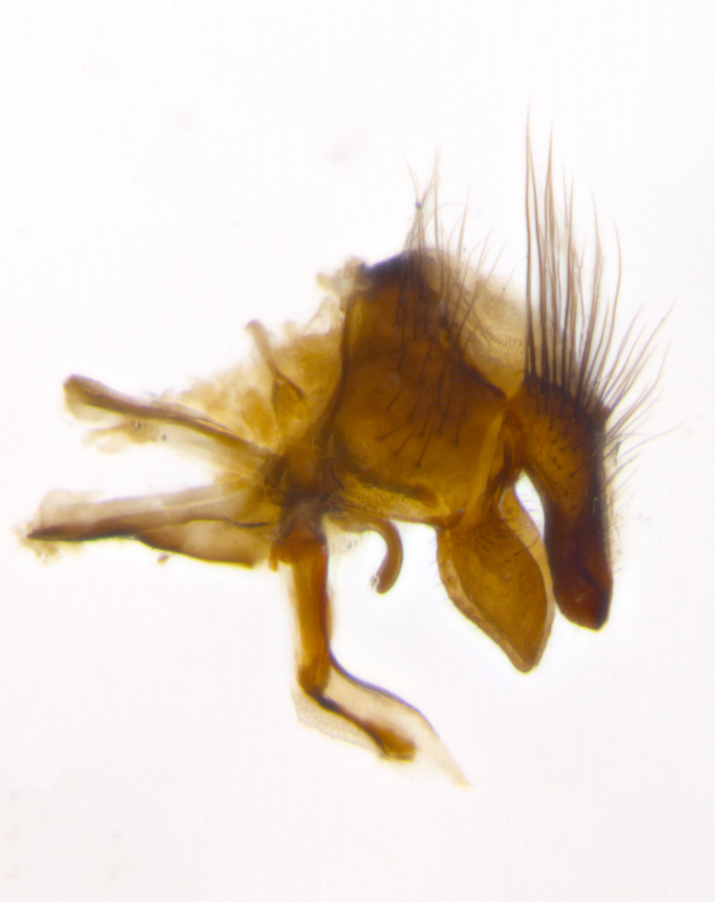
terminalia in lateral view

**Figure 7c.**
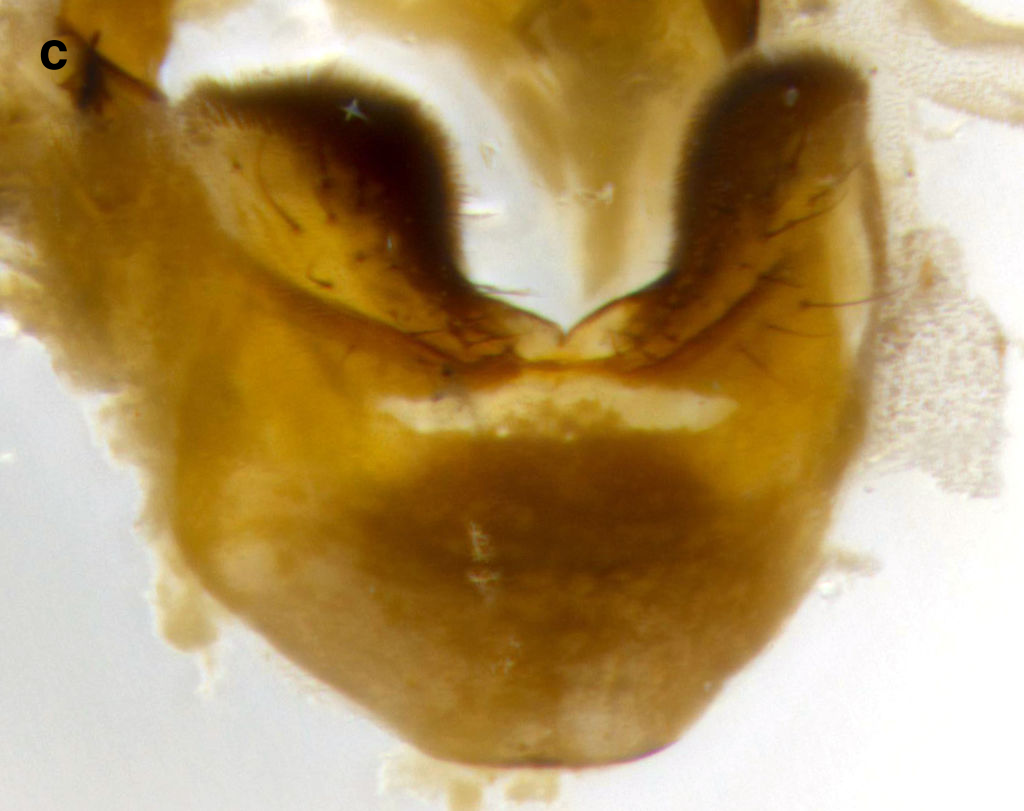
sternite 5 in ventral view

**Figure 8a.**
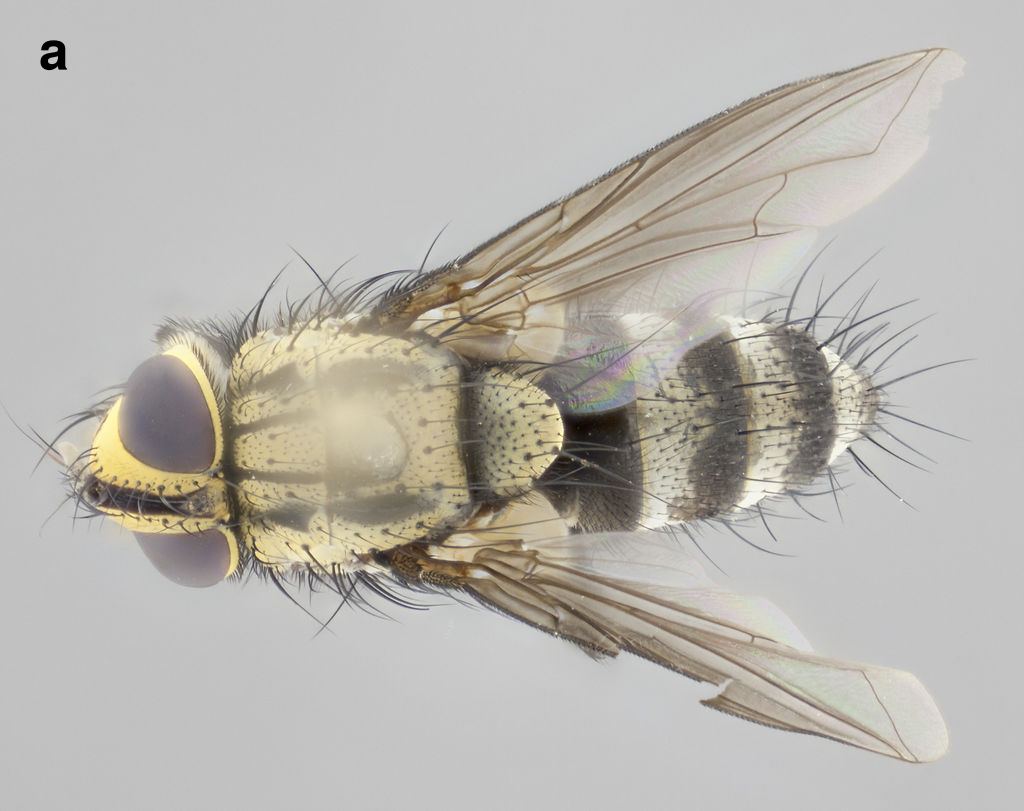
habitus in dorsal view

**Figure 8b.**
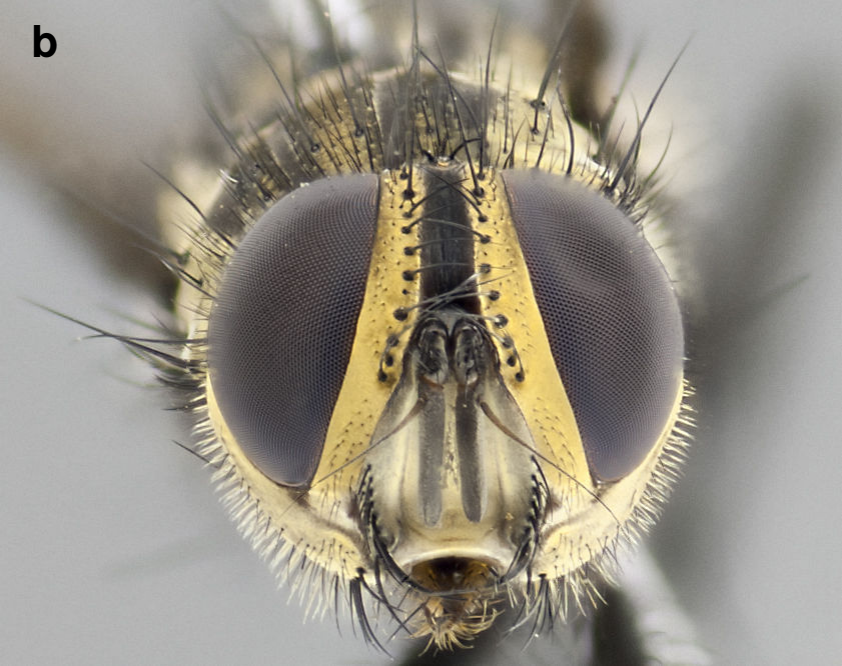
head in frontal view

**Figure 8c.**
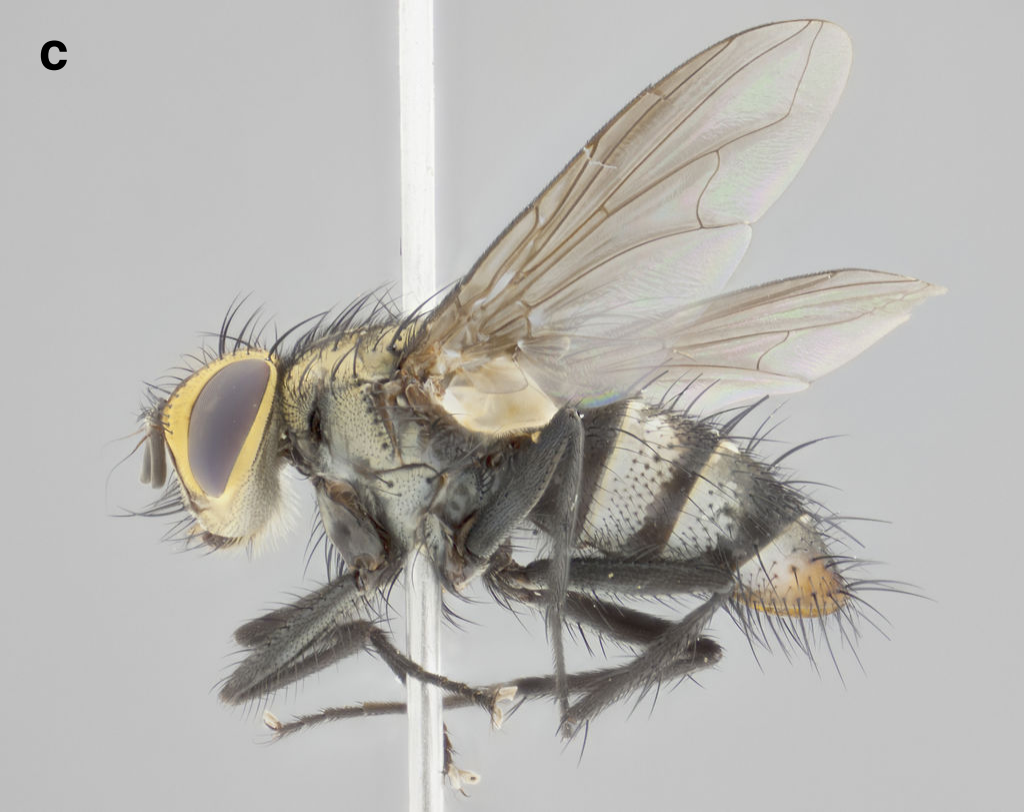
habitus in lateral view

**Figure 8d.**
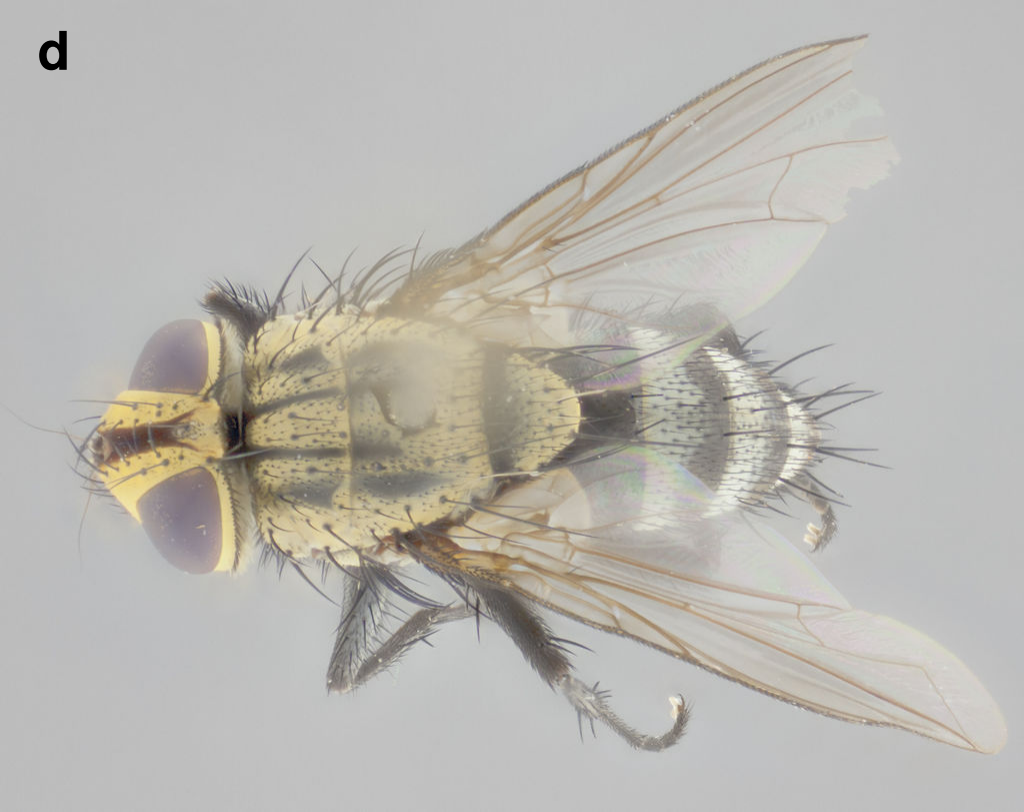
habitus in dorsal view

**Figure 8e.**
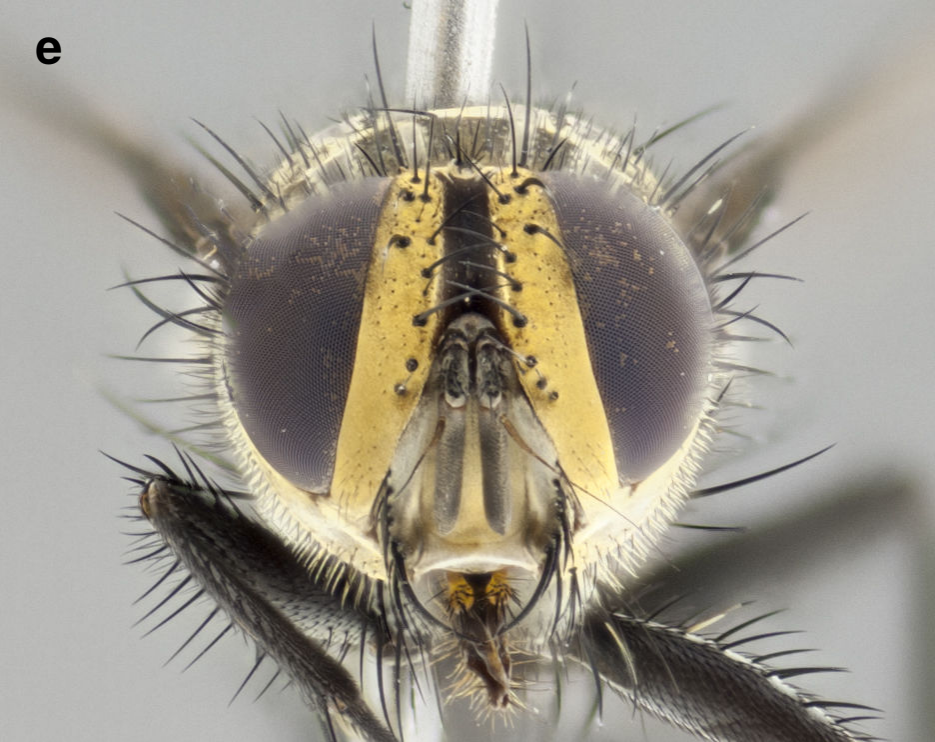
head in frontal view

**Figure 8f.**
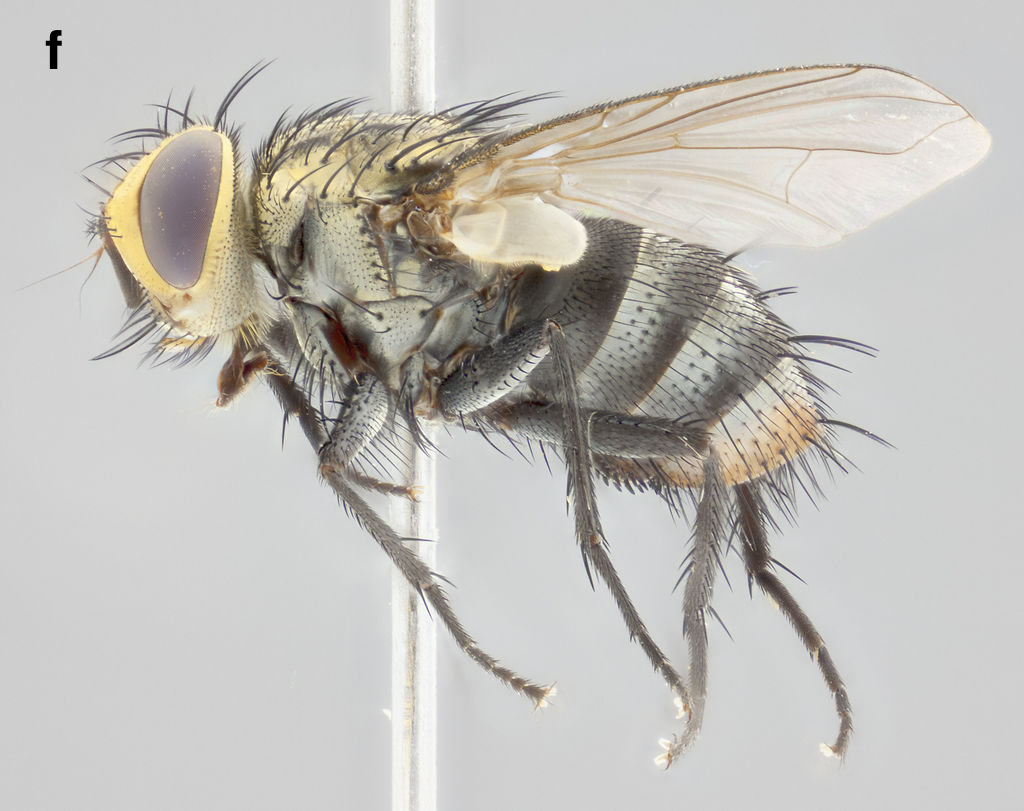
habitus in lateral view

**Figure 9a.**
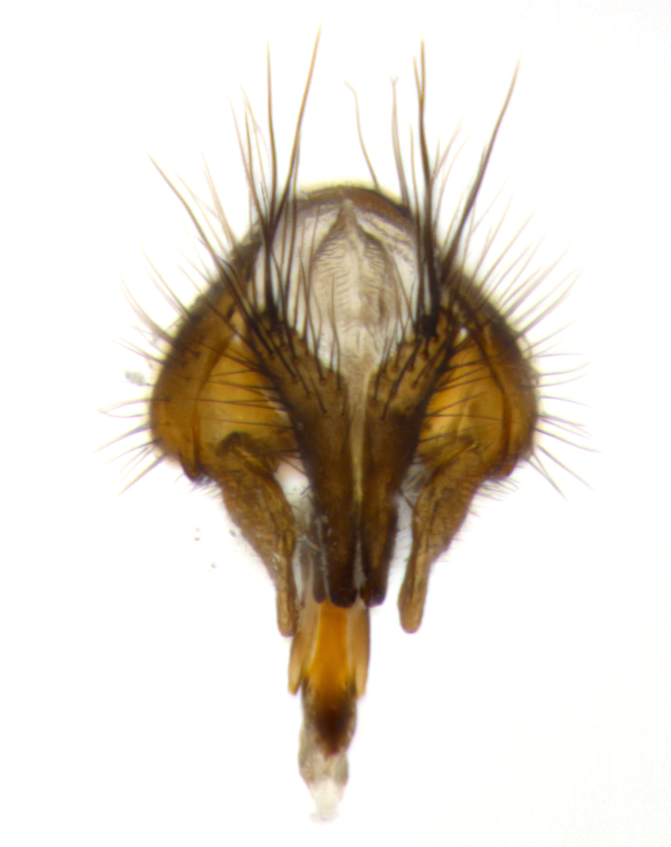
terminalia in dorsal view

**Figure 9b.**
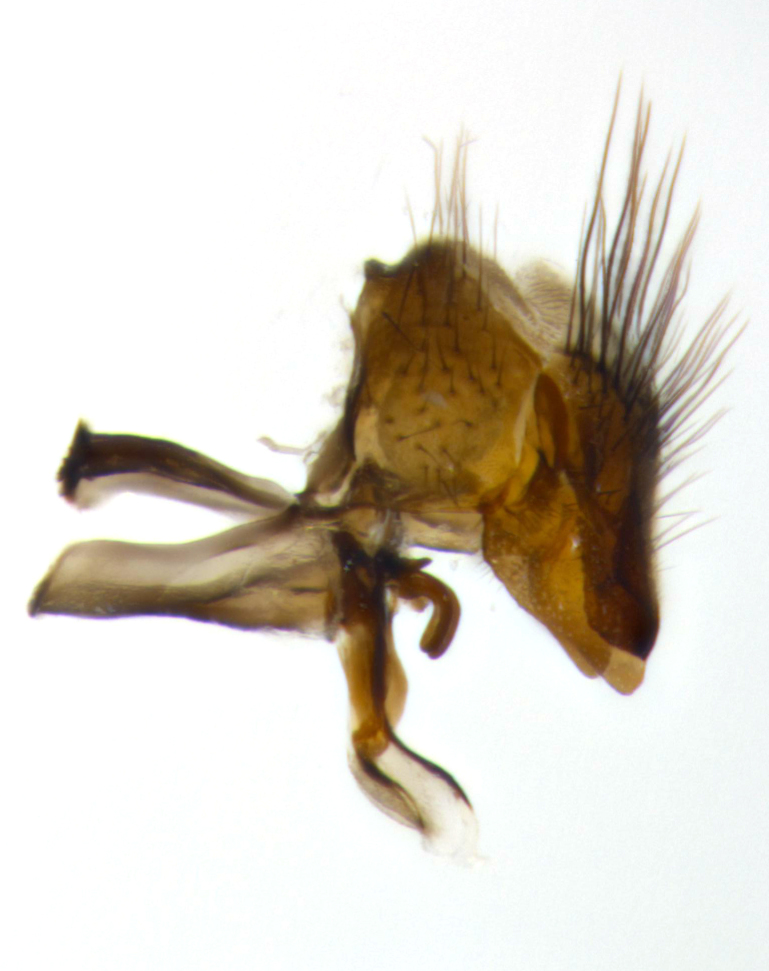
terminalia in lateral view

**Figure 9c.**
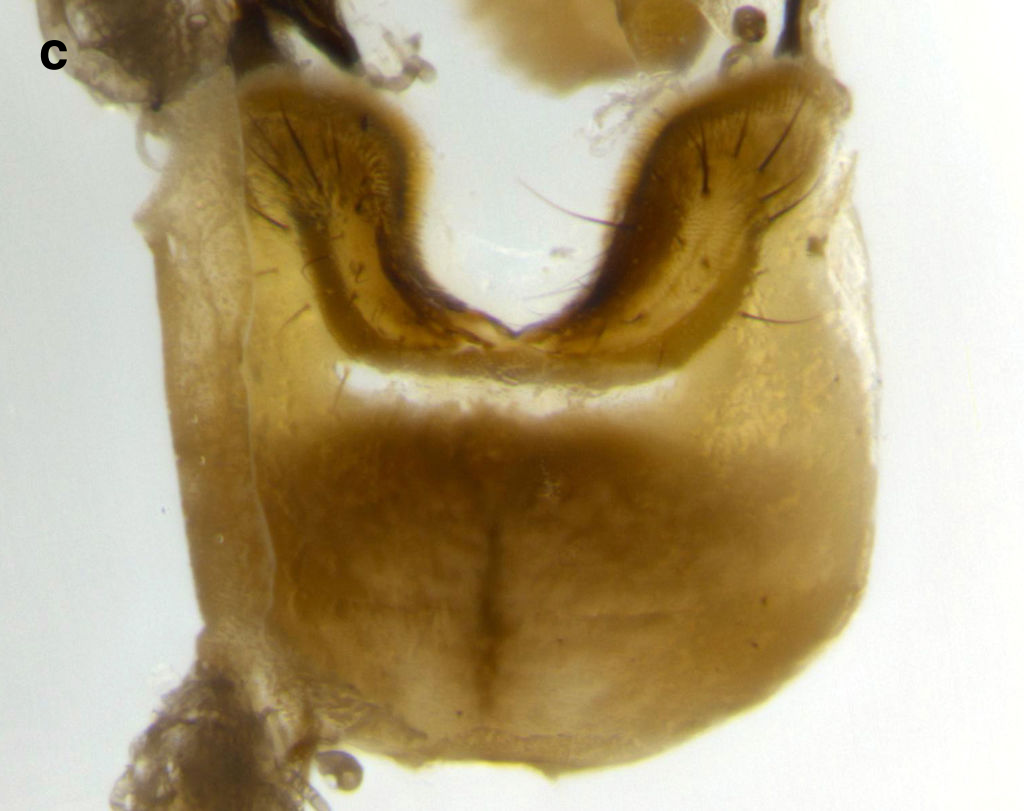
sternite 5 in ventral view

**Figure 10a.**
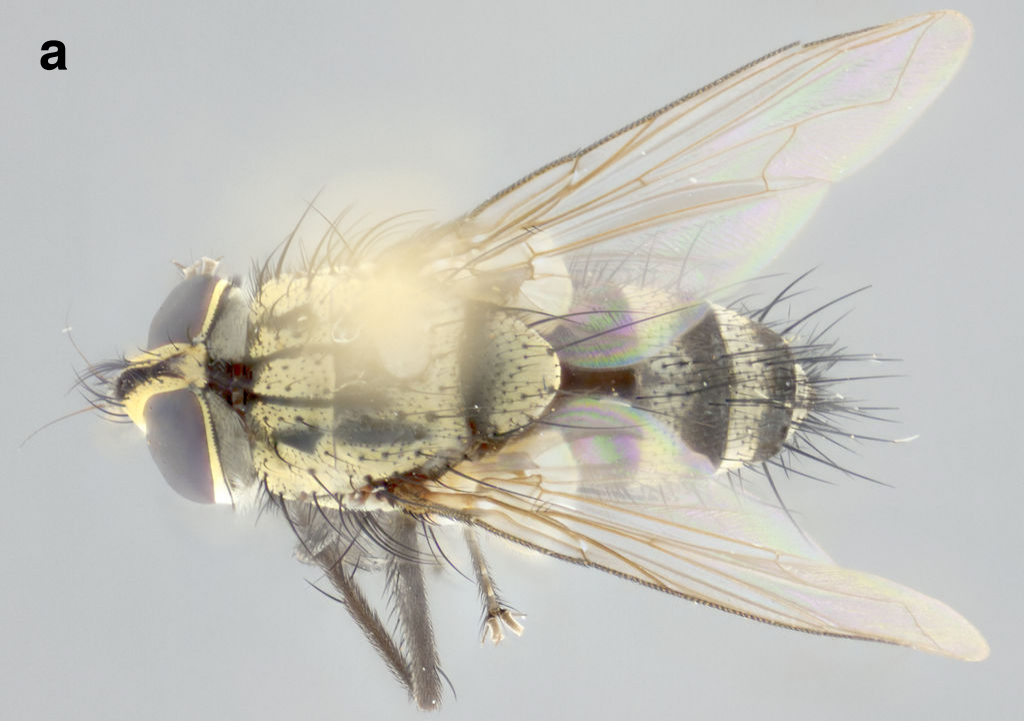
habitus in dorsal view

**Figure 10b.**
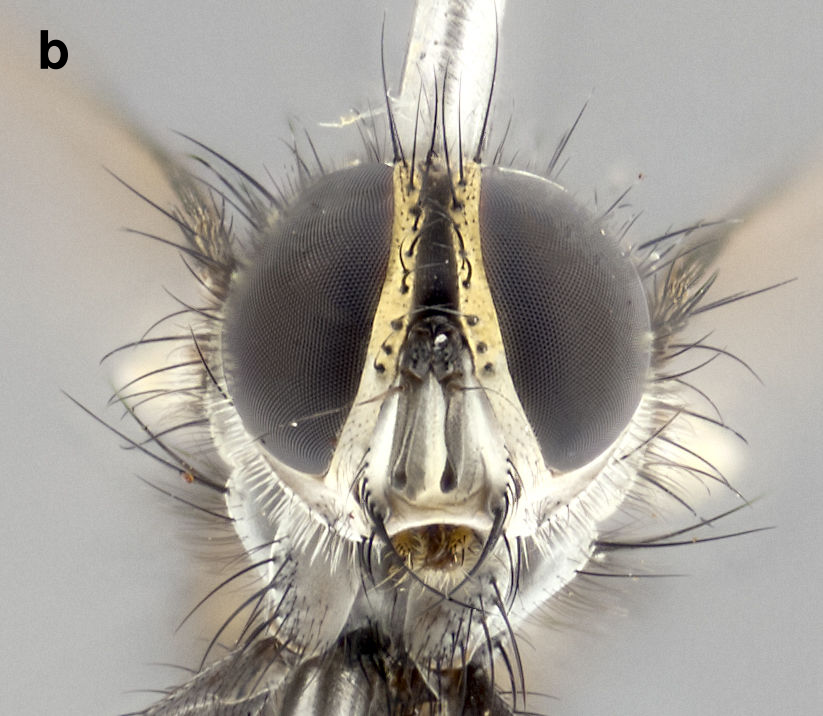
head in frontal view

**Figure 10c.**
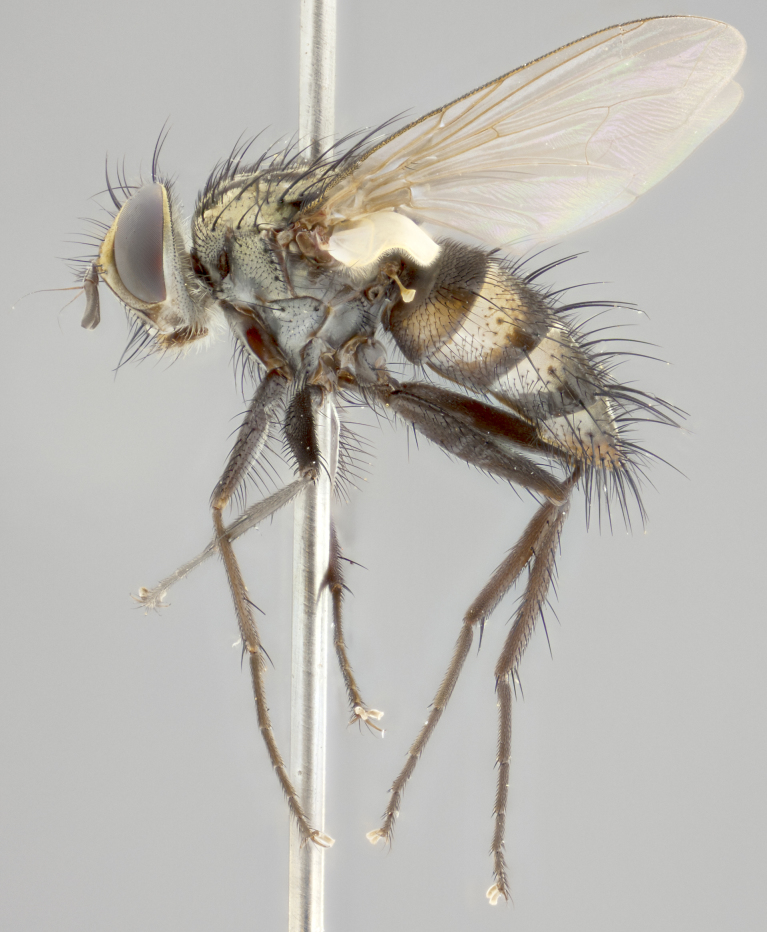
habitus in lateral view

**Figure 10d.**
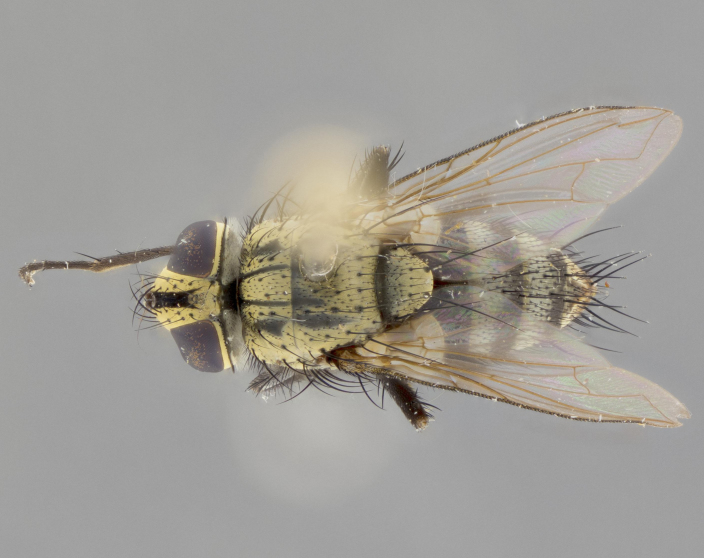
habitus in dorsal view

**Figure 10e.**
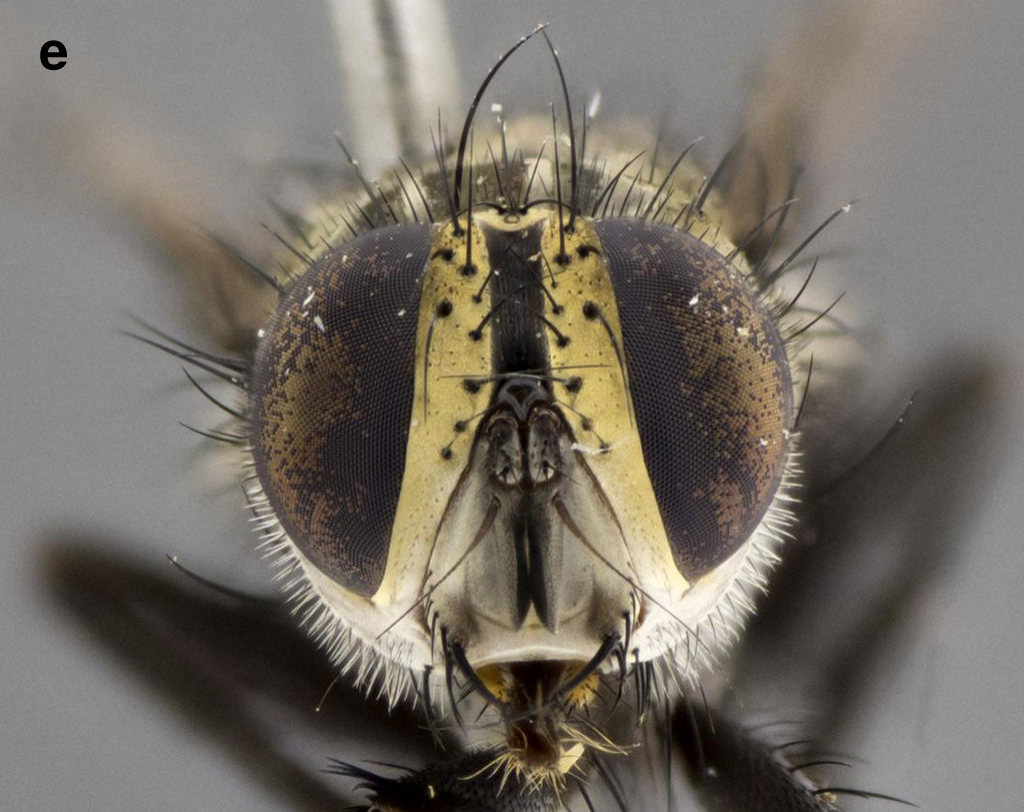
head in frontal view

**Figure 10f.**
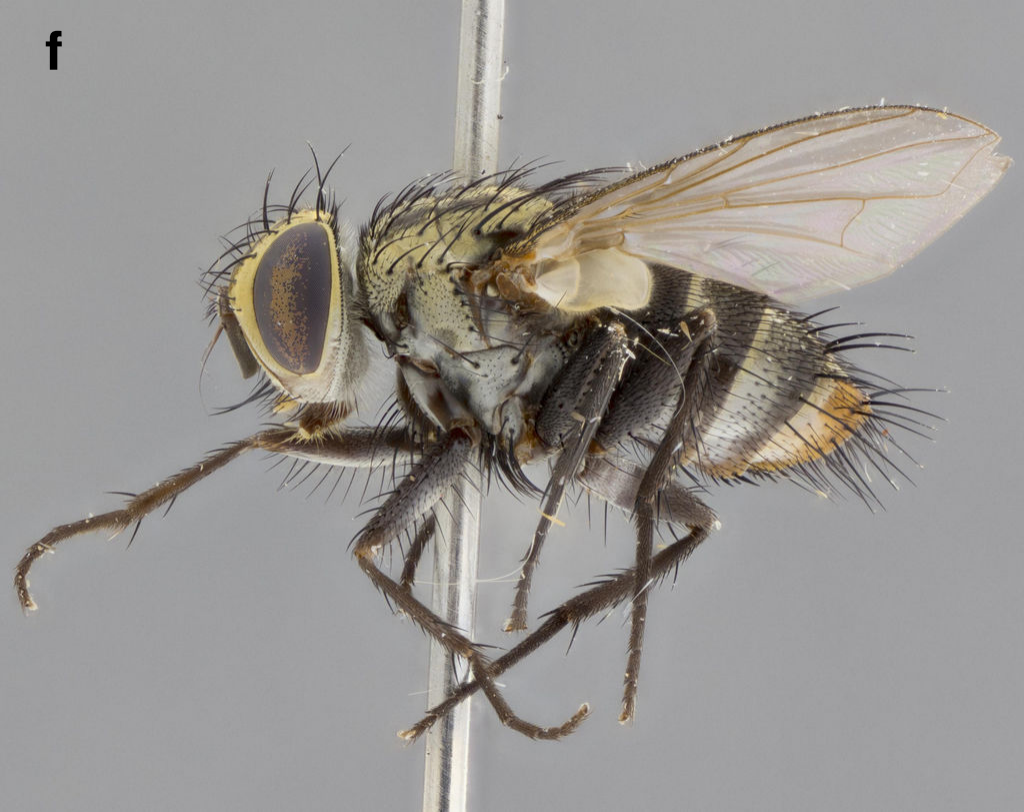
habitus in lateral view

**Figure 11a.**
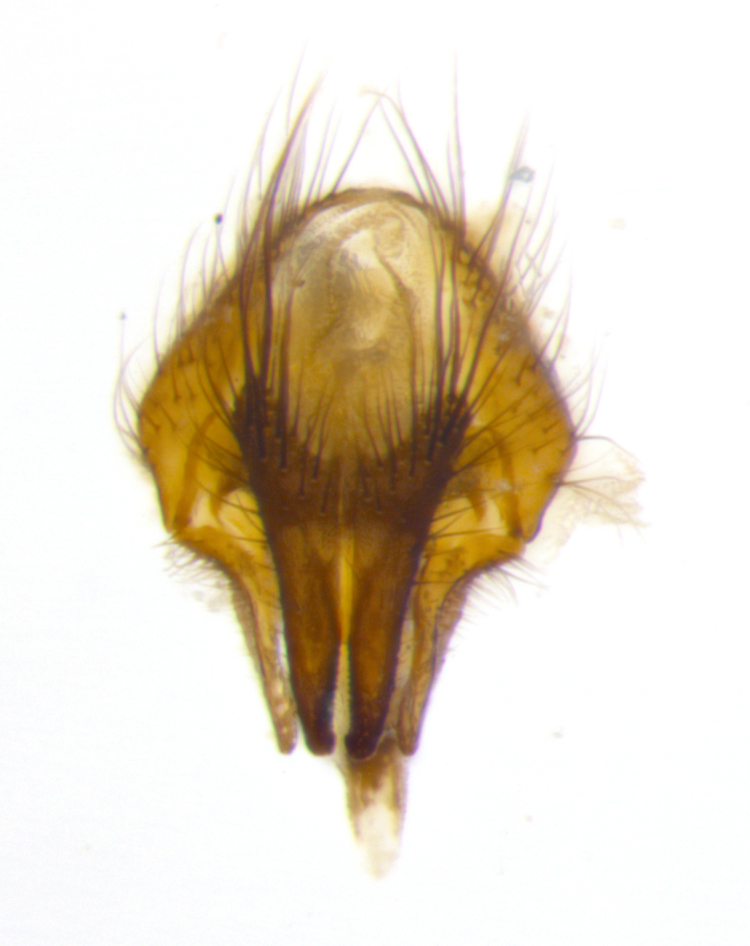
terminalia in dorsal view

**Figure 11b.**
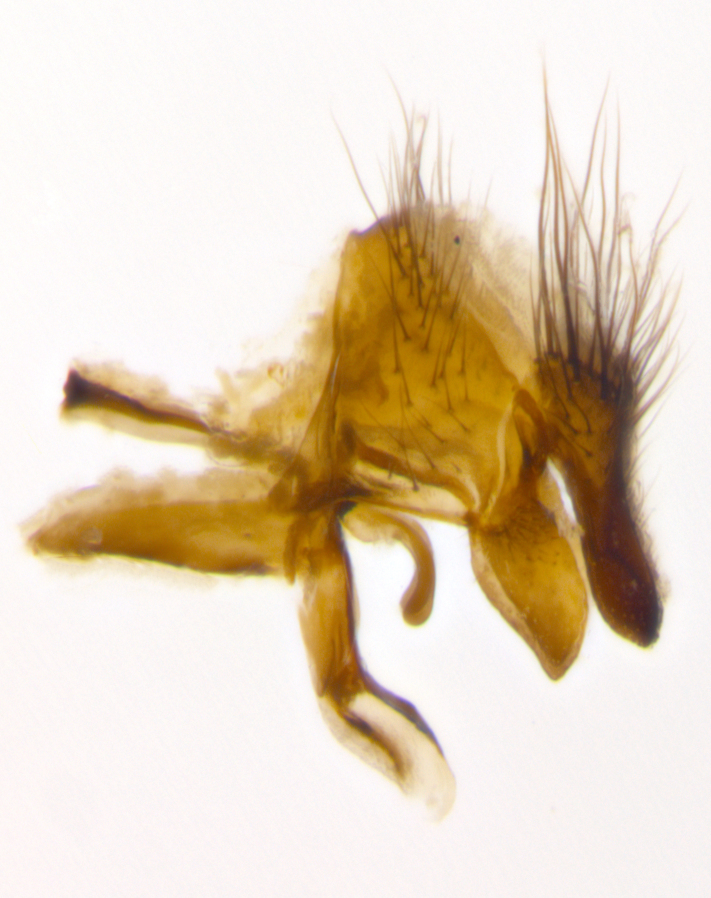
terminalia in lateral view

**Figure 11c.**
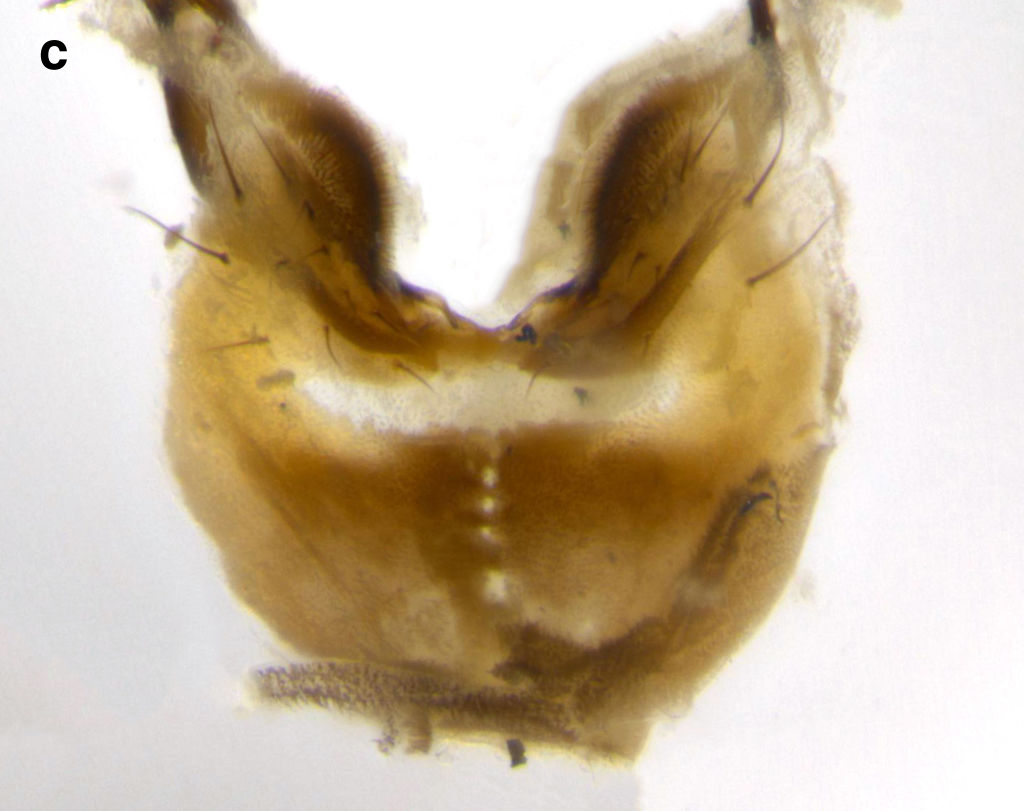
sternite 5 in ventral view
